# The efficacy and safety of Chaihu guizhi ganjiang tang for type 2 diabetes mellitus: a systematic review and meta-analysis

**DOI:** 10.3389/fphar.2026.1855616

**Published:** 2026-06-25

**Authors:** Quan Zhang, Yujie Ouyang, Shurui He, Chunguang Xie, Xiaoxu Fu

**Affiliations:** 1 Chengdu University of Traditional Chinese Medicine, Chengdu, China; 2 Hospital of Chengdu University of Traditional Chinese Medicine, Chengdu, China; 3 TCM Prevention and Treatment of Metabolic and Chronic Diseases Key Laboratory of Sichuan Province, Hospital of Chengdu University of Traditional Chinese Medicine, Chengdu, China; 4 Department of Endocrinology, Hospital of Chengdu University of Traditional Chinese Medicine, Chengdu, China

**Keywords:** Chaihu guizhi ganjiang tang, insulin resistance, meta-analysis, traditional Chinese medicine, type 2 diabetes mellitus

## Abstract

**Background:**

The pathological mechanisms of type 2 diabetes mellitus (T2DM) are complex and necessitate multi-target intervention strategies. Chaihu Guizhi Ganjiang Tang (CHGZGJT), a classical formula derived from the ancient canonical text *Shanghan Lun* (Treatise on Cold Damage Diseases), has been reported to improve glucose and lipid metabolism as well as islet function in patients with T2DM. However, a quantitative evidence-based evaluation is currently lacking. This study aimed to systematically evaluate the overall efficacy and safety of CHGZGJT.

**Methods:**

This study was registered on the PROSPERO platform. Eight Chinese and English databases and two clinical trial registries were systematically searched, and 12 clinical controlled trials involving a total of 883 patients were ultimately included. Meta-analysis were performed using R software to assess efficacy and safety.

**Results:**

The meta-analysis suggested that CHGZGJT combined with conventional treatment significantly reduced glycated hemoglobin (HbA1c, MD = −0.69%), fasting plasma glucose (FPG, MD = −0.86 mmol/L), and 2-h postprandial glucose (2hPG, MD = −0.74 mmol/L) in patients with T2DM. At the mechanistic level, this regimen significantly decreased fasting insulin (FINS, MD = −2.08 µIU/mL) and the homeostasis model assessment of insulin resistance (HOMA-IR, MD = −0.79), improved islet β-cell function (HOMA-β, MD = 5.13), and lowered total cholesterol, triglyceride, and low-density lipoprotein cholesterol levels, with a lower risk of adverse events (RR = 0.44).

**Conclusion:**

CHGZGJT may improve glycemic control, insulin resistance, β-cell function, and the lipid profile in patients with T2DM, with a favorable safety profile for short-term administration. However, the quality of evidence from the included studies was low to moderate, and high-quality clinical trials are still warranted to further verify its long-term efficacy and safety.

## 1 Introduction

Type 2 diabetes mellitus (T2DM) has emerged as one of the most critical global public health challenges of the 21st century, characterized by a sustained and alarming escalation in prevalence. According to the latest data from the International Diabetes Federation, the number of adults aged 20–79 living with diabetes globally reached 589 million in 2024, representing a prevalence of 11.1%. Driven largely by population growth, aging, and urbanization trends, this number is projected to surge to 853 million by 2050 (Duncan et al., 2025). Similarly, the Global Burden of Disease study revealed that the age-standardized global prevalence of diabetes was 6.1% in 2021 and is anticipated to rise to 9.8% by 2050 (Kanyin Liane et al., 2023). This disease not only inflicts severe damage on individual health but also imposes a substantial socioeconomic burden. In 2024, diabetes-related health expenditure reached one trillion USD, accounting for 12% of global health spending, while causing over 3.4 million deaths, representing 9.3% of global mortality (Duncan et al., 2025). As the predominant form of diabetes (accounting for over 96% of cases), the complexity of the pathophysiology of T2DM and the diversity of its complications constitute the central difficulties in disease management (Kanyin Liane et al., 2023).

With respect to its pathophysiological basis, the core characteristics of T2DM lie in insulin resistance (IR) and progressive pancreatic β-cell dysfunction, which are intertwined and collectively bioactive constituents drive the collapse of glucose homeostasis. IR not only manifests as reduced responsiveness to insulin in target tissues (e.g., liver, skeletal muscle, adipose tissue) but also involves complex molecular mechanisms, including the accumulation of ectopic lipids (particularly diacylglycerol) activating protein kinase C ε/θ to interfere with insulin signaling, endoplasmic reticulum stress, and abnormalities in the hexosamine biosynthetic pathway (Wondmkun, 2020; Lee et al., 2022; Dludla et al., 2023). Among these, the concept of “selective insulin resistance” is particularly critical; under conditions of hyperinsulinemia, the ability of insulin to suppress hepatic gluconeogenesis is impaired, yet its ability to stimulate hepatic lipogenesis remains preserved, directly leading to the coexistence of hyperglycemia, hyperlipidemia, and hepatic steatosis (Lee et al., 2022). Simultaneously, under the persistent assault of hyperglycemia and oxidative stress, the antioxidant defense system of β-cells collapses, and key transcription factors (e.g., PDX1, MafA) are suppressed, resulting in functional failure and apoptosis (Park et al., 2025). Furthermore, a chronic low-grade inflammatory state characterized by elevated C-reactive protein (CRP), interleukin-6 (IL-6), and tumor necrosis factor-α (TNF-α) persists throughout the disease process, exacerbating IR and directly damaging β-cells (Dludla et al., 2023; Pellegrini et al., 2024; Atak Tel et al., 2025). If this metabolic derangement is not effectively curbed, it will trigger long-term microvascular (e.g., nephropathy, retinopathy) and macrovascular complications (e.g., myocardial infarction, stroke) through the “metabolic memory” effect, with the latter being the primary cause of mortality in patients (Rawshani et al., 2018; Marx et al., 2023; ElSayed et al., 2024b).

To address this complex disease pattern, modern medicine has established comprehensive management strategies ranging from lifestyle interventions and classic agents such as metformin to novel therapeutics including sodium-glucose cotransporter-2 inhibitors and glucagon-like peptide-1 receptor agonists. These interventions aim to reduce cardiovascular-kidney-metabolic risks (Ghimire and Sakiewicz, 2025; Zhu and Guo, 2025). However, despite the expanding therapeutic armamentarium, clinical management continues to face severe challenges. First, therapeutic inertia and low comprehensive compliance rates remain pervasive issues. Real-world data indicate that only approximately 22.2% of patients achieve simultaneous control of glucose, blood pressure, and lipids. Furthermore, significant delays in treatment intensification with a median delay ranging from 1 to 7 years drastically increase the risk of complications (ElSayed et al., 2024a; Rodriguez et al., 2024). Second, existing medications possess varying degrees of limitations. Metformin is prone to causing gastrointestinal discomfort which affects long-term tolerability. Sulfonylureas are associated with risks of hypoglycemia and weight gain while thiazolidinediones may increase the risk of heart failure. Although novel agents offer cardiorenal benefits, their prohibitive costs and the inconvenience of injectable administration limit widespread accessibility (Nabrdalik et al., 2024; Rodriguez et al., 2024). More critically, even when traditional risk factors are controlled, many patients still face threats from cardiovascular events driven by residual inflammatory risk. Additionally, the current uniform treatment models struggle to precisely address the high clinical and pathological heterogeneity of T2DM (Pellegrini et al., 2024; Rodriguez et al., 2024).

These unmet clinical needs have prompted the medical community to turn its attention to Traditional Chinese Medicine (TCM) therapies, which possess advantages in holistic regulation. TCM categorizes T2DM under the domain of “Xiao Ke” (Wasting and Thirsting), emphasizing the underlying imbalance of yin and yang and the dysfunction of the viscera. Unlike chemical drugs targeting single pathways, TCM formulas intervene in the disease process through multi-metabolite synergy, multi-target action, and multiple pathways (Wen et al., 2025). Modern pharmacological studies have suggested that active metabolites in TCM exert effects through various mechanisms. For instance, saikosaponins and 6-gingerol improve lipid metabolism and mitochondrial function by activating the AMPK/MAPK signaling pathway; baicalein directly promotes β-cell survival and secretion; and various active metabolites regulate the PI3K/AKT insulin signaling pathway while inhibiting the NF-κB inflammatory pathway and oxidative stress (Lim et al., 2021; Peng et al., 2024; Wen et al., 2025). Additionally, the potential of TCM to systematically improve the metabolic inflammatory environment by regulating the “gut-liver axis” and gut microbiota structure (e.g., increasing short-chain fatty acid (SCFA)-producing bacteria) is increasingly recognized (Yang et al., 2022; Wu et al., 2024). Within the TCM therapeutic system, Chaihu Guizhi Ganjiang Tang (CHGZGJT), a classic formula from the *Shanghan Lun* (Treatise on Cold Damage Diseases), demonstrates unique application value. Derived from a modification of Xiaochaihu Tang, the formula employs *Bupleuri Radix* (Chaihu) and *Scutellariae Radix* (Huangqin) to harmonize the Shaoyang and clear constrained heat; *Cinnamomi Ramulus* (Guizhi) and *Zingiberis Rhizoma* (Ganjiang) to warm and unblock yang qi and transform fluid retention; and *Trichosanthis Radix* (Tianhuafen) and *Ostreae Concha* (Muli) to generate fluids, astringe yin, and dissipate binding. The formula utilizes both cold and warm properties and combines reinforcing and reducing methods, possessing the efficacy of resolving Shaoyang disorders and warming the middle to transform rheum. In the *Shanghan Lun*, it is primarily used to treat symptoms such as “fullness and slight binding in the chest and hypochondrium, inhibited urination, thirst without vomiting, sweating from the head only, alternating chills and fever, and vexation.” These manifestations bear a high degree of similarity to the clinical presentation of T2DM patients, such as sensation of heat, dry mouth and thirst, mood fluctuations, and metabolic disturbances. Consequently, this formula is widely applied in modern clinical practice for T2DM and has shown favorable efficacy. Recent preclinical studies have further elucidated the multidimensional mechanisms by which CHGZGJT intervenes in T2DM and related metabolic disorders. In animal models of T2DM, CHGZGJT significantly improved fasting blood glucose levels and attenuated the progressive elevation of glycated hemoglobin (HbA1c), with the underlying mechanisms potentially related to the remodeling of gut microbiota structure (including enrichment of *Lactobacillus* and reduction in the relative abundance of *Ruminococcus*) and regulation of SCFA metabolism (Li et al., 2024). In a model of metabolic dysfunction-associated steatohepatitis (MASH), the core bioactive metabolites, including saikosaponin A, baicalin, and wogonoside, exerted dual lipid-regulating and anti-inflammatory effects by activating PPARα-mediated fatty acid oxidation pathways (upregulating CPT1A, FABP1, and ACOX1) and inhibiting TLR4/MyD88/NF-κB inflammatory signaling (Wu et al., 2026). Multi-omics analysis integrating gut microbiome profiling, metabolomics, and transcriptomics further demonstrated that CHGZGJT enriched beneficial microbial genera including *Lactobacillus* and *Lachnoclostridium*, upregulated intestinal tight junction proteins (ZO-1, Occludin, and Claudin-1) to restore intestinal barrier integrity, and cooperatively activated PPARα signaling while suppressing TLR4/NF-κB signaling, thereby attenuating hepatic steatosis, inflammation, and fibrosis (Wu et al., 2024). Collectively, the aforementioned preclinical evidence indicates that CHGZGJT exerts multisystem interventional potential in models of T2DM and its metabolic comorbidities by modulating the gut microbiota–SCFA metabolic axis, activating key lipid metabolic pathways, and inhibiting metabolic inflammation at multiple targets, thereby providing a solid mechanistic foundation for the clinical evidence-based analysis presented in this study.

However, despite the promising theoretical and preclinical evidence, high-level evidence-based medical data on CHGZGJT for T2DM remain scarce. Existing clinical studies are mostly small-sample trials characterized by inadequate reporting of randomization and blinding methods, and considerable design heterogeneity (variations in dosage, treatment duration, and control interventions). More critically, current research predominantly focuses on short-term glycemic changes, lacking systematic evaluation of deeper metabolic indicators such as HOMA-IR and HOMA-β, systemic inflammatory markers, and long-term safety. This fragmented evidence landscape prevents clinicians from forming definitive quantitative conclusions and hinders the standardized application of this formula. Therefore, this study aimed to quantitatively synthesize data from existing clinical trials *via* a systematic review and meta-analysis to clarify the efficacy and safety of CHGZGJT in improving glucolipid metabolism, insulin function, and inflammatory markers, thereby providing the first comprehensive, quantitative evidence-based medical evidence to inform clinical practice and guide future research.

## 2 Materials and methods

### 2.1 Study design and registration

This study was designed and conducted as a systematic review and meta-analysis to evaluate the efficacy and safety of CHGZGJT combined with conventional treatment in patients with T2DM. The design and reporting of the research protocol strictly adhered to the Preferred Reporting Items for Systematic Reviews and Meta-Analysis (PRISMA) 2020 statement. The protocol was prospectively registered with the International Prospective Register of Systematic Reviews (PROSPERO) under registration number CRD420261294373.

### 2.2 Literature search strategy

To ensure systematic and unbiased literature acquisition, a comprehensive search strategy was developed covering four English databases (PubMed, Embase, Cochrane Library, and Web of Science) and four Chinese databases (China National Knowledge Infrastructure [CNKI], Wanfang Data, VIP Chinese Science and Technology Periodicals Database, and SinoMed [CBM]). Additionally, ClinicalTrials.gov and the Chinese Clinical Trial Registry (ChiCTR) were searched. The search period extended from the inception of each database to 24 January 2026, to fully cover all potential studies since the field’s initiation. The search logic employed a combination of Medical Subject Headings (MeSH) and free-text terms. English search terms included “Chaihu guizhi ganjiang tang,” “Chaihu-guizhi-ganjiang-tang,” “Chai hu gui zhi gan jiang tang,” “type 2 diabetes,” “noninsulin-dependent diabetes mellitus,” and “T2DM”. Detailed search strategies and queries are provided in Supplementary Material S2.

### 2.3 Inclusion and exclusion criteria

This study established strict inclusion and exclusion criteria based on the PICO principle. Regarding study types, randomized controlled trials (RCTs) and non-randomized clinical controlled trials were included, while case reports, case series, reviews, animal experiments, *in vitro* studies, and conference abstracts lacking specific data were excluded. Regarding participants, the study included patients aged 18 years or older who met authoritative diagnostic criteria for T2DM, such as those of the World Health Organization or the *Guideline for the Prevention and Treatment of Type 2 Diabetes Mellitus in China*; patients with type 1 diabetes, gestational diabetes, secondary diabetes, or acute severe metabolic complications (e.g., diabetic ketoacidosis or hyperosmolar hyperglycemic state) were excluded. In terms of interventions and control settings, the experimental group was defined as receiving CHGZGJT or its clinically common modified formulas (regardless of dosage form) combined with conventional biomedical treatment; the control group received the exact same conventional biomedical treatment as the experimental group, including diabetes health education, dietary control, exercise guidance, and guideline-recommended oral hypoglycemic agents or insulin therapy. If the intervention in the experimental group included other TCM therapies (e.g., acupuncture) in addition to CHGZGJT, the study was excluded. Regarding outcome indicators, included studies were required to report at least one pre-specified indicator, including glucose metabolism metrics such as HbA1c, fasting plasma glucose (FPG), and 2-h postprandial glucose (2hPG); lipid metabolism metrics including total cholesterol (TC), triglycerides (TG), low-density lipoprotein cholesterol (LDL-C), and high-density lipoprotein cholesterol (HDL-C); insulin function metrics such as fasting insulin (FINS), homeostasis model assessment of insulin resistance (HOMA-IR), and homeostasis model assessment of β-cell function (HOMA-β); as well as C-reactive protein (CRP), clinical total effective rate, and the incidence of adverse events.

### 2.4 Literature screening and data extraction

Literature obtained through database searching was imported into EndNote X9 software for unified management, and duplicate records were removed using a combination of automatic software deduplication and manual verification. Literature screening was conducted independently by investigators QZ and OUY in two stages. First, studies that clearly did not meet the inclusion criteria were excluded by reading titles and abstracts. Second, full texts of the literature passing the initial screening were retrieved and re-evaluated against the PICO criteria. Any disagreements during the screening process were resolved through discussion; if a consensus could not be reached, investigators SH and XF were consulted for adjudication. Data extraction was similarly performed independently by QZ and OUY using a pre-designed standardized form, followed by cross-verification. The extracted content covered basic information such as the first author, publication year, study design, and sample size; characteristic information including patient age, disease duration, and baseline metabolic levels; intervention details such as the composition, dosage, and treatment duration of CHGZGJT, and the control group regimen; as well as data on outcome indicators, including means, standard deviations, or event counts, and details of adverse reactions. For outcome measures reported in different units across the included studies, unit conversion was performed following data extraction to ensure consistency. Specifically, fasting insulin (FINS) was reported in units of μIU/mL, μU/mL, and U/L. According to the International System of Units and clinical laboratory standards, 1 μIU/mL is equivalent to 1 μU/mL. For studies reporting values in U/L, the original numerical ranges were verified and compared with those of the remaining studies, and it was determined that this unit in fact denoted μIU/mL (i.e., 1 U/L = 1 μIU/mL). Accordingly, no numerical conversion was performed. The remaining indicators (FPG, 2hPG, HbA1c, TC, TG, LDL-C, HDL-C, and CRP) were reported in consistent units across all studies and required no conversion.

### 2.5 Botanical nomenclature verification and characterization of botanical drug interventions

In accordance with the best-practice standards of the Society for Medicinal Plant and Natural Product Research (GA) and the consensus recommendations for reporting natural product research (Heinrich et al., 2022), the botanical identity and level of characterization of each botanical drug intervention were verified in the present study. For each constituent medicinal material reported in the included trials, the scientific name, authority citation, and botanical family were validated against the Medicinal Plant Names Services (MPNS) of the Royal Botanic Gardens, Kew, and the Plants of the World Online (POWO) database, and the corresponding pharmaceutical names and medicinal parts were determined in accordance with the Pharmacopoeia of the People’s Republic of China (2020 Edition). For medicinal materials of animal or fungal origin not covered by MPNS or POWO (*oyster*, *earthworm*, *black-tailed snake*, *and Poria*), verification was performed against the World Register of Marine Species, authoritative zoological reference works, and the Index Fungorum database, respectively. The complete nomenclatural information for all constituent medicinal materials following taxonomic verification is provided in Supplementary Appendix S1. As the interventions in the original trials were individually prepared aqueous decoctions rather than standardized extracts, the ConPhYMP framework (Heinrich et al., 2022) was further employed to evaluate the level of chemical characterization reported in the original studies.

### 2.6 Risk of bias assessment

Risk of bias assessment was performed independently by investigators QZ and OUY. For RCTs, the Cochrane risk of bias tool 2.0 (RoB 2) was employed to evaluate risk levels across five domains: randomization process, deviations from intended interventions, missing outcome data, measurement of the outcome, and selection of the reported result. For non-randomized controlled studies, the risk of bias in non-randomized studies of interventions (ROBINS-I) tool was used, covering seven domains: bias due to confounding, selection of participants, classification of interventions, deviations from intended interventions, missing data, measurement of outcomes, and selection of the reported result. Discrepancies in assessment were resolved through arbitration by SH and XF. Final assessment results were presented in tabular format and risk summary diagrams.

### 2.7 Quantitative synthesis and statistical analysis

The meta-analysis was conducted using R software (version 4.4.2) with the meta and metafor packages. Continuous variables such as HbA1c and FPG were analyzed using mean difference (MD) as the effect size while dichotomous variables including the clinical total effective rate and incidence of adverse events were analyzed using the risk ratio (RR). The 95% confidence intervals were calculated for all effect sizes. Heterogeneity among studies was quantified using the I^2^ statistic and Cochrane Q test *P*-value. If I^2^ ≤ 50% and the Q test *P* > 0.05, heterogeneity was considered acceptable, and a fixed-effects model was used; otherwise, a random-effects model was applied. To investigate sources of heterogeneity, subgroup analysis were pre-specified for high-heterogeneity outcomes based on treatment duration, age, disease duration, and the presence of comorbidities. Meta-regression analysis was performed to test linear relationships between these covariates and effect sizes. Robustness of results was verified using sensitivity analysis *via* the leave-one-out method. For outcomes with 10 or more included studies, publication bias was assessed using funnel plots and Egger’s linear regression test, with *P* < 0.05 indicating statistically significant publication bias. It should be noted that the included studies exhibited a certain degree of cross-study variation in the specific composition of CHGZGJT (including the dosages of individual medicinals and the medicinals added or subtracted according to syndrome differentiation) as well as in the conventional biomedical background treatment regimens administered to the control groups (encompassing various regimens and dosages of metformin, sulfonylureas, sodium-glucose cotransporter 2 (SGLT-2) inhibitors, and insulin). The aforementioned clinical heterogeneity was regarded as a potential source of between-study variation in effect measures and was systematically evaluated through random-effects models, subgroup analysis, meta-regression, and sensitivity analysis.

## 3 Results

### 3.1 Literature search and screening results

**FIGURE 1:**
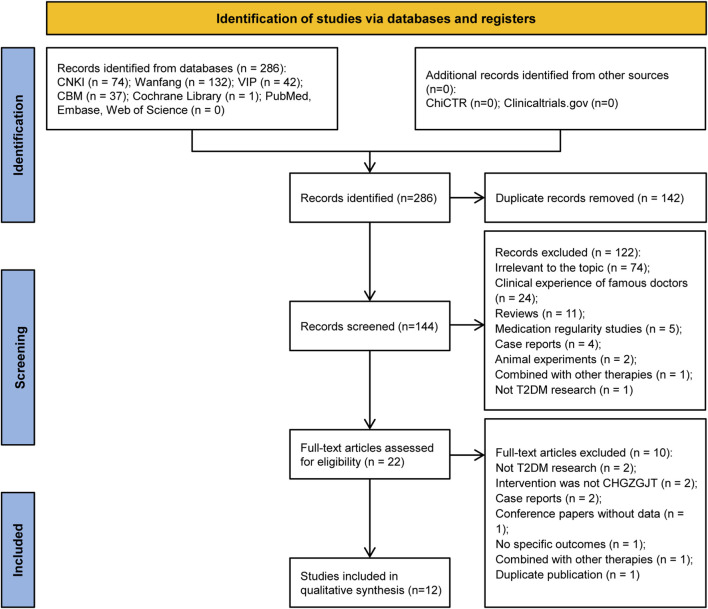
Flow diagram of study selection process.

A total of 286 relevant records were initially identified through a systematic search of English and Chinese databases as well as clinical trial registries. Specifically, 285 records were retrieved from Chinese databases, including 74 from the CNKI, 132 from Wanfang data, 42 from the VIP, and 37 from the CBM. Regarding English databases, 1 record was identified in the Cochrane Library, while no relevant literature was retrieved from PubMed, Embase, or Web of Science. Similarly, no relevant studies were found in the two clinical trial registries. Following deduplication using EndNote X9 software supplemented by manual verification, 142 duplicate records were removed, leaving 144 records for preliminary screening. By reviewing titles and abstracts, 122 records that clearly did not meet the inclusion criteria were excluded. The reasons for exclusion included irrelevance to the topic (n = 74), summaries of renowned physicians’ clinical experience (n = 24), reviews (n = 11), studies on medication rules (n = 5), case reports (n = 4), animal experiments (n = 2), combination with other therapies (n = 1), and non-T2DM research (n = 1). After initial screening, the remaining 22 articles underwent full-text review. Based on the pre-specified inclusion and exclusion criteria, 10 additional articles were excluded upon strict full-text examination. The reasons for exclusion were as follows: non-T2DM studies (n = 2), intervention other than CHGZGJT (n = 2), case reports (n = 2), conference literature lacking specific data (n = 1), absence of specific study results (n = 1), combination with other therapies (n = 1), and duplicate publication (n = 1). Ultimately, a total of 12 studies meeting the criteria were included in this meta-analysis. The detailed process of literature retrieval and screening is illustrated in Figure 1.

### 3.2 Characteristics of included studies

A total of 12 clinical controlled trials were ultimately included in this study. All trials were conducted in China, with publication dates ranging from 2009 to 2025. These studies involved a combined total of 883 patients with T2DM, comprising 440 in the experimental group and 443 in the control group. Regarding study design, 10 studies were randomized controlled trials (RCTs) (Wang and Xiao, 2009; Zhang, 2014; Mao and Wang, 2017; Li, 2019; Zhao, 2022; Zhao, 2023; Zheng and Ma, 2023; Xiang et al., 2024; Zhang, 2024; Rong et al., 2025), while the remaining two studies were non-randomized clinical controlled studies (Lv et al., 2019; Liu, 2022). In terms of diagnostic criteria, the included studies primarily relied on authoritative international or domestic guidelines. Specifically, six studies adopted the World Health Organization (WHO) diagnostic criteria for diabetes (1999 or 2010 editions) (Wang and Xiao, 2009; Zhang, 2014; Mao and Wang, 2017; Lv et al., 2019; Liu, 2022; Zhao, 2022), with some also referencing American Diabetes Association (ADA) standards. Four studies based their diagnosis on the *Guideline for the Prevention and Treatment of Type 2 Diabetes Mellitus in China* (2017 or 2020 editions) (Zheng and Ma, 2023; Xiang et al., 2024; Zhang, 2024; Rong et al., 2025). The remaining two studies did not specify the guideline version but stated adherence to clinical diagnostic criteria for T2DM (Li, 2019; Zhao, 2023). Regarding interventions, all control groups received conventional biomedical treatment, which included diabetes health education, dietary control, exercise guidance, and oral hypoglycemic agents or insulin therapy. The experimental groups received CHGZGJT in combination with the treatment provided to the control groups. With respect to botanical drug composition, one included study utilized the original CHGZGJT formula (Zhao, 2022), while the remaining 11 studies employed modified CHGZGJT formulas (Wang and Xiao, 2009; Zhang, 2014; Mao and Wang, 2017; Li, 2019; Lv et al., 2019; Liu, 2022; Zhao, 2023; Zheng and Ma, 2023; Xiang et al., 2024; Zhang, 2024; Rong et al., 2025). These modifications involved adjustments to the botanical drugs or dosages based on patients’ individualized concurrent syndromes (e.g., qi deficiency, blood stasis, or damp-heat). The complete composition of each formula, along with the verified botanical/pharmacological names and medicinal parts of each botanical drug, are provided in Supplementary Appendix S1. All trials described their interventions as traditional decoctions (“decocted and taken orally”), but none reported the origin of the crude drugs, identification information, or processing methods. Furthermore, they did not provide the decoction ratio, any chemical fingerprint profiles, quantitative data on marker metabolites, or quality control data for the finished decoction. Treatment duration varied across studies, ranging from 4 to 12 weeks. Specifically, one study had a duration of 28 days (4 weeks) (Zhao, 2023); five studies had a duration of 56 days (8 weeks) (Wang and Xiao, 2009; Zhang, 2014; Mao and Wang, 2017; Zhao, 2022; Rong et al., 2025); three studies had a duration of 84 days (12 weeks) (Lv et al., 2019; Liu, 2022; Xiang et al., 2024); and three studies had a duration of 90 days (Li, 2019; Zheng and Ma, 2023; Zhang, 2024). Detailed baseline characteristics of the included studies are summarized in Table 1.

**TABLE 1:** Characteristics of the included studies.

Study	Sample size (T/C)	Gender (M/F)	Age (years)	Course of disease (years)	Duration	Co-intervention (Control group)	Intervention (Treatment group)	Outcome index	Comorbidity
Wang and Xiao (2009)	63/63	T: 36/27 C: 35/28	T: 49.80±0.91 C: 50.10±0.53	14	8 weeks	Xuezhikang capsules + Lifestyle intervention	Modified CHGZGJT, 1 dose/day + CG	①②③⑦⑧⑨⑩⑫	Hyperlipidemia
Zhang (2014)	30/30	T: 18/12 C: 16/14	T: 48.60±7.20 C: 50.70±8.10	T: 10.70±2.10 C: 11.30±2.50	8 weeks	Hypoglycemic drugs (adjusted by glucose levels) + Lifestyle intervention	Modified CHGZGJT, 1 dose/day + CG	①②③⑦⑧⑨⑩⑫	Hyperlipidemia
Mao and Wang (2017)	30/30	T: 12/18 C: 13/17	T: 53.38±5.64 C: 54.08±5.51	16.5	8 weeks	Hypoglycemic drugs or insulin + Lifestyle intervention	Modified CHGZGJT, 1 dose/day + CG	①②③⑫	Diarrhea, Hypertension, CHD, Dyslipidemia
Li (2019)	33/33	T: 15/18 C: 20/13	T: 50.97±7.92 C: 51.10±9.33	T: 5.49±2.03 C: 5.21±1.96	3 months	Metformin tablets, 0.5 g, tid + Lifestyle intervention	Modified CHGZGJT, 1 dose/day + CG	①②③④⑤⑦⑧⑨⑩⑫	NR
Lv et al. (2019)	45/45	T: 25/20 C: 27/18	T: 50.64±7.29 C: 50.82±6.33	T: 5.95±3.32 C: 5.88±3.38	12 weeks	Insulin or Sulfonylureas + Lifestyle intervention	Modified CHGZGJT, 1 dose/day + CG	①②③⑪⑫⑬	NR
Liu (2022)	31/31	T: 17/14 C: 15/16	T: 51.48±14.25 C: 56.41±10.42	T: 7 (3, 15) C: 5 (2, 13)	12 weeks	Insulin, Sulfonylureas, or Biguanides + Lifestyle intervention	Modified CHGZGJT, 1 dose/day + CG	①②③⑫	NR
Zhao (2022)	33/36	T: 15/18 C: 15/21	T: 55.09±8.13 C: 57.39±5.38	T: 4.48±1.83 C: 4.81±1.27	8 weeks	Metformin tablets, 0.5 g, tid + Lifestyle intervention	Original CHGZGJT, 1 dose/day + CG	①②③④⑤⑦⑧⑨⑩⑫	NR
Zhao (2023)	30/30	T: 18/12 C: 17/13	T: 52.50±6.44 C: 53.50±7.45	T: 4.28±1.94 C: 3.90±2.06	4 weeks	Metformin tablets, 0.5 g, tid	Modified CHGZGJT, 1 dose/day + CG	①②③④⑤⑦⑧⑨⑩⑫	NR
Zheng and Ma (2023)	30/30	T: 17/13 C: 18/12	T: 51.79±7.86 C: 51.74±7.82	T: 6.15±2.50 C: 6.22±2.56	3 months	Hypoglycemic drugs/insulin + Cetirizine tablets, 10 mg, qd	Modified CHGZGJT + Guizhi Fuling Pills + CG	①②③⑪⑫⑬	Pruritus
Xiang et al. (2024)	43/43	T: 19/24 C: 17/26	T: 60.28±5.98 C: 60.31±5.44	T: 4.86±0.55 C: 4.71±0.57	12 weeks	Metformin tablets, 0.5-1.0 g, bid + Lifestyle intervention	Modified CHGZGJT, 1 dose/day + CG	①②③⑫	Hypertension, Hyperlipidemia
Zhang (2024)	40/40	T: 14/26 C: 18/22	T: 42.47±3.38 C: 42.98±3.02	T: 3.23±0.18 C: 3.25±0.17	3 months	Metformin tablets, 0.5 g, bid + Diet control	Modified CHGZGJT, 1 dose/day + CG	①②③⑤⑥⑫⑬	NR
Rong et al. (2025)	32/32	T: 18/14 C: 17/15	T: 58.30±9.90 C: 59.70±10.50	T: 3.60±1.80 C: 3.50±1.80	8 weeks	Metformin tablets, 0.5 g, tid + Dapagliflozin tablets, 10 mg, qd + Lifestyle intervention	Modified CHGZGJT + Zhishu Decoction + CG	①②③⑤⑥⑦⑧⑨⑩⑫	NR

Abbreviations: T, treatment group; C, control group; M, male; F, female; NR, not reported; CG, control group interventions; CHGZGJT, Chaihu Guizhi Ganjiang Tang; CHD, Coronary heart disease; bid, twice daily; tid, three times daily; qd, once daily. Outcome index: ① HbA1c; ② FBG; ③ 2hPG; ④ FINS; ⑤ HOMA-IR; ⑥ HOMA-β; ⑦ TC; ⑧ TG; ⑨ LDL-C; ⑩ HDL-C; ⑪ CRP; ⑫ Overall effective rate; ⑬ Adverse events rate.

### 3.3 Risk of bias assessment

**FIGURE 2:**
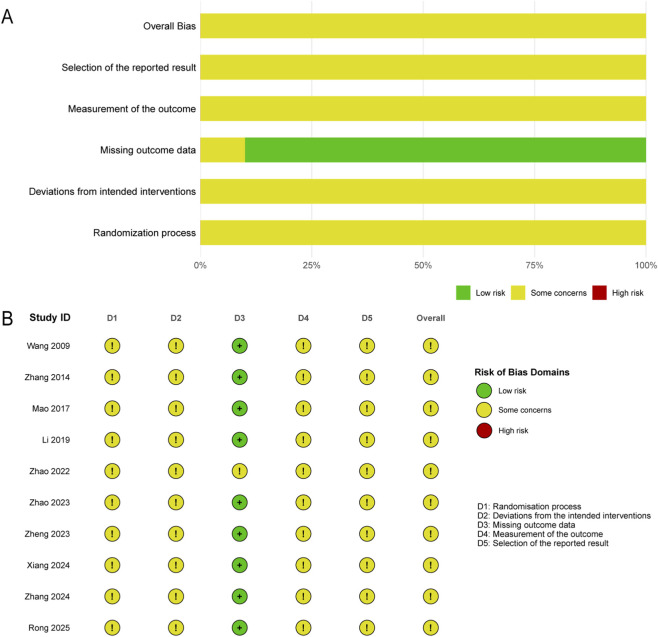
Risk of bias assessment for randomized controlled trials. **(A)** Risk of bias graph; **(B)** Risk of bias summary.

**FIGURE 3:**
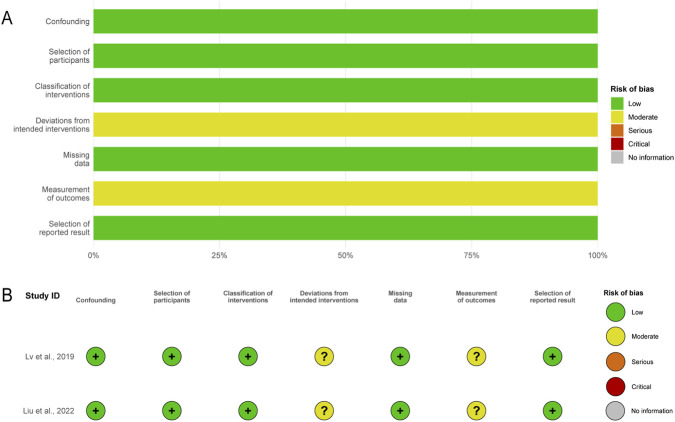
Risk of bias assessment for non-randomized clinical controlled trials. **(A)** Risk of bias graph; **(B)** Risk of bias summary.

The risk of bias for the 10 included randomized controlled trials (RCTs) was systematically evaluated using the Cochrane risk of bias tool 2.0 (RoB 2). The results indicated that the overall risk of bias for all RCTs was classified as “some concerns” (Figure 2). Regarding the randomization process, although some studies mentioned random allocation methods, the lack of specific details on allocation concealment resulted in a classification of “some concerns” for this domain. Concerning deviations from intended interventions and measurement of the outcome, given the specific formulation characteristics of TCM decoctions, none of the included studies implemented placebo controls. Consequently, blinding of participants and personnel was difficult to achieve, and the studies did not explicitly state whether outcome assessors were blinded. Thus, the risk of bias in these two domains was assessed as “some concerns” across all studies. Regarding missing outcome data, the vast majority of studies possessed complete data and were rated as low risk, with the exception of one study which was judged as “some concerns” due to three patient dropouts in the experimental group. For the domain of selection of the reported result, since none of the included studies provided clinical trial registration numbers or pre-published protocols, the possibility of selective reporting could not be completely ruled out. Thus, the risk level for this domain was graded as “some concerns” for all studies. For the two included non-randomized interventional studies, the risk of bias in non-randomized studies of interventions (ROBINS-I) tool was employed. The assessment showed that the overall risk of bias for both studies was at a moderate level (Figure 3). Specifically, both studies demonstrated low risk in five domains which included bias due to confounding, selection of participants, classification of interventions, missing data, and selection of the reported result. However, in the domains of deviations from intended interventions and measurement of outcomes, the risk was classified as moderate due to the inherent limitations of the non-randomized design and the absence of blinding measures. Overall, the methodological quality of the included studies was at a moderate level. Although the lack of blinding implementation is a pervasive challenge in clinical trials of TCM and may introduce implementation and measurement biases, considering that the relevant outcome indicators such as blood glucose, blood lipids, and islet function are objective biochemical markers relatively less influenced by subjective factors, the existing evidence retains reliable reference value.

### 3.4 Primary outcome measures

#### 3.4.1 Glucose metabolism indicators

##### 3.4.1.1 Glycated hemoglobin

**FIGURE 4:**
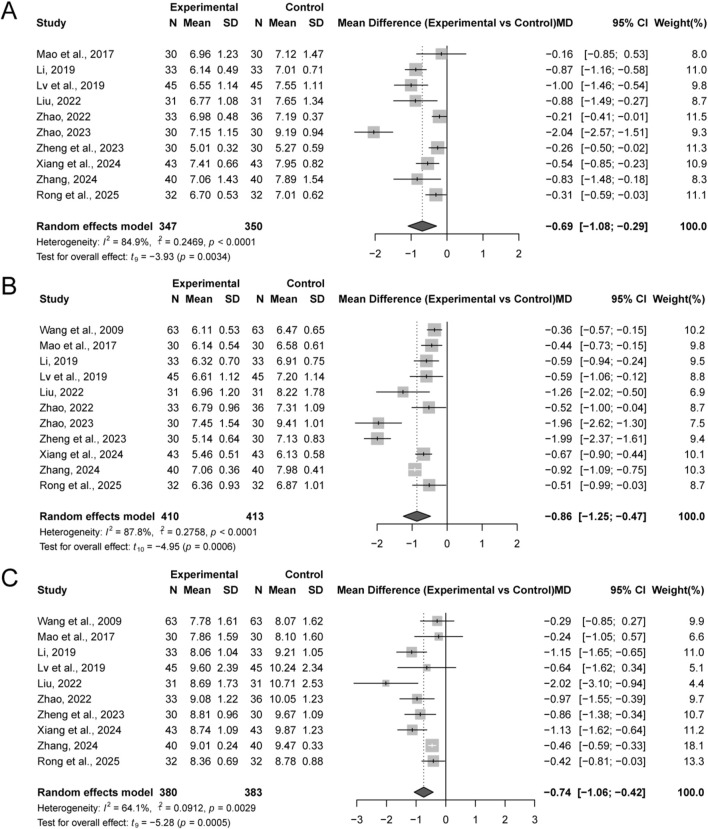
Forest plot for glucose metabolism indicators. **(A)** HbA1c; **(B)** FPG; **(C)** 2hPG.

A total of 10 studies (involving 697 patients) reported changes in HbA1c levels. Heterogeneity testing revealed significant high heterogeneity among the studies (I^2^ = 84.9%, *P* < 0.0001). Therefore, a random-effects model was employed for the pooled analysis. The meta-analysis results indicated that compared with the control group, CHGZGJT combined with conventional therapy significantly reduced HbA1c levels in patients with T2DM, with a pooled MD of −0.69% (95% CI: 1.08 to −0.29, *P* = 0.0034; Figure 4A). This demonstrates a clear clinical advantage of the combined therapy in improving long-term glycemic control. To explore the sources of high heterogeneity, we first constructed a multivariable meta-regression model including age, disease duration, treatment duration, and comorbidities, but found no significant linear relationships between these covariates and the effect size (*P* = 0.5755; Supplementary Material S3). Subsequently, pre-specified subgroup analysis were conducted. Subgroup analysis stratified by age showed a statistically significant inter-group difference (*P* = 0.0312; Supplementary Material S4); in the patient group with a mean age <55 years, the effect of combined therapy on reducing HbA1c was more pronounced (MD = −0.86, 95% CI: 1.42 to −0.30), whereas the effect size was relatively smaller in the population aged ≥55 years (MD = −0.32). Furthermore, the presence of comorbidities was identified as a potential source of heterogeneity (inter-group difference *P* = 0.0464; Supplementary Material S4), with a greater reduction in HbA1c observed in the subgroup without comorbidities (MD = −0.85). Subgroup analysis based on disease duration and treatment duration did not show statistically significant inter-group differences (*P* > 0.05). These findings suggest that the formula may yield the greatest benefit in patients who are relatively younger and have less complex conditions. Sensitivity analysis showed that after sequentially removing any single study, the pooled effect size fluctuated between −0.66% and −0.75%, and the statistical significance remained unchanged, indicating that the primary analysis results are robust (Supplementary Material S5).

##### 3.4.1.2 Fasting plasma glucose

A total of 11 studies (involving 823 patients) provided outcome data for FPG. Due to high heterogeneity among the studies (I^2^ = 87.8%, *P* < 0.0001), a random-effects model was used to pool the data. The meta-analysis results showed that the CHGZGJT combined with conventional therapy group demonstrated a significant advantage in reducing FPG compared with the control group, with a pooled MD of −0.86 mmol/L (95% CI: 1.25 to −0.47, *P* = 0.0006; Figure 4B). This result indicates that the addition of this formula to conventional therapy can further significantly improve FPG control levels in patients. Given the observed high heterogeneity, meta-regression and subgroup analysis were employed to explore potential moderating variables. Meta-regression analysis did not identify significant linear associations between age, disease duration, treatment duration, or comorbidity status and the effect size (*P* > 0.05; Supplementary Material S3). Further subgroup analysis revealed that regardless of whether patients were younger than 55 years (*P* = 0.1285), had a disease duration exceeding 5 years (*P* = 0.9469), had a treatment cycle exceeding 60 days (*P* = 0.4424), or had comorbidities (*P* = 0.9990), the direction of effects remained consistent within each subgroup, and inter-group differences were not statistically significant (Supplementary Material S4). Although the aforementioned clinical characteristics could not fully explain the sources of heterogeneity, the analysis suggests that the efficacy of the formula in improving FPG has good consistency across populations with different characteristics. Sensitivity analysis indicated that after sequentially removing individual studies, the pooled effect size consistently remained within the statistically significant range of −0.71 to −0.92 mmol/L (Supplementary Material S5), suggesting that the main conclusions of this study are robust and reliable, and not influenced by individual extreme data.

##### 3.4.1.3 2-h postprandial glucose

A total of 10 included studies (totaling 763 patients) reported 2hPG data. Heterogeneity test results showed moderate heterogeneity among the studies (I^2^ = 64.1%, *P* = 0.0029). Thus, a random-effects model was adopted. The meta-analysis showed that the experimental group was significantly superior to the control group in reducing 2hPG, with a pooled MD of −0.74 mmol/L (95% CI: 1.06 to −0.42, *P* = 0.0005; Figure 4C). This result suggests that CHGZGJT can effectively reduce postprandial glucose peaks in patients with T2DM. To clarify the sources of heterogeneity, meta-regression and subgroup analysis were performed. Meta-regression analysis did not show significant linear relationships between age, disease duration, treatment duration, or comorbidities and the effect size (*P* > 0.05; Supplementary Material S3). In subgroup analysis, age, disease duration, and the presence of comorbidities did not lead to obvious inter-group effect differences (*P* > 0.05; Supplementary Material S4). In the subgroup analysis of treatment duration, a trend of enhanced efficacy over time was observed. Patients with a treatment duration >60 days showed a numerically higher benefit (MD = −0.93) compared those with a duration ≤60 days (MD = −0.49; Supplementary Material S4), although this difference was not statistically significant (*P* = 0.0719). Sensitivity analysis suggested the stability of the results; after sequentially removing studies, the effect size fluctuated within a small range with a consistent direction (MD range: 0.68 to −0.81; Supplementary Material S5).

#### 3.4.2 Insulin function indicators

##### 3.4.2.1 Fasting insulin

**FIGURE 5:**
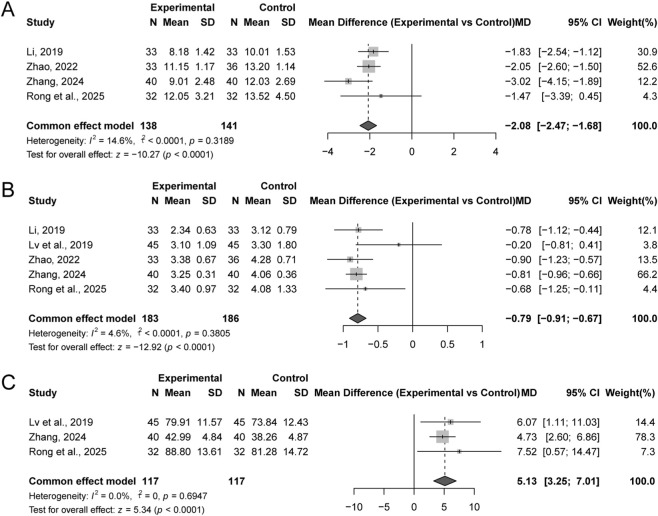
Forest plot for insulin and β-cell function-related indicators. **(A)** FINS; **(B)** HOMA-IR; **(C)** HOMA-β.

Initially, five studies were included to assess FINS levels; however, the preliminary heterogeneity test revealed significant variation among the studies (I^2^ = 87.6%, *P* < 0.0001). Through leave-one-out sensitivity analysis and a review of clinical characteristics, the study by Zhao (2023) was identified as the primary source of heterogeneity. The intervention duration in this study was only 4 weeks, significantly shorter than the 8–12 weeks in other studies. To ensure consistency in the clinical characteristics of the included studies, we excluded this short-term study from the final analysis and pooled the data from the remaining four studies (involving 279 patients). After excluding the source of heterogeneity, inter-study heterogeneity decreased significantly (I^2^ = 14.6%, *P* = 0.3189). Therefore, a fixed-effects model was adopted for the meta-analysis. The results showed that compared with the conventional therapy group, the CHGZGJT combined treatment group significantly reduced FINS levels, with a pooled MD of −2.08 µIU/mL (95% CI: 2.47 to −1.68, *P* < 0.0001; Figure 5A). This result strongly supports the clinical efficacy of the formula in ameliorating hyperinsulinemia. Sensitivity analysis demonstrated highly robust results. In the four retained long-term studies, the pooled effect size remained stable between −1.94 and −2.19 after sequentially removing any single study, and the 95% confidence intervals consistently did not cross the line of no effect (Supplementary Material S5).

##### 3.4.2.2 Insulin resistance index

Initially, a total of six studies were included. However, heterogeneity testing suggested substantial variation among studies (I^2^ = 96.1%, *P* < 0.0001). Through sensitivity analysis and a retrospective review of clinical characteristics, the study by Zhao (2023) was identified as the primary source of heterogeneity. The intervention cycle of this study was only 4 weeks, which was significantly shorter than that of other studies with intervention cycles of 8 weeks or more. Based on these considerations of clinical heterogeneity, we excluded this short-term study from the final analysis and pooled the remaining five studies (totaling 369 patients). After excluding the source of heterogeneity, inter-study heterogeneity decreased to a low level (I^2^ = 4.6%, *P* = 0.3805). Thus, a fixed-effects model was used for statistical inference. The meta-analysis results indicated that compared with the control group, the CHGZGJT combined treatment group significantly reduced the HOMA-IR index, with a pooled MD of −0.79 (95% CI: 0.91 to −0.67, *P* < 0.0001; Figure 5B). This result indicates that the formula has definitive efficacy in improving insulin sensitivity in patients with T2DM. Sensitivity analysis further verified the reliability of this conclusion. Using the leave-one-out method, the pooled effect size fluctuated within a narrow range of −0.75 to −0.81, and the statistical significance remained unchanged (Supplementary Material S5). Notably, after removing the study with the largest weight (Zhang, 2024; weight 66.2%), the effect size remained at −0.75 (95% CI: 0.96 to −0.54), indicating that the primary conclusion is not solely dependent on a single large-sample study but possesses good generalizability and robustness.

##### 3.4.2.3 Pancreatic β-cell function index

A total of three studies (involving 234 patients) assessed changes in HOMA-β before and after treatment. Heterogeneity testing showed no obvious heterogeneity among the studies (I^2^ = 0.0%, *P* = 0.6947). Therefore, a fixed-effects model was used for the pooled analysis. The meta-analysis results showed that compared with the control group, CHGZGJT combined treatment significantly increased the HOMA-β index, with a pooled MD of 5.13 (95% CI: 3.25 to 7.01, *P* < 0.0001; Figure 5C). This result corroborates the HOMA-IR analysis, suggesting that the formula not only improves peripheral IR but also significantly promotes the functional repair of damaged pancreatic β-cell secretion. Sensitivity analysis suggested the robustness of the above conclusion (Supplementary Material S5). Although the study by Zhang (2024) occupied a large weight (78.3%) in the analysis, the direction of the pooled effect size did not change after excluding this study. Instead, the MD increased to 6.56 (95% CI: 2.52–10.60), and the statistical difference remained significant. After removing any other single study, the MD value fluctuated between 4.94 and 4.97, and the results remained consistently stable. This indicates that the efficacy of the formula in improving islet function is objectively existent and not dominated by a single study.

#### 3.4.3 Lipid metabolism indicators

##### 3.4.3.1 Total cholesterol

**FIGURE 6:**
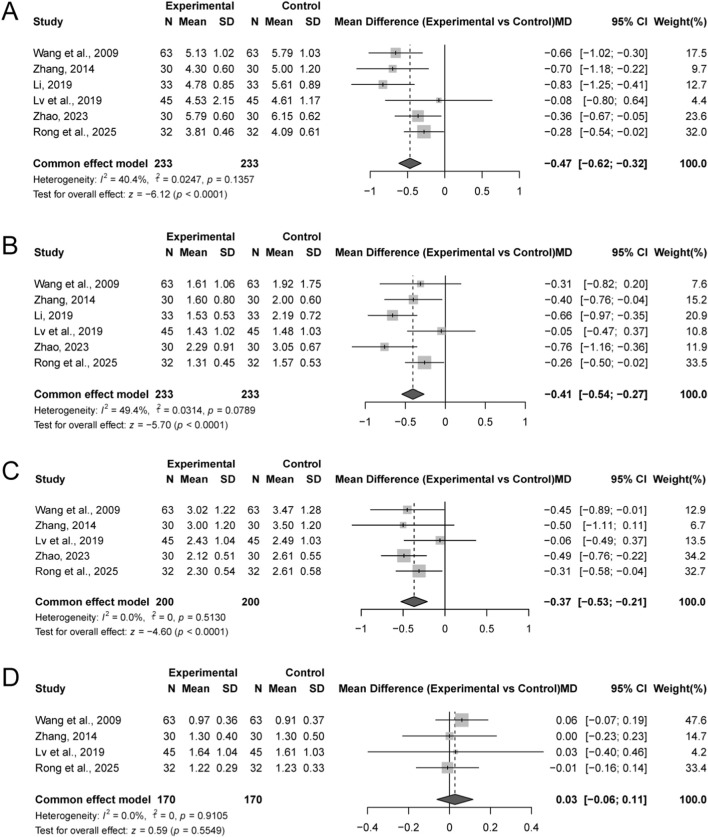
Forest plot for lipid metabolism indicators. **(A)** TC; **(B)** TG; **(C)** LDL-C; **(D)** HDL-C.

A total of six studies (involving 466 patients) reported changes in serum TC levels. Heterogeneity testing revealed no statistically significant heterogeneity among the studies (I^2^ = 40.4%, *P* = 0.1357). Therefore, a fixed-effects model was employed for the pooled analysis. The meta-analysis results indicated that CHGZGJT combined with conventional therapy significantly reduced TC levels in patients with T2DM, with a pooled MD of −0.47 mmol/L (95% CI: 0.62 to −0.32, *P* < 0.0001; Figure 6A). This suggests that the formula possesses potential clinical value in ameliorating lipid metabolism disorders while regulating glucose metabolism. Sensitivity analysis demonstrated robust and reliable results. Upon sequential removal of individual studies, the pooled effect size fluctuated between −0.41 and −0.56 mmol/L, and the 95% confidence intervals for all results did not include the line of no effect (Supplementary Material S5).

##### 3.4.3.2 Triglycerides

A total of six included studies (totaling 466 patients) provided data on serum TG levels. Heterogeneity testing suggested moderate heterogeneity among the studies (I^2^ = 49.4%, *P* = 0.0789). Based on the characteristics of the existing data, a fixed-effects model was used to estimate the pooled effect size. The meta-analysis results showed that the experimental group was significantly superior to the control group in reducing TG levels, with a pooled MD of −0.41 mmol/L (95% CI: 0.54 to −0.27, *P* < 0.0001; Figure 6B). Consistent with the findings for TC, this result indicates that CHGZGJT has definitive efficacy in improving hypertriglyceridemia in diabetic patients. Sensitivity analysis indicated that despite certain statistical heterogeneity, the main conclusion remained highly robust. After leave-one-out analysis, the pooled effect size fluctuated within the range of −0.34 to −0.48 mmol/L (Supplementary Material S5). Notably, even after removing the study with a relatively large weight (Li, 2019; weight 20.9%), the effect size decreased to −0.34 (95% CI: 0.49 to −0.18), but the difference remained highly statistically significant.

##### 3.4.3.3 Low-density lipoprotein cholesterol

A total of five studies (involving 400 patients) reported outcome data for serum LDL-C levels. Heterogeneity test results showed no obvious heterogeneity among the included studies (I^2^ = 0.0%, *P* = 0.5130). Thus, a fixed-effects model was adopted for the analysis. The meta-analysis results demonstrated that CHGZGJT combined with conventional therapy exhibited significant efficacy in reducing LDL-C, with a pooled MD of −0.37 mmol/L (95% CI: 0.53 to −0.21, *P* < 0.0001; Figure 6C). Given that LDL-C is a critical risk factor for macrovascular complications in diabetic patients, this significant reduction suggests that the formula may possess potential cardiovascular protective effects. Sensitivity analysis further suggested the reliability of this result. After sequentially removing individual studies, the pooled MD value fluctuated within a narrow range of −0.31 to −0.42 mmol/L, and all analysis results after exclusion maintained statistical significance (Supplementary Material S5). Even after removing the study with the largest weight (Zhao, 2023; weight 34.2%), the pooled effect size remained stable at −0.31 (95% CI: 0.50 to −0.11), indicating that the conclusion is robust and not dominated by a single study.

##### 3.4.3.4 High-density lipoprotein cholesterol

A total of four studies (involving 340 patients) reported HDL-C outcome indicators. Heterogeneity testing showed no apparent heterogeneity among the studies (I^2^ = 0.0%, *P* = 0.9105). Therefore, a fixed-effects model was used for the analysis. The meta-analysis results showed that although the CHGZGJT combined treatment group displayed a slight numerical trend toward increasing HDL-C compared with the control group, the difference did not reach a statistically significant level, with a pooled MD of 0.03 mmol/L (95% CI: 0.06 to 0.11, *P* = 0.5549; Figure 6D). This suggests that the lipid-regulating effect of the formula may be primarily concentrated on lowering atherogenic lipids such as TC, TG, and LDL-C, with no significant impact on HDL-C levels. Sensitivity analysis results were consistent with the primary analysis. After sequentially removing individual studies, the pooled effect size consistently fluctuated between 0.00 and 0.04 mmol/L, and all 95% confidence intervals crossed the line of no effect (Supplementary Material S5). This confirms the robustness of the conclusion that the formula has no significant effect on improving HDL-C.

### 3.5 Secondary outcome measures

#### 3.5.1 C-reactive protein

**FIGURE 7:**
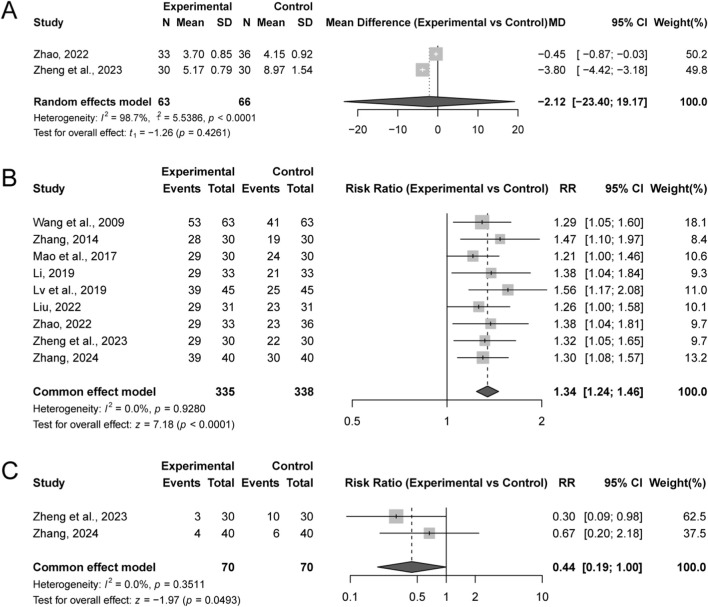
Forest plot for secondary outcomes. **(A)** C-reactive protein; **(B)** Overall effectiveness; **(C)** Adverse events.

A total of two studies (involving 129 patients) assessed changes in serum CRP levels before and after treatment to evaluate the chronic low-grade inflammatory state. Due to the substantial disparity in effect size values between the two included studies (MD of −0.45 and −3.80, respectively), statistical heterogeneity was extremely high (I^2^ = 98.7%, *P* < 0.0001). Constrained by the limited sample size and high heterogeneity, the results pooled using a random-effects model showed no statistically significant difference between the combined treatment group and the control group in reducing CRP, with a pooled MD of −2.12 mg/L (95% CI: 23.40 to 19.17, *P* = 0.4261; Figure 7A). However, an in-depth analysis of individual studies revealed that both trials reported a decreasing trend in CRP levels following combined treatment with CHGZGJT. Sensitivity analysis indicated that regardless of which study was retained, the single-study effect size suggested superiority of the experimental group over the control group (MD values were all less than 0, and confidence intervals did not cross the line of no effect; Supplementary Material S5). This suggests that although current data preclude a definitive quantitative conclusion at the meta-analysis level due to excessive heterogeneity, the formula may possess potential clinical benefits in ameliorating the chronic inflammatory response in patients with T2DM, which warrants further verification through more homogeneous, high-quality studies.

#### 3.5.2 Overall effective rate

A total of nine studies (involving 673 patients) reported the overall effective rate. Heterogeneity test results showed no obvious heterogeneity among the studies (I^2^ = 0.0%, *P* = 0.9280), indicating high consistency in efficacy adjudication across different studies. Therefore, a fixed-effects model was used for data pooling. The meta-analysis results showed that the overall effective rate in the CHGZGJT combined with conventional therapy group was significantly higher than that in the conventional therapy alone group, with a pooled RR of 1.34 (95% CI: 1.24 to 1.46, *P* < 0.0001; Figure 7B). This result suggests that the combined treatment regimen can significantly enhance the clinical response rate and improve comprehensive treatment outcomes in patients with T2DM. Sensitivity analysis further suggested the high robustness of this conclusion. Re-analysis using the leave-one-out method revealed that the pooled RR consistently remained within a narrow range of 1.32–1.36, and the lower limits of all 95% confidence intervals were greater than 1.0 (Supplementary Material S5). This indicates that the conclusion regarding the formula’s ability to improve the clinical effective rate is relatively reliable and not overly influenced by any single study.

#### 3.5.3 Adverse events rate

Among the 12 included studies, six reported safety-related information. Of these, four studies (Wang and Xiao, 2009; Zhang, 2014; Mao and Wang, 2017; Zhao, 2022) explicitly stated that no apparent adverse events had occurred, and no clinically significant abnormalities were observed in complete blood count, urinalysis, hepatic and renal function, or electrocardiography before and after treatment. Two studies (Zheng and Ma, 2023; Zhang, 2024) reported quantifiable incidences of adverse events, and the events recorded were confined to mild, self-limiting reactions, including gastrointestinal symptoms (nausea, abdominal distension, and constipation), skin rash, dry mouth, somnolence, insomnia, and fatigue. No study reported any serious adverse events, hospitalization, or treatment discontinuation. A study-level summary is provided in Supplementary Material S6. Analysis of the two aforementioned trials revealed no apparent heterogeneity (I^2^ = 0.0%, *P* = 0.3511), and a fixed-effects model was therefore applied. The results indicated that, compared with conventional biomedical pharmacotherapy alone, the group receiving combined CHGZGJT treatment exhibited a significantly reduced risk of adverse events, with a pooled RR of 0.44 (95% CI: 0.19 to 1.00, *P* = 0.0493; Figure 7C). This finding suggests that the TCM formula may possess a favorable safety profile and, when administered in combination, may help to alleviate the adverse reactions induced by conventional hypoglycemic agents. Sensitivity analysis indicated that the robustness of the results was somewhat limited by the small number of included studies (Supplementary Material S5). When the study with a larger sample weight (Zheng and Ma, 2023) was excluded, although the estimated pooled effect size still favored the experimental group (RR = 0.67), the difference was no longer statistically significant (95% CI: 0.20–2.18). Therefore, although the current pooled evidence supports the safety advantage of this formula, further large-sample, high-quality RCTs are still warranted to confirm its precise value in reducing drug-related adverse reactions.

### 3.6 Restricted analysis and quality of evidence assessment

To further verify the robustness of the results, a restricted analysis was conducted in which the meta-analysis was repeated using data from only the 10 RCTs. The analysis demonstrated that none of the pooled estimates changed substantially, and the direction and statistical significance of each outcome measure remained unchanged (Supplementary Material S7). In addition, the quality of evidence for the outcome measures was assessed using the Grading of Recommendations Assessment, Development and Evaluation (GRADE) approach (Supplementary Material S8). FINS, HOMA-IR, TC, TG, and LDL-C were rated as moderate quality; HbA1c, FPG, 2hPG, HOMA-β, HDL-C, the overall response rate, and adverse events were rated as low quality; and CRP was rated as very low quality, primarily attributable to risk of bias and, for certain glycemic outcomes, unexplained heterogeneity. The aforementioned ratings indicate that, although the observed direction of effects was consistent, the findings should nonetheless be interpreted with appropriate caution.

### 3.7 Publication bias assessment

**FIGURE 8:**
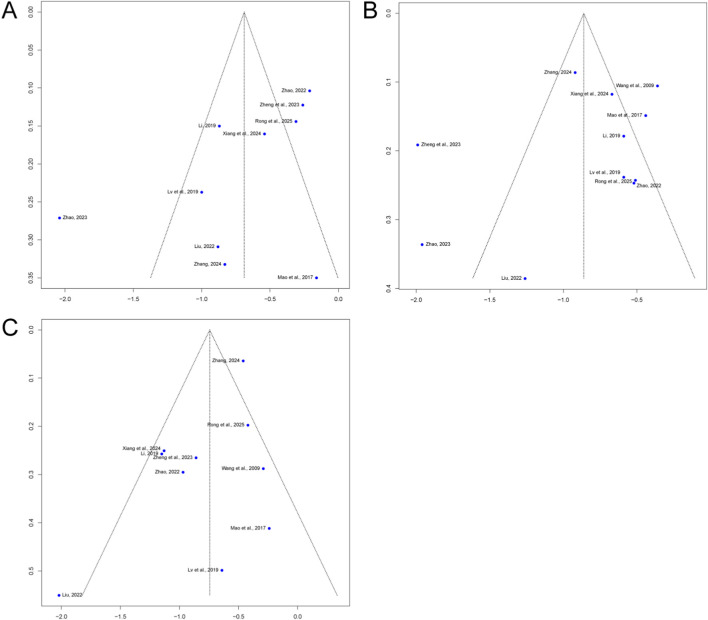
Funnel plots for assessing publication bias. **(A)** HbA1c; **(B)** FPG; **(C)** 2hPG.

Publication bias was assessed for the three outcomes of HbA1c, FPG, and 2hPG which included 10 or more studies. The assessment strategy involved qualitative visual inspection of funnel plots (Figure 8) combined with quantitative verification using Egger’s linear regression test. For HbA1c, the funnel plot displayed a generally symmetrical distribution of studies around the pooled effect size. Although some scatter points deviated from the midline, no distinct asymmetry or missing corners were observed. Egger’s test further corroborated the visual inspection, revealing no statistically significant asymmetry (t = −2.17, *P* = 0.0622; Supplementary Material S9A), indicating the absence of significant publication bias for this indicator. Regarding the analysis of FPG, the funnel plot exhibited good symmetry, with the majority of studies concentrated within the confidence interval at the apex of the funnel. Egger’s test (t = −0.83, *P* = 0.4269; Supplementary Material S9B) detected no significant publication bias. For 2hPG, although visual inspection of the funnel plot suggested a slight trend toward left-sided distribution, Egger’s test (t = −2.08, *P* = 0.0707; Supplementary Material S9C) did not reveal statistically significant asymmetry. This suggests that while there may be potential signs of small-study effects, they were insufficient to constitute statistically significant publication bias. Consistent results from Egger’s tests across key outcome indicators suggest that the findings included in this meta-analysis possess good reliability, with no significant publication bias detected, indicating that the results are relatively robust.

## 4 Discussion

### 4.1 Main results of this study

This study systematically constructed and quantified the evidence base for the efficacy and safety of CHGZGJT combined with conventional biomedical treatment in patients with T2DM through a meta-analysis. The results demonstrated that the CHGZGJT combination regimen yielded clear clinical benefits across multiple dimensions. Regarding efficacy, the combination therapy significantly improved glycemic control in T2DM patients, as evidenced by comprehensive reductions in HbA1c (the gold standard reflecting long-term glycemic levels), FPG, and 2hPG. Concurrently, the treatment significantly ameliorated the atherogenic lipid profile, lowering TC, TG, and LDL-C levels. More importantly, the study revealed the positive effects of this formula on the core pathophysiological processes of T2DM, effectively reducing FINS levels and HOMA-IR while increasing HOMA-β. This demonstrates a dual potential to enhance insulin sensitivity and protect islet cell function. These biochemical improvements ultimately translated into a higher overall clinical efficacy rate. In terms of safety, preliminary pooled analysis suggested that the combination therapy might help reduce the overall risk of adverse events, indicating favorable tolerability. Through this systematic meta-analysis, the study constructed a complete chain of evidence from efficacy validation to safety evaluation, laying a solid foundation for a deeper exploration of the clinical value and mechanisms of action of CHGZGJT in treating T2DM.

### 4.2 Clinical and mechanistic interpretation of efficacy

#### 4.2.1 Significance and potential mechanisms of glucose metabolism improvement

This study confirms that CHGZGJT combined with conventional therapy significantly ameliorates the glucose metabolic status of patients with T2DM, evidenced by comprehensive reductions in HbA1c, FPG, and 2hPG. As the international gold standard reflecting average blood glucose levels over the preceding 8–12 weeks, the reduction of HbA1c is explicitly correlated with a decreased risk of diabetic microvascular and macrovascular complications (ElSayed et al., 2024b). Although the observed mean reduction in HbA1c of 0.69% in this study did not reach the magnitude of reduction seen with certain intensive therapies, it possesses definitive clinical value. Epidemiological and clinical trial data indicate that every 1% reduction in HbA1c significantly lowers the risk of diabetes-related endpoints, microvascular complications, and myocardial infarction (ElSayed et al., 2024b; Zhu and Guo, 2025). Therefore, the HbA1c reduction achieved by the CHGZGJT combined regimen has positive practical significance for delaying or preventing chronic complications such as diabetic nephropathy and retinopathy, thereby favoring the improvement of long-term patient prognosis.

The results of the subgroup analysis provide critical clues for the precise targeting of patient populations. We found that patients who were younger (<55 years) and without comorbidities exhibited a more significant reduction in HbA1c. This phenomenon carries dual clinical implications. On one hand, it aligns with the TCM philosophy of “preventive treatment of disease” (*Zhi Wei Bing*) and early intervention. In relatively young patients with a shorter disease duration and less complex conditions, the TCM pathogenesis is predominantly characterized by liver depression and spleen deficiency, while complex concurrent syndromes such as phlegm-stasis intermingling are not yet profound. CHGZGJT excels in soothing the liver to resolve depression, clearing heat to generate fluids, and warming yang to transform rheum, which directly addresses the core pathogenesis of this stage, thus yielding optimal efficacy. On the other hand, this corresponds highly with the emphasis in modern medicine on active intervention in early-stage T2DM. Research indicates that intensive glycemic control in the early stages of the disease not only effectively lowers the risk of complications but also confers long-term clinical benefits through the “metabolic memory” effect (ElSayed et al., 2024b; Zhu and Guo, 2025). Consequently, this formula may be particularly suitable for the patient population in the early-to-mid stages of T2DM, characterized predominantly by IR with partially preserved β-cell compensatory capacity.

Regarding potential mechanisms of action, insights can be drawn from both the holistic view of TCM and modern pathophysiology. First, TCM theory categorizes T2DM under the domain of wasting and thirsting, attributing its onset and progression to the dysfunction of the liver, spleen, and kidney. Emotional distress and dietary irregularity can lead to liver qi stagnation, which transversely invades the spleen. The spleen then fails to transport and transform, causing the non-distribution of water and grain essence, which brews into phlegm-rheum and stagnates into heat, consuming qi and yin. In CHGZGJT, *Bupleuri Radix* (Chaihu) and *Scutellariae Radix* (Huangqin) soothe the liver and clear heat to resolve Shaoyang depression; *Cinnamomi Ramulus* (Guizhi) and *Zingiberis Rhizoma* (Ganjiang) warm and unblock middle yang to aid the spleen in transforming fluid; *Ostreae Concha* (Muli) and *Trichosanthis Radix (Tianhuafen)* soften hardness to dissipate binding and generate fluids to astringe yin; and *Glycyrrhizae Radix et Rhizoma* (Gancao) harmonizes the botanical drugs. The formula combines cold and heat properties and employs both attacking and reinforcing methods, collectively achieving the regulation of qi mechanism, restoration of middle jiao transport, and balancing of yin and yang, thereby enabling the distribution of fluids and the clearing of dryness and heat, which systematically ameliorates the disordered state of glucose metabolism. Second, based on modern pharmacology and the results of this study, it is hypothesized that the formula may exert its effects by improving IR. The significant reduction in HOMA-IR observed in this study suggests that enhancing insulin sensitivity in peripheral tissues (particularly the liver and skeletal muscle) is one of its primary pathways. IR is the core pathological basis of T2DM, associated with multiple mechanisms including ectopic lipid deposition, chronic inflammation, and endoplasmic reticulum stress (Lee et al., 2022). The multiple metabolites of CHGZGJT likely act through multi-target pathways. For instance, its lipid-regulating effects may help reduce lipid accumulation in the liver and muscle, thereby alleviating lipotoxicity-induced IR. Furthermore, the potential downregulation trend of CRP by this formula suggests it may possess certain anti-inflammatory properties, and chronic low-grade inflammation is a key factor driving IR (Dludla et al., 2023; Pellegrini et al., 2024). By inhibiting inflammatory pathways, it may indirectly improve insulin signal transduction. Regarding the protection of pancreatic β-cell function, the significant elevation of the HOMA-β index is another critical finding of this study. β-cell dysfunction is closely related to glucolipotoxicity, oxidative stress, and inflammatory infiltration (Dludla et al., 2023; Park et al., 2025). The metabolites of the heat-clearing and yang-warming botanical drugs of CHGZGJT may contain antioxidant and anti-inflammatory activities. For example, metabolites such as baicalin have been widely studied for their effects against inflammation and oxidative stress. By mitigating oxidative damage and inflammatory responses in a high-glucose and high-fat environment, this formula may protect β-cells from apoptosis and improve their insulin secretion function. This aligns with the TCM concept of strengthening the body’s defenses and expelling pathogens to regulate organ function. Regarding the regulation of postprandial glucose fluctuations, the significant reduction in 2hPG indicates that this formula helps blunt postprandial glucose peaks. Postprandial hyperglycemia is not only a major source of overall glucose variability but also a cardiovascular risk factor independent of HbA1c (Takao et al., 2017). Its mechanism may involve delaying carbohydrate absorption in the gastrointestinal tract, improving first-phase insulin secretion, or inhibiting inappropriate postprandial glucagon release. TCM posits that postprandial glucose elevation is directly related to “spleen deficiency and transport dysfunction”. The formula’s efficacy in warming and transporting spleen yang and fortifying the spleen to transform fluid may constitute the mechanism underlying its improvement of postprandial glucose. The comprehensive improvement of glucose metabolism in T2DM patients by CHGZGJT is a concrete manifestation of its TCM therapeutic principles of “soothing the liver and resolving depression, clearing heat and generating fluids, and warming yang to transform rheum” in modern disease management. Its potential mechanisms likely involve synergistic actions across multiple targets and pathways, covering the improvement of insulin sensitivity, protection of β-cell function, regulation of lipid metabolism, and potential alleviation of chronic inflammation, thereby achieving systemic regulation of glucose homeostasis.

#### 4.2.2 Deep impact on insulin resistance and β-cell function

The synergistic amelioration of FINS, HOMA-IR, and HOMA-β observed in this study addresses the most pivotal pathophysiological mechanisms of T2DM and provides critical evidence for the depth of the therapeutic action of CHGZGJT. The meta-analysis results demonstrate that combined treatment with CHGZGJT not only reduced FINS levels, which reflects an alleviation of hyperinsulinemia, but also synchronously and significantly improved peripheral insulin sensitivity and augmented β-cell function. This triad of effects involving the reduction of metabolic load, the enhancement of sensitivity, and the repair of function constitutes an ideal closed loop for metabolic improvement. This comprehensive action stands in sharp contrast to many biomedical drugs that operate *via* single mechanisms. For instance, traditional sulfonylureas primarily stimulate insulin secretion, which may exacerbate β-cell exhaustion and fails to address insulin resistance (Zhu and Guo, 2025). Conversely, while thiazolidinediones are potent insulin sensitizers, evidence regarding their direct enhancement of β-cell function remains inconsistent, and they are associated with risks such as fluid retention (Lee et al., 2022). The multi-target synergistic action exhibited by CHGZGJT embodies the holistic TCM concept of systematically reconstructing the body’s metabolic homeostasis by harmonizing the liver and spleen. Its objective extends beyond merely lowering blood glucose to correcting the fundamental pathological state driving hyperglycemia, specifically the coexistence of defective insulin action and insufficient secretion.

This early synergistic intervention targeting IR and β-cell function holds profound implications for clinical prognosis. IR is not merely a driver of hyperglycemia but also a common risk factor linking obesity, dyslipidemia, hypertension, and cardiovascular disease (Lee et al., 2022; Marx et al., 2023). Prospective cohort studies have suggested that even in normoglycemic populations, elevated HOMA-IR is an independent risk factor for the future development of T2DM and chronic kidney disease (Lee et al., 2023). Therefore, effectively ameliorating IR in the early stages of the disease is tantamount to intervening upstream in the cascade of metabolic derangement, potentially delaying or preventing the progression to complications. Furthermore, protecting and enhancing β-cell function is the cornerstone of maintaining long-term glycemic stability. Research suggests that intensive therapy aimed at improving β-cell function in the early stages of diabetes is associated with higher rates of diabetes remission (Zhu and Guo, 2025). The elevation in HOMA-β observed in this study, combined with a favorable safety profile, suggests that CHGZGJT as part of a combination regimen may secure a prolonged honeymoon period for patients. This could reduce the need for reliance on more potent or numerous hypoglycemic agents and thereby improve long-term therapeutic outcomes and quality of life. The deep amelioration of IR and β-cell function represents a key mechanism underlying the therapeutic efficacy of CHGZGJT in T2DM. Applying a formula with such comprehensive regulatory capabilities in the early stages of the disease is of significant strategic value for breaking the vicious cycle of IR and β-cell failure and modifying the natural course of T2DM.

#### 4.2.3 Lipid metabolism regulation and potential cardiovascular benefits

This study demonstrates that CHGZGJT exerts a definitive regulatory effect on the lipid metabolism disorders accompanying T2DM while simultaneously improving glucose metabolism. The meta-analysis results indicate that the combined treatment significantly reduced serum TC, TG, and LDL-C levels but had no significant effect on HDL-C. This unique pattern of lipid profile improvement possesses significant clinical pathophysiological implications.

First, the specific lipid abnormalities ameliorated by CHGZGJT correspond precisely to the atherogenic dyslipidemia characteristic of T2DM. This dyslipidemia is characterized chiefly by elevated fasting and postprandial TC, reduced HDL-C, and an increase in small dense LDL-C particles. Its core driving mechanism involves increased lipolysis in adipose tissue, excessive hepatic synthesis of very low-density lipoprotein, and impaired lipoprotein clearance resulting from IR (Marx et al., 2023; Gaita et al., 2024). The synchronous reduction in TG and TC observed in this study suggests that the formula may reduce the influx of free fatty acids into the liver and the excessive synthesis of lipoproteins by improving IR upstream. Of particular critical importance is the significant reduction in LDL-C. Current authoritative international guidelines consistently identify LDL-C as the primary target for lipid-lowering therapy in atherosclerotic cardiovascular disease (ASCVD) and establish strict LDL-C control targets, such as < 1.8 mmol/L or <1.4 mmol/L, for high-risk or very-high-risk populations like those with T2DM (Marx et al., 2023; Gaita et al., 2024; Sousa et al., 2025). Although the mean reduction in LDL-C achieved by CHGZGJT combined therapy did not reach the efficacy of high-intensity statins, it represents an additional benefit on top of conventional therapy. This aids more patients in approaching or achieving lipid control targets and is directly associated with a potential reduction in cardiovascular event risk. Second, the lack of significant elevation in HDL-C requires objective interpretation. In the prevention and treatment of atherosclerosis, reducing atherogenic lipoproteins such as LDL-C has been suggested as a causal intervention strategy, whereas pharmacologically raising HDL-C has not consistently translated into cardiovascular benefits (Gaita et al., 2024). Therefore, the efficacy of CHGZGJT in lowering TC, TG, and LDL-C aligns with current priority strategies for lipid management. This suggests that its mechanism of lipid regulation may differ from that of fibrates or novel agents and is more likely realized through a benign restructuring of the lipoprotein profile achieved by globally improving the metabolic state.

CHGZGJT demonstrated the potential to synchronously ameliorate multiple cardiovascular risk factors in patients with T2DM. Cardiovascular risk management in T2DM is a comprehensive endeavor, and its success depends on the degree of control over multiple risk factors including blood glucose, blood pressure, blood lipids, and obesity. A nationwide cohort study in Sweden suggested that when five key risk factors including HbA1c, blood pressure, LDL-C, smoking, and albuminuria are all within target ranges, the risks of all-cause mortality, myocardial infarction, and stroke in patients with T2DM can be reduced to levels comparable to those of the general population without diabetes (Rawshani et al., 2018). This study reveals that the intervention of CHGZGJT combined with conventional therapy confers additional multiple improvements in blood glucose, insulin sensitivity, and the atherogenic lipid profile. This multi-target effect pattern is highly valuable as it implies that for T2DM patients with dyslipidemia, combining CHGZGJT with existing standard treatment regimens may simultaneously optimize multiple risk domains and thereby more efficiently reduce the residual cardiovascular risk of patients (Pellegrini et al., 2024; Sousa et al., 2025). From the perspective of TCM theory, dyslipidemia is generally categorized under the domains of fluid retention, phlegm turbidity, and blood stasis. The root causes lie in the loss of liver dredging and spleen transport, leading to the failure of transformation of water and grain essence and the accumulation of dampness to generate phlegm. In CHGZGJT, *Bupleuri Radix* (Chaihu) and *Scutellariae Radix* (Huangqin) soothe the liver and clear heat to resolve qi stagnation, while *Cinnamomi Ramulus* (Guizhi) and *Zingiberis Rhizoma* (Ganjiang) warm yang and transform qi to aid spleen and stomach transport. These actions address the core pathogenesis of phlegm turbidity generation from the level of simultaneous liver and spleen regulation. Consequently, its lipid-regulating action can be viewed as a direct manifestation of the therapeutic methods of soothing the liver, fortifying the spleen, and warming yang to transform rheum at the micro-metabolic level. The regulation of lipid metabolism by CHGZGJT, particularly the reduction of LDL-C, constitutes a significant component of its clinical benefit. By synergistically improving blood glucose, IR, and the atherogenic lipid profile, this formula may provide an integrated Chinese and biomedical treatment strategy that facilitates the comprehensive management of cardiovascular risk and the reduction of residual risk in patients with T2DM.

#### 4.2.4 Inflammatory state and safety analysis

In addition to glucose and lipid metabolism and insulin function, this study provides preliminary evidence for assessing the impact of CHGZGJT on the chronic low-grade inflammatory state of T2DM and its overall safety. The meta-analysis targeting serum CRP levels failed to yield a statistically significant pooled effect size because it included only two studies and exhibited extremely high heterogeneity. However, an in-depth examination of the individual studies revealed that both trials reported a decreasing trend in CRP levels following combined treatment with CHGZGJT. This signal possesses important pathophysiological implications as substantial evidence indicates that chronic low-grade inflammation serves as a core pathological link connecting obesity, IR, T2DM, and cardiovascular complications (Dludla et al., 2023; Pellegrini et al., 2024). Inflammatory factors such as CRP and IL-6 can interfere with insulin signal transduction to exacerbate IR and directly damage vascular endothelial function, which promotes the formation and progression of atherosclerotic plaques (Marx et al., 2023; Atak Tel et al., 2025). Therefore, even though the effect of CHGZGJT on reducing CRP remains inconclusive in this pooled analysis, the observed decreasing trend warrants high attention. This suggests that the heat-clearing efficacy of the formula may be realized partially through the regulation of inflammatory pathways, which complements its metabolic improvement effects. Future research must include more high-quality randomized controlled trials with homogeneous designs and standardized measurements of inflammatory markers to clarify whether CHGZGJT possesses definitive anti-inflammatory effects and to explore the association between these effects and improvements in clinical endpoints.

Current evidence indicates that, when used as an adjunct to conventional treatment, CHGZGJT possesses a favorable short-term safety profile over treatment courses of up to 12 weeks. The reported adverse events were low in incidence, mild in severity, and self-limiting, and routine hematological, hepatic, renal, and electrocardiographic monitoring revealed no clinically significant abnormalities. The number of adverse events in the combination therapy group was lower than that in the control group, which is consistent with the traditional view that this formula exerts gastrointestinal-protective and harmonizing effects. Nevertheless, this signal was derived from only two trials and should be interpreted with caution. In addition, several pharmacological issues warrant attention, in particular the fact that none of the included trials systematically evaluated interactions between botanical drugs and conventional medicines or documented concomitant medication use in detail. The formula contains *Glycyrrhizae Radix et Rhizoma* (Gancao), whose principal metabolite, glycyrrhizic acid, may induce pseudoaldosteronism (manifesting as hypokalemia, sodium and water retention, and hypertension) when used over prolonged periods or at high doses, and may interact with potassium-depleting diuretics, glucocorticoids, and digitalis glycosides (Nazari et al., 2017; Deutch et al., 2019). It is therefore prudent to limit the dosage and duration of licorice-containing preparations and to monitor blood pressure and serum potassium during long-term use (Omar et al., 2012). As the formula itself exerts hypoglycemic effects, its combination with insulin or oral hypoglycemic agents may produce additive effects; accordingly, monitoring for hypoglycemia is recommended, with adjustment of the dosage of conventional hypoglycemic agents where necessary. Future trials should prospectively document concomitant medication use, specifically monitor hypoglycemic events, evaluate pharmacokinetic and pharmacodynamic interactions, adopt a standardized adverse event grading system such as the Common Terminology Criteria for Adverse Events (CTCAE), and incorporate longer follow-up periods to clarify the long-term safety of this preparation.

### 4.3 Research limitations and future perspectives

While elucidating the evidential value of this study, its limitations must be objectively examined and a clear roadmap for future research outlined on the basis of the existing findings. First, the methodological quality of the included primary studies represents a weak link in the chain of evidence. The body of evidence in this study comprised two non-randomized controlled studies. Although these were each evaluated using the ROBINS-I tool (both being rated as having a moderate risk of bias) and prespecified restricted analysis suggested that all conclusions remained unchanged after the exclusion of these two studies, this nonetheless constitutes one of the limitations of the present work. Moreover, most RCTs did not adequately report allocation concealment or the implementation of blinding, nor were they prospectively registered, and consequently the quality of evidence as assessed by GRADE was generally low. On this basis, the current findings should be regarded as supportive evidence rather than confirmatory conclusions. Second, the substantial clinical heterogeneity could not be fully explained. At the intervention level, CHGZGJT was applied across the studies in the form of modified formulas, and its variations in the dosages of individual medicinals, the composition of added and subtracted medicinals, the geographical origin of the medicinal materials, and the decoction process constituted sources of residual heterogeneity that were difficult to quantify; moreover, owing to the differences in formula composition across studies, a formal dose-response analysis could not be conducted at present. The ConPhYMP-based assessment indicated that none of the original trials performed chemical characterization or standardization of their decoctions, and information regarding the source, identification, processing, and batch-to-batch consistency of the crude drugs was largely absent. This lack of pharmaceutical characterization limited the chemical comparability of the pooled interventions and the reproducibility of the evidence. At the level of background treatment, the biomedical hypoglycemic regimens administered to the control groups varied across the included studies, encompassing different regimens and dosages of metformin, sulfonylureas, SGLT-2 inhibitors, and insulin. Although the existing subgroup and sensitivity analysis supported the robustness of the conclusions, the potential influence of background treatment heterogeneity on the pooled effect measures remains to be quantitatively elucidated in larger-sample studies. Third, the treatment duration of the included studies was an important constraint on the reliability of the HbA1c assessment. HbA1c reflects a weighted average of blood glucose levels over the preceding approximately 2–3 months, and the establishment of its complete steady state requires approximately 90–120 days, whereas more than half of the studies included in this work had treatment courses of less than 90 days. This implies that the changes in HbA1c observed in some studies may not have fully reflected the true long-term effects of treatment, posing a risk of underestimating the effect size. As the existing subgroup analysis (stratified by treatment duration) was statistically underpowered owing to the limited number of studies, the quantitative influence of treatment duration on the HbA1c effect could not be adequately elucidated. This further underscores the necessity of conducting long-term follow-up studies. Fourth, with respect to safety, only half of the included studies reported adverse events, and only two studies provided quantifiable event data. The ascertainment and reporting of adverse events did not follow standardized methods, and no study systematically evaluated interactions between botanical drugs and conventional medicines or long-term safety. Therefore, the current conclusions regarding safety are merely preliminary.

The aforementioned limitations point to clear directions for future research. The foremost priority is to conduct prospective, large-sample, long-duration, high-quality RCTs in order to overcome the current deficiency in methodological quality. Such studies should adhere to the Consolidated Standards of Reporting Trials (CONSORT) statement, adopt a multicenter, randomized, double-blind, placebo-controlled design, and undergo clinical trial registration in advance so as to minimize selection and measurement bias. To address the clinical heterogeneity that could not be fully explained in this study, botanical drug interventions should, at the intervention level, be reported in accordance with the GA/ConPhYMP best-practice standards, including the provision of information on taxonomically authenticated crude drug sources, processing details, decoction procedures, and chemical fingerprints, thereby achieving the standardization and quality control of CHGZGJT preparations. At the level of background treatment, future studies are recommended to standardize the basic biomedical hypoglycemic regimens as far as possible, or to evaluate the differential effects of CHGZGJT combined with distinct classes of hypoglycemic agents through large-sample stratified designs, in order to quantify the influence of background treatment heterogeneity on the pooled effect measures. With regard to the reliability of efficacy evaluation, the treatment duration of future RCTs should be no less than 3 months (with 6 months or longer recommended), and HbA1c should be assessed at standardized time points (such as baseline and 3 and 6 months of treatment) to ensure that this indicator adequately reflects the true impact of the therapeutic intervention on long-term glycemic control. In addition, to address the insufficiency of safety evidence, future studies should systematically collect and report all adverse events using a standardized adverse event grading system such as CTCAE, prospectively document concomitant medication use, specifically monitor hypoglycemic events and interactions between botanical drugs and conventional medicines, and extend the follow-up period to evaluate long-term safety.

## 5 Conclusion

This study demonstrates that CHGZGJT combined with conventional treatment offers advantages over conventional treatment alone in the management of T2DM. This combination regimen shows some potential in alleviating insulin resistance (HOMA-IR), enhancing pancreatic β-cell function (HOMA-β), and further improving glycemic control (HbA1c, FPG, 2hPG) while regulating lipid metabolism (TC, TG, LDL-C). Moreover, CHGZGJT was generally well tolerated and may contribute to a reduced incidence of adverse events associated with conventional therapy. However, given the variable methodological quality of the included studies, the relatively limited sample sizes, and the potential for heterogeneity, the strength of the evidence from this study requires further reinforcement, and the results should be interpreted and applied with caution. In the clinical management of T2DM, treatment decisions should still integrate a comprehensive assessment of the patient’s overall condition and the evolution of their TCM syndrome patterns. Future rigorously designed, large-scale, multicenter, randomized, double-blind, placebo-controlled trials, alongside in-depth mechanistic studies at the molecular level, are urgently needed to provide robust evidence regarding the long-term benefits and safety of CHGZGJT in T2DM treatment, thereby offering new directions for clinical practice.

## Data Availability

The original contributions presented in the study are included in the article/Supplementary Material, further inquiries can be directed to the corresponding author.
