# Supplementary material for: The efficacy and safety of Chaihu guizhi ganjiang tang for type 2 diabetes mellitus: a systematic review and meta-analysis
Source: Front Pharmacol. 2026 Jun 25;17:1855616. doi: 10.3389/fphar.2026.1855616 (PMC13345826; doi:10.3389/fphar.2026.1855616)
Supplement: Supplementary file 1 [file Supplementaryfile1.docx]

# Appendix 1

## Botanical, zoological and pharmaceutical nomenclature of the constituent drugs of Chaihu Guizhi Ganjiang Tang reported in the included studies

The scientific name, naming authority and family of every constituent drug were validated against the Medicinal Plant Names Services (MPNS, Royal Botanic Gardens, Kew) and Plants of the World Online (POWO). Drugs of animal (†) and fungal (‡) origin, which are not covered by MPNS/POWO, were verified against the World Register of Marine Species (WoRMS) / fauna references and Index Fungorum, respectively. Pharmaceutical (drug) names and medicinal parts follow the Pharmacopoeia of the People's Republic of China (2020 edition).

| **No.** | **Common name (Pinyin)** | **Validated scientific name (with naming authority)** | **Family** | **Pharmaceutical (drug) name** | **Medicinal part** |
| --- | --- | --- | --- | --- | --- |
| ***Principal drugs of the classical Chaihu Guizhi Ganjiang Tang (Shanghan Lun)*** | | | | | |
| 1 | Chaihu | *Bupleurum chinense* DC. (also *B. scorzonerifolium* Willd.) | Apiaceae | *Bupleuri Radix* | Root |
| 2 | Guizhi | *Cinnamomum cassia* (L.) J.Presl (syn. *C. aromaticum* Nees) | Lauraceae | *Cinnamomi Ramulus* | Twig (young branch) |
| 3 | Ganjiang | *Zingiber officinale* Roscoe | Zingiberaceae | *Zingiberis Rhizoma* | Dried rhizome |
| 4 | Huangqin | *Scutellaria baicalensis* Georgi | Lamiaceae | *Scutellariae Radix* | Root |
| 5 | Tianhuafen | *Trichosanthes kirilowii* Maxim. | Cucurbitaceae | *Trichosanthis Radix* | Root |
| 6 | Muli † | *Magallana gigas* (Thunberg, 1793) (syn. *Crassostrea gigas*; *Ostrea gigas* Thunb.) | Ostreidae (Animalia) | *Ostreae Concha* | Shell |
| 7 | Gancao / Zhigancao | *Glycyrrhiza uralensis* Fisch. ex DC. (also *G. inflata* Bat.; *G. glabra* L.) | Fabaceae | *Glycyrrhizae Radix et Rhizoma* | Root and rhizome |
| ***Additional drugs used in the individual modified formulae (alphabetical by Pinyin)*** | | | | | |
| 8 | Baishao | *Paeonia lactiflora* Pall. | Paeoniaceae | *Paeoniae Radix Alba* | Root |
| 9 | Baixianpi | *Dictamnus dasycarpus* Turcz. | Rutaceae | *Dictamni Cortex* | Root bark |
| 10 | Baizhu | *Atractylodes macrocephala* Koidz. | Asteraceae | *Atractylodis Macrocephalae Rhizoma* | Rhizome |
| 11 | Cangzhu | *Atractylodes lancea* (Thunb.) DC. (also *A. chinensis* (DC.) Koidz.) | Asteraceae | *Atractylodis Rhizoma* | Rhizome |
| 12 | Chenpi | *Citrus reticulata* Blanco | Rutaceae | *Citri Reticulatae Pericarpium* | Pericarp |
| 13 | Chishao | *Paeonia lactiflora* Pall. (also *P. veitchii* Lynch) | Paeoniaceae | *Paeoniae Radix Rubra* | Root |
| 14 | Chuanlianzi | *Melia toosendan* Siebold & Zucc. | Meliaceae | *Toosendan Fructus* | Fruit |
| 15 | Chuanxiong | *Ligusticum striatum* DC. (syn. *L. chuanxiong* Hort.) | Apiaceae | *Chuanxiong Rhizoma* | Rhizome |
| 16 | Dangshen | *Codonopsis pilosula* (Franch.) Nannf. | Campanulaceae | *Codonopsis Radix* | Root |
| 17 | Mudanpi | *Paeonia* × *suffruticosa* Andrews | Paeoniaceae | *Moutan Cortex* | Root bark |
| 18 | Danshen | *Salvia miltiorrhiza* Bunge | Lamiaceae | *Salviae Miltiorrhizae Radix et Rhizoma* | Root and rhizome |
| 19 | Dilong † | *Pheretima aspergillum* (E. Perrier, 1872) | Megascolecidae (Animalia) | *Pheretima* | Dried body |
| 20 | Fabanxia | *Pinellia ternata* (Thunb.) Makino | Araceae | *Pinelliae Rhizoma Praeparatum* | Tuber |
| 21 | Fangfeng | *Saposhnikovia divaricata* (Turcz.) Schischk. | Apiaceae | *Saposhnikoviae Radix* | Root |
| 22 | Fuling ‡ | *Wolfiporia cocos* (F.A.Wolf) Ryvarden & Gilb. (syn. *Poria cocos* (Schw.) Wolf) | Polyporaceae (Fungi) | *Poria* | Sclerotium |
| 23 | Gegen / Fenge | *Pueraria montana* var. *thomsonii* (Benth.) M.R.Almeida (syn. *P. thomsonii* Benth.) | Fabaceae | *Puerariae Thomsonii Radix* | Root |
| 24 | Guijianyu | *Euonymus alatus* (Thunb.) Siebold | Celastraceae | *Euonymi Ramulus Alatus* | Winged twig |
| 25 | Hehuanhua | *Albizia julibrissin* Durazz. | Fabaceae | *Albiziae Flos* | Flower / flower bud |
| 26 | Heshouwu | *Reynoutria multiflora* (Thunb.) Moldenke (syn. *Polygonum multiflorum* Thunb.; *Fallopia multiflora* (Thunb.) Haraldson) | Polygonaceae | *Polygoni Multiflori Radix* | Root tuber |
| 27 | Honghua | *Carthamus tinctorius* L. | Asteraceae | *Carthami Flos* | Flower |
| 28 | Houpo | *Houpoëa officinalis* (Rehder & E.H.Wilson) N.H.Xia & C.Y.Wu (syn. *Magnolia officinalis* Rehder & E.H.Wilson) | Magnoliaceae | *Magnoliae Officinalis Cortex* | Bark |
| 29 | Huangqi | *Astragalus mongholicus* Bunge (syn. *A. membranaceus* (Fisch.) Bunge) | Fabaceae | *Astragali Radix* | Root |
| 30 | Jinyinhua | *Lonicera japonica* Thunb. | Caprifoliaceae | *Lonicerae Japonicae Flos* | Flower bud |
| 31 | Jixueteng | *Spatholobus suberectus* Dunn | Fabaceae | *Spatholobi Caulis* | Stem |
| 32 | Kushen | *Sophora flavescens* Aiton | Fabaceae | *Sophorae Flavescentis Radix* | Root |
| 33 | Lianqiao | *Forsythia suspensa* (Thunb.) Vahl | Oleaceae | *Forsythiae Fructus* | Fruit |
| 34 | Maoxucao | *Orthosiphon aristatus* (Blume) Miq. (syn. *Clerodendranthus spicatus* (Thunb.) C.Y.Wu) | Lamiaceae | *Clerodendranthi Spicati Herba* | Aerial part |
| 35 | Fuzi / Paofuzi | *Aconitum carmichaelii* Debeaux | Ranunculaceae | *Aconiti Lateralis Radix Praeparata* | Prepared lateral root |
| 36 | Roucongrong | *Cistanche deserticola* Y.C.Ma | Orobanchaceae | *Cistanches Herba* | Fleshy stem |
| 37 | Rougui | *Cinnamomum cassia* (L.) J.Presl (syn. *C. aromaticum* Nees) | Lauraceae | *Cinnamomi Cortex* | Bark |
| 38 | Shanyao | *Dioscorea polystachya* Turcz. (syn. *D. opposita* Thunb.) | Dioscoreaceae | *Dioscoreae Rhizoma* | Rhizome |
| 39 | Shengma | *Actaea cimicifuga* L. (syn. *Cimicifuga foetida* L.) | Ranunculaceae | *Cimicifugae Rhizoma* | Rhizome |
| 40 | Shihu | *Dendrobium nobile* Lindl. | Orchidaceae | *Dendrobii Caulis* | Stem |
| 41 | Suanzaoren | *Ziziphus jujuba* Mill. var. *spinosa* (Bunge) Hu ex H.F.Chow | Rhamnaceae | *Ziziphi Spinosae Semen* | Seed |
| 42 | Taizishen | *Pseudostellaria heterophylla* (Miq.) Pax | Caryophyllaceae | *Pseudostellariae Radix* | Root tuber |
| 43 | Taoren | *Prunus persica* (L.) Batsch (syn. *Amygdalus persica* L.) | Rosaceae | *Persicae Semen* | Seed |
| 44 | Tufuling | *Smilax glabra* Roxb. | Smilacaceae | *Smilacis Glabrae Rhizoma* | Rhizome |
| 45 | Wumei | *Prunus mume* Siebold & Zucc. (syn. *Armeniaca mume* Siebold) | Rosaceae | *Mume Fructus* | Fruit |
| 46 | Wushaoshe | *Ptyas dhumnades* (Cantor, 1842) (syn. *Zaocys dhumnades*) | Colubridae (Animalia) | *Zaocys* | Body (viscera removed) |
| 47 | Yanhusuo | *Corydalis yanhusuo* W.T.Wang ex Z.Y.Su & C.Y.Wu | Papaveraceae | *Corydalis Rhizoma* | Tuber |
| 48 | Yiyiren | *Coix lacryma-jobi* L. | Poaceae | *Coicis Semen* | Seed |
| 49 | Yinchen | *Artemisia capillaris* Thunb. | Asteraceae | *Artemisiae Scopariae Herba* | Aerial part |
| 50 | Yuanzhi | *Polygala tenuifolia* Willd. | Polygalaceae | *Polygalae Radix* | Root |
| 51 | Yujin | *Curcuma wenyujin* Y.H.Chen & C.Ling | Zingiberaceae | *Curcumae Radix* | Root tuber |
| 52 | Yuzhu | *Polygonatum odoratum* (Mill.) Druce | Asparagaceae | *Polygonati Odorati Rhizoma* | Rhizome |
| 53 | Zhimu | *Anemarrhena asphodeloides* Bunge | Asparagaceae | *Anemarrhenae Rhizoma* | Rhizome |
| 54 | Zhishi | *Citrus aurantium* L. | Rutaceae | *Aurantii Fructus Immaturus* | Immature fruit |

**Notes.** † Drug of animal origin (zoological); ‡ drug of fungal origin (mycological). Guizhi (Cinnamomi Ramulus) and Rougui (Cinnamomi Cortex) are derived from the same species (Cinnamomum cassia) but represent different medicinal parts (twig vs. bark).

## The components of the original CHGZGJT or modified CHGZGJT

| **Study** | **Formula** | **Components** |
| --- | --- | --- |
| Wang et al., 2009 | Modified CHGZGJT | *Bupleuri Radix* (Chaihu) 10 g, *Cinnamomi Ramulus* (Guizhi) 10 g, *Zingiberis Rhizoma* (Ganjiang) 8 g, *Trichosanthis Radix* (Tianhuafen) 12 g, *Scutellariae Radix* (Huangqin) 10 g, *Ostreae Concha* (Muli) 10 g, *Astragali Radix* (Huangqi) 15 g, *Dioscoreae Rhizoma* (Shanyao) 15 g, *Polygoni Multiflori Radix* (Heshouwu) 12 g, *Atractylodis Rhizoma* (Cangzhu) 15 g, *Salviae Miltiorrhizae Radix et Rhizoma* (Danshen) 15 g. |
| Zhang, 2014 | Modified CHGZGJT | *Bupleuri Radix* (Chaihu) 10 g, *Zingiberis Rhizoma* (Ganjiang) 8 g, *Cinnamomi Ramulus* (Guizhi) 10 g, *Trichosanthis Radix* (Tianhuafen) 12 g, *Ostreae Concha* (Muli) 10 g, *Scutellariae Radix* (Huangqin) 10 g, *Astragali Radix* (Huangqi) 15 g, *Polygoni Multiflori Radix* (Heshouwu) 12 g, *Dioscoreae Rhizoma* (Shanyao) 15 g, *Salviae Miltiorrhizae Radix et Rhizoma* (Danshen) 15 g, *Atractylodis Rhizoma* (Cangzhu) 15 g. If nausea occurred, *Pinelliae Rhizoma Praeparatum* (Fabanxia) 12 g was added; If constipation occurred, *Cistanches Herba* (Roucongrong) 20 g was added; If limb numbness occurred, *Spatholobi Caulis* (Jixueteng) 20 g and *Pheretima* (Dilong) 10 g were added; If accompanied by coronary heart disease, *Chuanxiong Rhizoma* (Chuanxiong) 15 g was added. |
| Mao et al., 2017 | Modified CHGZGJT | *Bupleuri Radix* (Chaihu) 12 g, *Cinnamomi Ramulus* (Guizhi) 10 g, *Zingiberis Rhizoma* (Ganjiang) 6-10 g, *Scutellariae Radix* (Huangqin) 6-10 g, *Glycyrrhizae Radix et Rhizoma* (Zhigancao) 6 g, *Trichosanthis Radix* (Tianhuafen) 12 g, *Ostreae Concha* (Muli) 10 g. If gastric distension occurred, *Magnoliae Officinalis Cortex* (Houpo) 10 g and *Citri Reticulatae Pericarpium* (Chenpi) 6 g were added; If chronic diarrhea occurred, *Mume Fructus* (Wumei) 15 g and *Cimicifugae Rhizoma* (Shengma) 10 g were added; If sublingual collateral tortuosity occurred, *Persicae Semen* (Taoren) 10 g, *Carthami Flos* (Honghua) 10 g and *Salviae Miltiorrhizae Radix et Rhizoma* (Danshen) 15 g were added. |
| Li, 2019 | Modified CHGZGJT | *Bupleuri Radix* (Chaihu) 15 g, *Scutellariae Radix* (Huangqin) 10 g, *Glycyrrhizae Radix et Rhizoma* (Zhigancao) 6 g, *Zingiberis Rhizoma* (Ganjiang) 10 g, *Ostreae Concha* (Muli) 30 g, *Trichosanthis Radix* (Tianhuafen) 24 g, *Cinnamomi Ramulus* (Guizhi) 12 g, *Atractylodis Rhizoma* (Cangzhu) 12 g, *Smilacis Glabrae Rhizoma* (Tufuling) 30 g, *Coicis Semen* (Yiyiren) 30 g, *Euonymi Ramulus Alatus* (Guijianyu) 30 g. |
| Lv et al., 2019 | Modified CHGZGJT | *Bupleuri Radix* (Chaihu) 15 g, *Zingiberis Rhizoma* (Ganjiang) 6 g, *Scutellariae Radix* (Huangqin) 15 g, *Glycyrrhizae Radix et Rhizoma* (Zhigancao) 15 g, *Ostreae Concha* (Muli) 15 g, *Trichosanthis Radix* (Tianhuafen) 30 g, *Cinnamomi Ramulus* (Guizhi) 15 g, *Atractylodis Macrocephalae Rhizoma* (Baizhu) 20 g, *Puerariae Thomsonii Radix* (Gegen) 30 g, *Dioscoreae Rhizoma* (Shanyao) 30 g, *Cinnamomi Cortex* (Rougui) 6 g, *Dendrobii Caulis* (Shihu) 15 g. If shortness of breath and fatigue were identified, *Codonopsis Radix* (Dangshen) and *Astragali Radix* (Huangqi) were added; If excessive thirst was identified, *Anemarrhenae Rhizoma* (Zhimu) and *Polygonati Odorati Rhizoma* (Yuzhu) were added. |
| Liu, 2022 | Modified CHGZGJT | *Bupleuri Radix* (Chaihu) 10 g, *Zingiberis Rhizoma* (Ganjiang) 6 g, *Scutellariae Radix* (Huangqin) 10 g, *Glycyrrhizae Radix et Rhizoma* (Zhigancao) 6 g, *Ostreae Concha* (Muli, decocted first) 15 g, *Trichosanthis Radix* (Tianhuafen) 15 g, *Cinnamomi Ramulus* (Guizhi) 10 g. If shortness of breath and fatigue were identified, *Codonopsis Radix* (Dangshen) and *Astragali Radix* (Huangqi) were added; If insomnia was identified, *Ziziphi Spinosae Semen* (Suanzaoren), *Albiziae Flos* (Hehuanhua) and *Polygalae Radix* (Yuanzhi) were added; If loose stools were identified, *Atractylodis Macrocephalae Rhizoma* (Baizhu) and *Puerariae Thomsonii Radix* (Gegen) were added. |
| Zhao, 2022 | Original CHGZGJT | *Bupleuri Radix* (Chaihu) 15 g, *Cinnamomi Ramulus* (Guizhi) 12 g, *Zingiberis Rhizoma* (Ganjiang) 6 g, *Scutellariae Radix* (Huangqin) 9 g, *Ostreae Concha* (Muli) 15 g, *Trichosanthis Radix* (Tianhuafen) 15 g, *Glycyrrhizae Radix et Rhizoma* (Zhigancao) 6 g. |
| Zhao, 2023 | Modified CHGZGJT | *Bupleuri Radix* (Chaihu) 15 g, *Zingiberis Rhizoma* (Ganjiang) 10 g, *Scutellariae Radix* (Huangqin) 15 g, *Glycyrrhizae Radix et Rhizoma* (Zhigancao) 15 g, *Ostreae Concha* (Muli) 15 g, *Trichosanthis Radix* (Tianhuafen) 30 g, *Cinnamomi Ramulus* (Guizhi) 15 g, *Atractylodis Macrocephalae Rhizoma* (Baizhu) 20 g, *Puerariae Thomsonii Radix* (Gegen) 30 g, *Dioscoreae Rhizoma* (Shanyao) 30 g, *Cinnamomi Cortex* (Rougui) 6 g, *Dendrobii Caulis* (Shihu) 15 g. |
| Zheng et al., 2023 | Modified CHGZGJT | *Ostreae Concha* (Muli, decocted first) 30 g, *Trichosanthis Radix* (Tianhuafen) 20 g, *Paeoniae Radix Alba* (Baishao) 15 g, *Bupleuri Radix* (Chaihu) 10 g, *Scutellariae Radix* (Huangqin) 10 g, *Poria* (Fuling) 10 g, *Moutan Cortex* (Mudanpi) 10 g, *Paeoniae Radix Rubra* (Chishao) 10 g, *Persicae Semen* (Taoren) 10 g, *Cinnamomi Ramulus* (Guizhi) 6 g, *Zingiberis Rhizoma* (Ganjiang) 6 g, *Glycyrrhizae Radix et Rhizoma* (Gancao) 6 g, *Zaocys* (Wushaoshe) 5 g, *Saposhnikoviae Radix* (Fangfeng) 5 g. If qi deficiency was identified, *Pseudostellariae Radix* (Taizishen) 20-30 g was added; If yang deficiency was identified, *Cinnamomi Ramulus* (Guizhi) was removed and *Cinnamomi Cortex* (Rougui) was added; If skin infection from scratching occurred, *Lonicerae Japonicae Flos* (Jinyinhua) 15 g and *Forsythiae Fructus* (Lianqiao) 15 g were added; If fungal infection occurred, *Sophorae Flavescentis Radix* (Kushen) 15 g and *Dictamni Cortex* (Baixianpi) 15 g were added. |
| Xiang et al., 2024 | Modified CHGZGJT | *Atractylodis Macrocephalae Rhizoma* (Baizhu) 20 g, *Bupleuri Radix* (Chaihu) 15 g, *Scutellariae Radix* (Huangqin) 15 g, *Glycyrrhizae Radix et Rhizoma* (Zhigancao) 15 g, *Cinnamomi Ramulus* (Guizhi) 15 g, *Ostreae Concha* (Muli) 15 g, *Dendrobii Caulis* (Shihu) 15 g, *Zingiberis Rhizoma* (Ganjiang) 6 g. |
| Zhang, 2024 | Modified CHGZGJT | *Bupleuri Radix* (Chaihu) 10 g, *Scutellariae Radix* (Huangqin) 10 g, *Trichosanthis Radix* (Tianhuafen) 10 g, *Clerodendranthi Spicati Herba* (Maoxucao) 10 g, *Cinnamomi Ramulus* (Guizhi) 6 g, *Zingiberis Rhizoma* (Ganjiang) 6 g, *Glycyrrhizae Radix et Rhizoma* (Zhigancao) 6 g, *Ostreae Concha* (Muli) 30 g. If severe abdominal pain occurred, *Corydalis Rhizoma* (Yanhusuo) 10 g and *Toosendan Fructus* (Chuanlianzi) 10 g were added; If severe spleen deficiency occurred, *Dioscoreae Rhizoma* (Shanyao) 15 g and *Atractylodis Macrocephalae Rhizoma* (Baizhu) 15 g were added; If diarrhea with severe soreness and cold pain in the waist and knees occurred, *Cinnamomi Cortex* (Rougui) 3 g and *Aconiti Lateralis Radix Praeparata* (Paofuzi) 10 g were added; If severe damp-heat occurred, *Artemisiae Scopariae Herba* (Yinchen) 10 g and *Curcumae Radix* (Yujin) 10 g were added. |
| Rong et al., 2025 | Modified CHGZGJT | *Bupleuri Radix* (Chaihu) 20 g, *Scutellariae Radix* (Huangqin) 10 g, *Cinnamomi Ramulus* (Guizhi) 10 g, *Zingiberis Rhizoma* (Ganjiang) 5 g, *Trichosanthis Radix* (Tianhuafen) 15 g, *Glycyrrhizae Radix et Rhizoma* (Zhigancao) 5 g, *Ostreae Concha* (Muli) 30 g, *Aurantii Fructus Immaturus* (Zhishi) 15 g, *Atractylodis Macrocephalae Rhizoma* (Baizhu) 30 g. |
