# Supplementary material for: The efficacy and safety of Chaihu guizhi ganjiang tang for type 2 diabetes mellitus: a systematic review and meta-analysis
Source: Front Pharmacol. 2026 Jun 25;17:1855616. doi: 10.3389/fphar.2026.1855616 (PMC13345826; doi:10.3389/fphar.2026.1855616)
Supplement: Supplementary file 2 [file Supplementaryfile2.docx]

Supplementary Material

**Contents Page**

[1 Supplementary Material S1. PRISMA 2020 checklist 2](#_Toc231329431)

[2 Supplementary Material S2. Database and Search Strategies 8](#_Toc231329432)

[2.1 PubMed 8](#_Toc231329433)

[2.2 Embase 9](#_Toc231329434)

[2.3 Cochrane 11](#_Toc231329435)

[2.4 WOS 11](#_Toc231329436)

[2.5 CNKI 12](#_Toc231329437)

[2.6 Wan Fang 13](#_Toc231329438)

[2.7 VIP 14](#_Toc231329439)

[2.8 CBM 15](#_Toc231329440)

[2.9 ClinicalTrials.gov 16](#_Toc231329441)

[2.10 Chinese Clinical Trial Registry 17](#_Toc231329442)

[3 Supplementary Material S3. Meta regression 18](#_Toc231329443)

[3.1 Meta regression of HbA1c for CHGZGJT combined with conventional treatment vs. conventional treatment 18](#_Toc231329444)

[3.2 Meta regression of FPG for CHGZGJT combined with conventional treatment vs. conventional treatment 18](#_Toc231329445)

[3.3 Meta regression of 2hPG for CHGZGJT combined with conventional treatment vs. conventional treatment 19](#_Toc231329446)

[4 Supplementary Material S4. Subgroup analysis 20](#_Toc231329447)

[4.1 Subgroup analysis of HbA1c for CHGZGJT combined with conventional treatment vs. conventional treatment 20](#_Toc231329448)

[4.2 Subgroup analysis of FPG for CHGZGJT combined with conventional treatment vs. conventional treatment 21](#_Toc231329449)

[4.3 Subgroup analysis of 2hPG for CHGZGJT combined with conventional treatment vs. conventional treatment 21](#_Toc231329450)

[5 Supplementary Material S5. Sensitivity analysis 22](#_Toc231329451)

[6 Supplementary Material S6. Adverse events and safety monitoring reported in the included studies 28](#_Toc231329452)

[7 Supplementary Material S7. Restricted analysis: influence of the two non-randomized controlled studies (Lv et al., 2019; Liu, 2022) on the pooled estimates 29](#_Toc231329453)

[8 Supplementary Material S8. Summary of findings and GRADE certainty of the evidence for CHGZGJT combined with conventional therapy versus conventional therapy alone in type 2 diabetes mellitus 30](#_Toc231329454)

[9 Supplementary Material S9. Egger’s test of HbA1c, FPG and 2hPG 32](#_Toc231329455)

# Supplementary Material S1. PRISMA 2020 checklist

| **Section and Topic** | **Item #** | **Checklist item** | **Location where item is reported** |
| --- | --- | --- | --- |
| **TITLE** | | |  |
| Title | 1 | Identify the report as a systematic review. |  |
| **ABSTRACT** | | |  |
| Abstract | 2 | See the PRISMA 2020 for Abstracts checklist. |  |
| **INTRODUCTION** | | |  |
| Rationale | 3 | Describe the rationale for the review in the context of existing knowledge. |  |
| Objectives | 4 | Provide an explicit statement of the objective(s) or question(s) the review addresses. |  |
| **METHODS** | | |  |
| Eligibility criteria | 5 | Specify the inclusion and exclusion criteria for the review and how studies were grouped for the syntheses. |  |
| Information sources | 6 | Specify all databases, registers, websites, organisations, reference lists and other sources searched or consulted to identify studies. Specify the date when each source was last searched or consulted. |  |
| Search strategy | 7 | Present the full search strategies for all databases, registers and websites, including any filters and limits used. |  |
| Selection process | 8 | Specify the methods used to decide whether a study met the inclusion criteria of the review, including how many reviewers screened each record and each report retrieved, whether they worked independently, and if applicable, details of automation tools used in the process. |  |
| Data collection process | 9 | Specify the methods used to collect data from reports, including how many reviewers collected data from each report, whether they worked independently, any processes for obtaining or confirming data from study investigators, and if applicable, details of automation tools used in the process. |  |
| Data items | 10a | List and define all outcomes for which data were sought. Specify whether all results that were compatible with each outcome domain in each study were sought (e.g. for all measures, time points, analyses), and if not, the methods used to decide which results to collect. |  |
|  | 10b | List and define all other variables for which data were sought (e.g. participant and intervention characteristics, funding sources). Describe any assumptions made about any missing or unclear information. |  |
| Study risk of bias assessment | 11 | Specify the methods used to assess risk of bias in the included studies, including details of the tool(s) used, how many reviewers assessed each study and whether they worked independently, and if applicable, details of automation tools used in the process. |  |
| Effect measures | 12 | Specify for each outcome the effect measure(s) (e.g. risk ratio, mean difference) used in the synthesis or presentation of results. |  |
| Synthesis methods | 13a | Describe the processes used to decide which studies were eligible for each synthesis (e.g. tabulating the study intervention characteristics and comparing against the planned groups for each synthesis (item #5)). |  |
|  | 13b | Describe any methods required to prepare the data for presentation or synthesis, such as handling of missing summary statistics, or data conversions. |  |
|  | 13c | Describe any methods used to tabulate or visually display results of individual studies and syntheses. |  |
|  | 13d | Describe any methods used to synthesize results and provide a rationale for the choice(s). If meta-analysis was performed, describe the model(s), method(s) to identify the presence and extent of statistical heterogeneity, and software package(s) used. |  |
|  | 13e | Describe any methods used to explore possible causes of heterogeneity among study results (e.g. subgroup analysis, meta-regression). |  |
|  | 13f | Describe any sensitivity analyses conducted to assess robustness of the synthesized results. |  |
| Reporting bias assessment | 14 | Describe any methods used to assess risk of bias due to missing results in a synthesis (arising from reporting biases). |  |
| Certainty assessment | 15 | Describe any methods used to assess certainty (or confidence) in the body of evidence for an outcome. |  |
| **RESULTS** | | |  |
| Study selection | 16a | Describe the results of the search and selection process, from the number of records identified in the search to the number of studies included in the review, ideally using a flow diagram. |  |
|  | 16b | Cite studies that might appear to meet the inclusion criteria, but which were excluded, and explain why they were excluded. |  |
| Study characteristics | 17 | Cite each included study and present its characteristics. |  |
| Risk of bias in studies | 18 | Present assessments of risk of bias for each included study. |  |
| Results of individual studies | 19 | For all outcomes, present, for each study: (a) summary statistics for each group (where appropriate) and (b) an effect estimate and its precision (e.g. confidence/credible interval), ideally using structured tables or plots. |  |
| Results of syntheses | 20a | For each synthesis, briefly summarise the characteristics and risk of bias among contributing studies. |  |
|  | 20b | Present results of all statistical syntheses conducted. If meta-analysis was done, present for each the summary estimate and its precision (e.g. confidence/credible interval) and measures of statistical heterogeneity. If comparing groups, describe the direction of the effect. |  |
|  | 20c | Present results of all investigations of possible causes of heterogeneity among study results. |  |
|  | 20d | Present results of all sensitivity analyses conducted to assess the robustness of the synthesized results. |  |
| Reporting biases | 21 | Present assessments of risk of bias due to missing results (arising from reporting biases) for each synthesis assessed. |  |
| Certainty of evidence | 22 | Present assessments of certainty (or confidence) in the body of evidence for each outcome assessed. |  |
| **DISCUSSION** | | |  |
| Discussion | 23a | Provide a general interpretation of the results in the context of other evidence. |  |
|  | 23b | Discuss any limitations of the evidence included in the review. |  |
|  | 23c | Discuss any limitations of the review processes used. |  |
|  | 23d | Discuss implications of the results for practice, policy, and future research. |  |
| **OTHER INFORMATION** | | |  |
| Registration and protocol | 24a | Provide registration information for the review, including register name and registration number, or state that the review was not registered. |  |
|  | 24b | Indicate where the review protocol can be accessed, or state that a protocol was not prepared. |  |
|  | 24c | Describe and explain any amendments to information provided at registration or in the protocol. |  |
| Support | 25 | Describe sources of financial or non-financial support for the review, and the role of the funders or sponsors in the review. |  |
| Competing interests | 26 | Declare any competing interests of review authors. |  |
| Availability of data, code and other materials | 27 | Report which of the following are publicly available and where they can be found: template data collection forms; data extracted from included studies; data used for all analyses; analytic code; any other materials used in the review. |  |

*From:*  Page MJ, McKenzie JE, Bossuyt PM, Boutron I, Hoffmann TC, Mulrow CD, et al. The PRISMA 2020 statement: an updated guideline for reporting systematic reviews. BMJ 2021;372:n71. doi: 10.1136/bmj.n71

# Supplementary Material S2. Database and Search Strategies

## PubMed

The retrieval of the PubMed database was conducted on January 24, 2026, and no records were retrieved.

| Search | Query | Results |
| --- | --- | --- |
| #5 | ((Diabetes Mellitus, Type 2[MeSH Terms]) OR ("adult onset diabetes"[Title/Abstract] OR "adult onset diabetes mellitus"[Title/Abstract] OR "Adult-Onset Diabetes Mellitus"[Title/Abstract] OR "diabetes mellitus type 2"[Title/Abstract] OR "diabetes mellitus type ii"[Title/Abstract] OR "Diabetes Mellitus, Adult Onset"[Title/Abstract] OR "Diabetes Mellitus, Adult-Onset"[Title/Abstract] OR "Diabetes Mellitus, Ketosis Resistant"[Title/Abstract] OR "Diabetes Mellitus, Ketosis-Resistant"[Title/Abstract] OR "diabetes mellitus, maturity onset"[Title/Abstract] OR "Diabetes Mellitus, Maturity-Onset"[Title/Abstract] OR "diabetes mellitus, non insulin dependent"[Title/Abstract] OR "Diabetes Mellitus, Noninsulin Dependent"[Title/Abstract] OR "Diabetes Mellitus, Noninsulin-Dependent"[Title/Abstract] OR "diabetes mellitus, non-insulin-dependent"[Title/Abstract] OR "Diabetes Mellitus, Slow Onset"[Title/Abstract] OR "Diabetes Mellitus, Slow-Onset"[Title/Abstract] OR "Diabetes Mellitus, Stable"[Title/Abstract] OR "Diabetes Mellitus, Type 2"[Title/Abstract] OR "diabetes mellitus, type II"[Title/Abstract] OR "diabetes type 2"[Title/Abstract] OR "diabetes type II"[Title/Abstract] OR "diabetes, adult onset"[Title/Abstract] OR "Diabetes, Maturity-Onset"[Title/Abstract] OR "Diabetes, Type 2"[Title/Abstract] OR "dm 2"[Title/Abstract] OR "insulin independent diabetes"[Title/Abstract] OR "insulin independent diabetes mellitus"[Title/Abstract] OR "ketosis resistant diabetes mellitus"[Title/Abstract] OR "Ketosis-Resistant Diabetes Mellitus"[Title/Abstract] OR "maturity onset diabetes"[Title/Abstract] OR "maturity onset diabetes mellitus"[Title/Abstract] OR "Maturity-Onset Diabetes"[Title/Abstract] OR "Maturity-Onset Diabetes Mellitus"[Title/Abstract] OR "MODY"[Title/Abstract] OR "niddm"[Title/Abstract] OR "NIDDM (non insulin dependent diabetes mellitus)"[Title/Abstract] OR "non insulin dependent (type 2) diabetes mellitus"[Title/Abstract] OR "non insulin dependent diabetes"[Title/Abstract] OR "non insulin dependent diabetes mellitus"[Title/Abstract] OR "noninsulin dependent (type 2) diabetes mellitus"[Title/Abstract] OR "noninsulin dependent diabetes"[Title/Abstract] OR "noninsulin dependent diabetes mellitus"[Title/Abstract] OR "Noninsulin-Dependent Diabetes Mellitus"[Title/Abstract] OR "non-insulin-dependent diabetes mellitus"[Title/Abstract] OR "Slow-Onset Diabetes Mellitus"[Title/Abstract] OR "Stable Diabetes Mellitus"[Title/Abstract] OR "T2DM"[Title/Abstract] OR "TIIDM"[Title/Abstract] OR "type 2 (insulin independent) diabetes"[Title/Abstract] OR "type 2 diabetes"[Title/Abstract] OR "type 2 diabetes mellitus"[Title/Abstract] OR "Type 2 Diabetes Mellitus"[Title/Abstract] OR "type II diabetes"[Title/Abstract] OR "type II diabetes mellitus"[Title/Abstract])) AND ("Chaihu Guizhi Ganjiang"[Title/Abstract] OR "Chaihu-Guizhi-Ganjiang"[Title/Abstract] OR "Chai Hu Gui Zhi Gan Jiang"[Title/Abstract] OR "CHGZGJ"[Title/Abstract] OR "CGJ"[Title/Abstract] OR "Bupleurum, Cinnamon Twig and Ginger Decoction"[Title/Abstract] OR "Bupleurum and Cinnamon Twig and Dried Ginger Decoction"[Title/Abstract] OR "Radix Bupleuri, Ramulus Cinnamomi and Rhizoma Zingiberis Decoction"[Title/Abstract] OR "Bupleurum Plus Cinnamon Twig and Ginger Decoction"[Title/Abstract] OR "Cinnamon Twig and Ginger Decoction Plus Bupleurum"[Title/Abstract]) | 0 |
| #4 | "Chaihu Guizhi Ganjiang"[Title/Abstract] OR "Chaihu-Guizhi-Ganjiang"[Title/Abstract] OR "Chai Hu Gui Zhi Gan Jiang"[Title/Abstract] OR "CHGZGJ"[Title/Abstract] OR "CGJ"[Title/Abstract] OR "Bupleurum, Cinnamon Twig and Ginger Decoction"[Title/Abstract] OR "Bupleurum and Cinnamon Twig and Dried Ginger Decoction"[Title/Abstract] OR "Radix Bupleuri, Ramulus Cinnamomi and Rhizoma Zingiberis Decoction"[Title/Abstract] OR "Bupleurum Plus Cinnamon Twig and Ginger Decoction"[Title/Abstract] OR "Cinnamon Twig and Ginger Decoction Plus Bupleurum"[Title/Abstract] | 70 |
| #3 | (Diabetes Mellitus, Type 2[MeSH Terms]) OR ("adult onset diabetes"[Title/Abstract] OR "adult onset diabetes mellitus"[Title/Abstract] OR "Adult-Onset Diabetes Mellitus"[Title/Abstract] OR "diabetes mellitus type 2"[Title/Abstract] OR "diabetes mellitus type ii"[Title/Abstract] OR "Diabetes Mellitus, Adult Onset"[Title/Abstract] OR "Diabetes Mellitus, Adult-Onset"[Title/Abstract] OR "Diabetes Mellitus, Ketosis Resistant"[Title/Abstract] OR "Diabetes Mellitus, Ketosis-Resistant"[Title/Abstract] OR "diabetes mellitus, maturity onset"[Title/Abstract] OR "Diabetes Mellitus, Maturity-Onset"[Title/Abstract] OR "diabetes mellitus, non insulin dependent"[Title/Abstract] OR "Diabetes Mellitus, Noninsulin Dependent"[Title/Abstract] OR "Diabetes Mellitus, Noninsulin-Dependent"[Title/Abstract] OR "diabetes mellitus, non-insulin-dependent"[Title/Abstract] OR "Diabetes Mellitus, Slow Onset"[Title/Abstract] OR "Diabetes Mellitus, Slow-Onset"[Title/Abstract] OR "Diabetes Mellitus, Stable"[Title/Abstract] OR "Diabetes Mellitus, Type 2"[Title/Abstract] OR "diabetes mellitus, type II"[Title/Abstract] OR "diabetes type 2"[Title/Abstract] OR "diabetes type II"[Title/Abstract] OR "diabetes, adult onset"[Title/Abstract] OR "Diabetes, Maturity-Onset"[Title/Abstract] OR "Diabetes, Type 2"[Title/Abstract] OR "dm 2"[Title/Abstract] OR "insulin independent diabetes"[Title/Abstract] OR "insulin independent diabetes mellitus"[Title/Abstract] OR "ketosis resistant diabetes mellitus"[Title/Abstract] OR "Ketosis-Resistant Diabetes Mellitus"[Title/Abstract] OR "maturity onset diabetes"[Title/Abstract] OR "maturity onset diabetes mellitus"[Title/Abstract] OR "Maturity-Onset Diabetes"[Title/Abstract] OR "Maturity-Onset Diabetes Mellitus"[Title/Abstract] OR "MODY"[Title/Abstract] OR "niddm"[Title/Abstract] OR "NIDDM (non insulin dependent diabetes mellitus)"[Title/Abstract] OR "non insulin dependent (type 2) diabetes mellitus"[Title/Abstract] OR "non insulin dependent diabetes"[Title/Abstract] OR "non insulin dependent diabetes mellitus"[Title/Abstract] OR "noninsulin dependent (type 2) diabetes mellitus"[Title/Abstract] OR "noninsulin dependent diabetes"[Title/Abstract] OR "noninsulin dependent diabetes mellitus"[Title/Abstract] OR "Noninsulin-Dependent Diabetes Mellitus"[Title/Abstract] OR "non-insulin-dependent diabetes mellitus"[Title/Abstract] OR "Slow-Onset Diabetes Mellitus"[Title/Abstract] OR "Stable Diabetes Mellitus"[Title/Abstract] OR "T2DM"[Title/Abstract] OR "TIIDM"[Title/Abstract] OR "type 2 (insulin independent) diabetes"[Title/Abstract] OR "type 2 diabetes"[Title/Abstract] OR "type 2 diabetes mellitus"[Title/Abstract] OR "Type 2 Diabetes Mellitus"[Title/Abstract] OR "type II diabetes"[Title/Abstract] OR "type II diabetes mellitus"[Title/Abstract]) | 284,388 |
| #2 | "adult onset diabetes"[Title/Abstract] OR "adult onset diabetes mellitus"[Title/Abstract] OR "Adult-Onset Diabetes Mellitus"[Title/Abstract] OR "diabetes mellitus type 2"[Title/Abstract] OR "diabetes mellitus type ii"[Title/Abstract] OR "Diabetes Mellitus, Adult Onset"[Title/Abstract] OR "Diabetes Mellitus, Adult-Onset"[Title/Abstract] OR "Diabetes Mellitus, Ketosis Resistant"[Title/Abstract] OR "Diabetes Mellitus, Ketosis-Resistant"[Title/Abstract] OR "diabetes mellitus, maturity onset"[Title/Abstract] OR "Diabetes Mellitus, Maturity-Onset"[Title/Abstract] OR "diabetes mellitus, non insulin dependent"[Title/Abstract] OR "Diabetes Mellitus, Noninsulin Dependent"[Title/Abstract] OR "Diabetes Mellitus, Noninsulin-Dependent"[Title/Abstract] OR "diabetes mellitus, non-insulin-dependent"[Title/Abstract] OR "Diabetes Mellitus, Slow Onset"[Title/Abstract] OR "Diabetes Mellitus, Slow-Onset"[Title/Abstract] OR "Diabetes Mellitus, Stable"[Title/Abstract] OR "Diabetes Mellitus, Type 2"[Title/Abstract] OR "diabetes mellitus, type II"[Title/Abstract] OR "diabetes type 2"[Title/Abstract] OR "diabetes type II"[Title/Abstract] OR "diabetes, adult onset"[Title/Abstract] OR "Diabetes, Maturity-Onset"[Title/Abstract] OR "Diabetes, Type 2"[Title/Abstract] OR "dm 2"[Title/Abstract] OR "insulin independent diabetes"[Title/Abstract] OR "insulin independent diabetes mellitus"[Title/Abstract] OR "ketosis resistant diabetes mellitus"[Title/Abstract] OR "Ketosis-Resistant Diabetes Mellitus"[Title/Abstract] OR "maturity onset diabetes"[Title/Abstract] OR "maturity onset diabetes mellitus"[Title/Abstract] OR "Maturity-Onset Diabetes"[Title/Abstract] OR "Maturity-Onset Diabetes Mellitus"[Title/Abstract] OR "MODY"[Title/Abstract] OR "niddm"[Title/Abstract] OR "NIDDM (non insulin dependent diabetes mellitus)"[Title/Abstract] OR "non insulin dependent (type 2) diabetes mellitus"[Title/Abstract] OR "non insulin dependent diabetes"[Title/Abstract] OR "non insulin dependent diabetes mellitus"[Title/Abstract] OR "noninsulin dependent (type 2) diabetes mellitus"[Title/Abstract] OR "noninsulin dependent diabetes"[Title/Abstract] OR "noninsulin dependent diabetes mellitus"[Title/Abstract] OR "Noninsulin-Dependent Diabetes Mellitus"[Title/Abstract] OR "non-insulin-dependent diabetes mellitus"[Title/Abstract] OR "Slow-Onset Diabetes Mellitus"[Title/Abstract] OR "Stable Diabetes Mellitus"[Title/Abstract] OR "T2DM"[Title/Abstract] OR "TIIDM"[Title/Abstract] OR "type 2 (insulin independent) diabetes"[Title/Abstract] OR "type 2 diabetes"[Title/Abstract] OR "type 2 diabetes mellitus"[Title/Abstract] OR "Type 2 Diabetes Mellitus"[Title/Abstract] OR "type II diabetes"[Title/Abstract] OR "type II diabetes mellitus"[Title/Abstract] | 237,302 |
| #1 | Diabetes Mellitus, Type 2[MeSH Terms] | 196,302 |

## Embase

The retrieval of the Embase database was conducted on January 24, 2026, and no records were retrieved.

| Search |  | Results |
| --- | --- | --- |
| #5 | #3 AND #4 | 0 |
| #4 | #1 OR #2 | 513,618 |
| #3 | ‘Chaihu Guizhi Ganjiang’:ti,ab,kw OR ‘Chaihu-Guizhi-Ganjiang’:ti,ab,kw OR ‘Chai Hu Gui Zhi Gan Jiang’:ti,ab,kw OR ‘CHGZGJ’:ti,ab,kw OR ‘CGJ’:ti,ab,kw OR ‘Bupleurum, Cinnamon Twig and Ginger Decoction’:ti,ab,kw OR ‘Bupleurum and Cinnamon Twig and Dried Ginger Decoction’:ti,ab,kw OR ‘Radix Bupleuri, Ramulus Cinnamomi and Rhizoma Zingiberis Decoction’:ti,ab,kw OR ‘Bupleurum Plus Cinnamon Twig and Ginger Decoction’:ti,ab,kw OR ‘Cinnamon Twig and Ginger Decoction Plus Bupleurum’:ti,ab,kw | 88 |
| #2 | 'adult onset diabetes':ti,ab,kw OR 'adult onset diabetes mellitus':ti,ab,kw OR 'adult-onset diabetes mellitus':ti,ab,kw OR 'diabetes mellitus type 2':ti,ab,kw OR 'diabetes mellitus type ii':ti,ab,kw OR 'diabetes mellitus, adult onset':ti,ab,kw OR 'diabetes mellitus, adult-onset':ti,ab,kw OR 'diabetes mellitus, ketosis resistant':ti,ab,kw OR 'diabetes mellitus, ketosis-resistant':ti,ab,kw OR 'diabetes mellitus, maturity onset':ti,ab,kw OR 'diabetes mellitus, maturity-onset':ti,ab,kw OR 'diabetes mellitus, non insulin dependent':ti,ab,kw OR 'diabetes mellitus, noninsulin dependent':ti,ab,kw OR 'diabetes mellitus, noninsulin-dependent':ti,ab,kw OR 'diabetes mellitus, non-insulin-dependent':ti,ab,kw OR 'diabetes mellitus, slow onset':ti,ab,kw OR 'diabetes mellitus, slow-onset':ti,ab,kw OR 'diabetes mellitus, stable':ti,ab,kw OR 'diabetes mellitus, type 2':ti,ab,kw OR 'diabetes mellitus, type ii':ti,ab,kw OR 'diabetes type 2':ti,ab,kw OR 'diabetes type ii':ti,ab,kw OR 'diabetes, adult onset':ti,ab,kw OR 'diabetes, maturity-onset':ti,ab,kw OR 'diabetes, type 2':ti,ab,kw OR 'dm 2':ti,ab,kw OR 'insulin independent diabetes':ti,ab,kw OR 'insulin independent diabetes mellitus':ti,ab,kw OR 'ketosis resistant diabetes mellitus':ti,ab,kw OR 'ketosis-resistant diabetes mellitus':ti,ab,kw OR 'maturity onset diabetes':ti,ab,kw OR 'maturity onset diabetes mellitus':ti,ab,kw OR 'maturity-onset diabetes':ti,ab,kw OR 'maturity-onset diabetes mellitus':ti,ab,kw OR 'mody':ti,ab,kw OR 'niddm':ti,ab,kw OR 'niddm (non insulin dependent diabetes mellitus)':ti,ab,kw OR 'non insulin dependent (type 2) diabetes mellitus':ti,ab,kw OR 'non insulin dependent diabetes':ti,ab,kw OR 'non insulin dependent diabetes mellitus':ti,ab,kw OR 'noninsulin dependent (type 2) diabetes mellitus':ti,ab,kw OR 'noninsulin dependent diabetes':ti,ab,kw OR 'noninsulin dependent diabetes mellitus':ti,ab,kw OR 'noninsulin-dependent diabetes mellitus':ti,ab,kw OR 'non-insulin-dependent diabetes mellitus':ti,ab,kw OR 'slow-onset diabetes mellitus':ti,ab,kw OR 'stable diabetes mellitus':ti,ab,kw OR 't2dm':ti,ab,kw OR 'tiidm':ti,ab,kw OR 'type 2 (insulin independent) diabetes':ti,ab,kw OR 'type 2 diabetes':ti,ab,kw OR 'type 2 diabetes mellitus':ti,ab,kw OR 'type ii diabetes':ti,ab,kw OR 'type ii diabetes mellitus':ti,ab,kw | 381,752 |
| #1 | 'non insulin dependent diabetes mellitus'/exp OR 'non insulin dependent diabetes mellitus' | 447,890 |


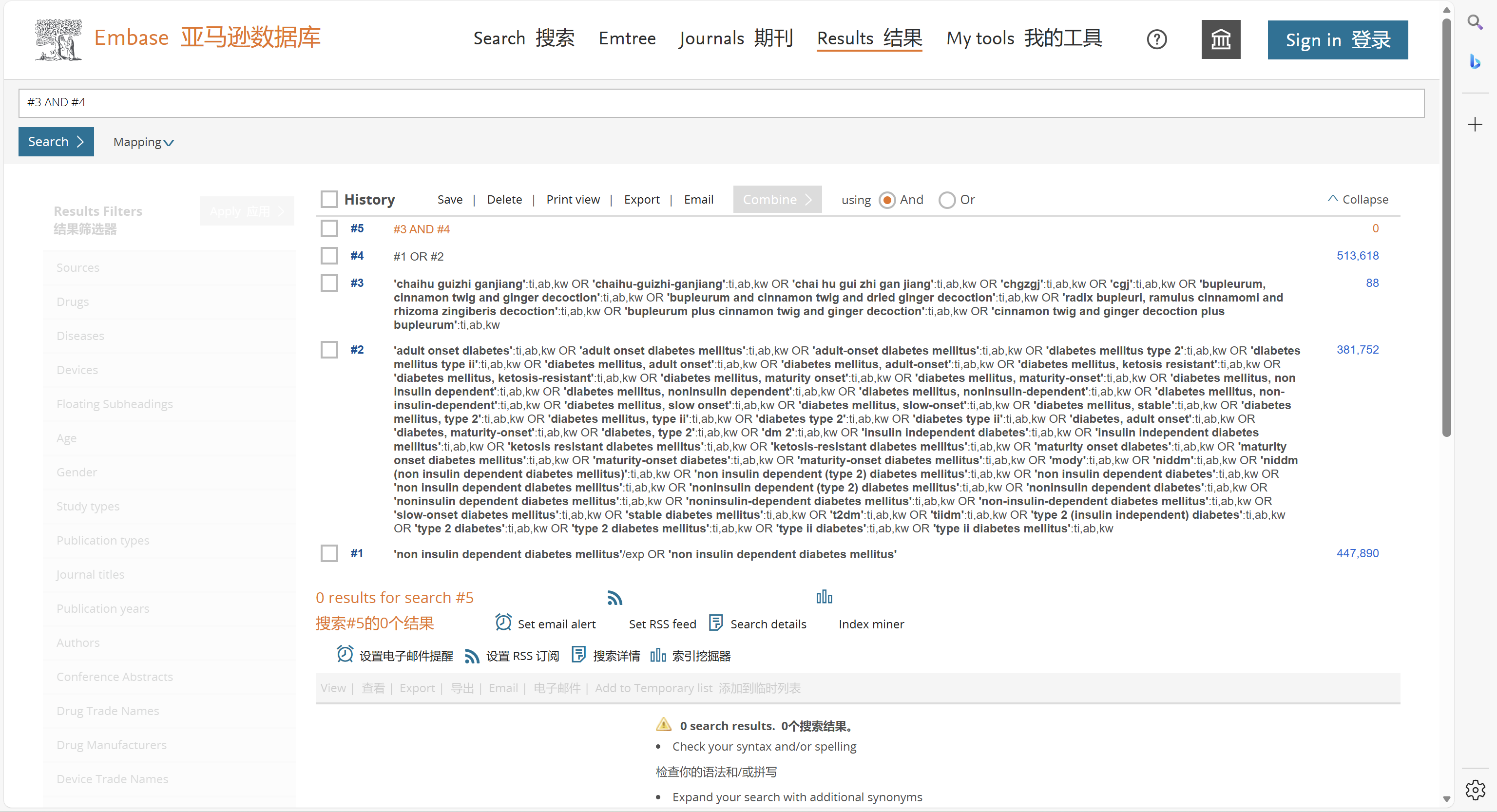


## Cochrane

The retrieval of the Cochrane Library was conducted on January 24, 2026, and 1 record were retrieved.


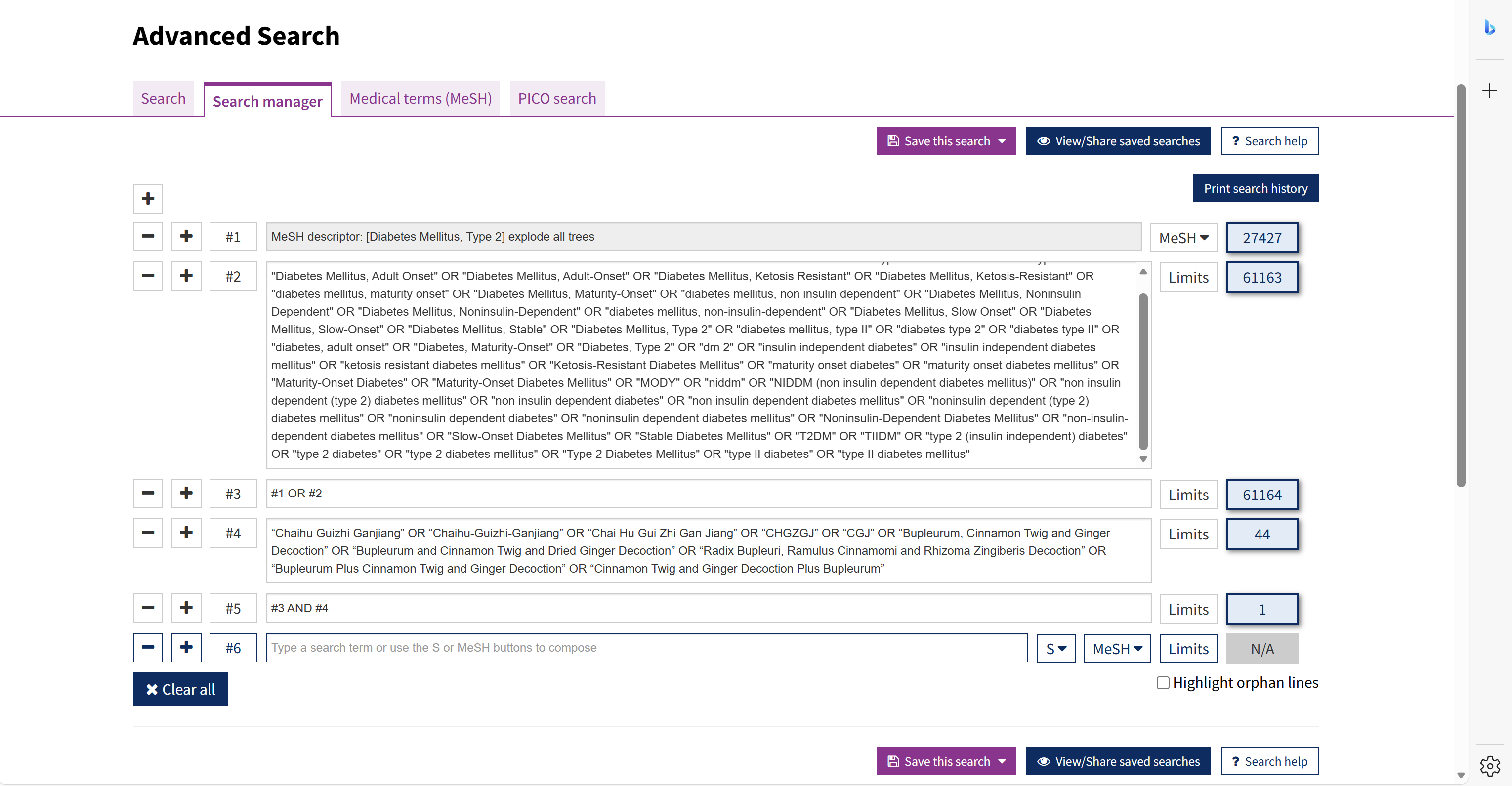


## WOS

The retrieval of the WOS database was conducted on January 24, 2026, and no records were retrieved.

| History | Search Query | Results |
| --- | --- | --- |
| #3 | #1 AND #2 | 0 |
| #2 | TS=“Chaihu Guizhi Ganjiang” OR “Chaihu-Guizhi-Ganjiang” OR “Chai Hu Gui Zhi Gan Jiang” OR “CHGZGJ” OR “CGJ” OR “Bupleurum, Cinnamon Twig and Ginger Decoction” OR “Bupleurum and Cinnamon Twig and Dried Ginger Decoction” OR “Radix Bupleuri, Ramulus Cinnamomi and Rhizoma Zingiberis Decoction” OR “Bupleurum Plus Cinnamon Twig and Ginger Decoction” OR “Cinnamon Twig and Ginger Decoction Plus Bupleurum”) | 66 |
| #1 | TS="adult onset diabetes" OR "adult onset diabetes mellitus" OR "Adult-Onset Diabetes Mellitus" OR "diabetes mellitus type 2" OR "diabetes mellitus type ii" OR "Diabetes Mellitus, Adult Onset" OR "Diabetes Mellitus, Adult-Onset" OR "Diabetes Mellitus, Ketosis Resistant" OR "Diabetes Mellitus, Ketosis-Resistant" OR "diabetes mellitus, maturity onset" OR "Diabetes Mellitus, Maturity-Onset" OR "diabetes mellitus, non insulin dependent" OR "Diabetes Mellitus, Noninsulin Dependent" OR "Diabetes Mellitus, Noninsulin-Dependent" OR "diabetes mellitus, non-insulin-dependent" OR "Diabetes Mellitus, Slow Onset" OR "Diabetes Mellitus, Slow-Onset" OR "Diabetes Mellitus, Stable" OR "Diabetes Mellitus, Type 2" OR "diabetes mellitus, type II" OR "diabetes type 2" OR "diabetes type II" OR "diabetes, adult onset" OR "Diabetes, Maturity-Onset" OR "Diabetes, Type 2" OR "dm 2" OR "insulin independent diabetes" OR "insulin independent diabetes mellitus" OR "ketosis resistant diabetes mellitus" OR "Ketosis-Resistant Diabetes Mellitus" OR "maturity onset diabetes" OR "maturity onset diabetes mellitus" OR "Maturity-Onset Diabetes" OR "Maturity-Onset Diabetes Mellitus" OR "MODY" OR "niddm" OR "NIDDM (non insulin dependent diabetes mellitus)" OR "non insulin dependent (type 2) diabetes mellitus" OR "non insulin dependent diabetes" OR "non insulin dependent diabetes mellitus" OR "noninsulin dependent (type 2) diabetes mellitus" OR "noninsulin dependent diabetes" OR "noninsulin dependent diabetes mellitus" OR "Noninsulin-Dependent Diabetes Mellitus" OR "non-insulin-dependent diabetes mellitus" OR "Slow-Onset Diabetes Mellitus" OR "Stable Diabetes Mellitus" OR "T2DM" OR "TIIDM" OR "type 2 (insulin independent) diabetes" OR "type 2 diabetes" OR "type 2 diabetes mellitus" OR "Type 2 Diabetes Mellitus" OR "type II diabetes" OR "type II diabetes mellitus" | 191,624 |


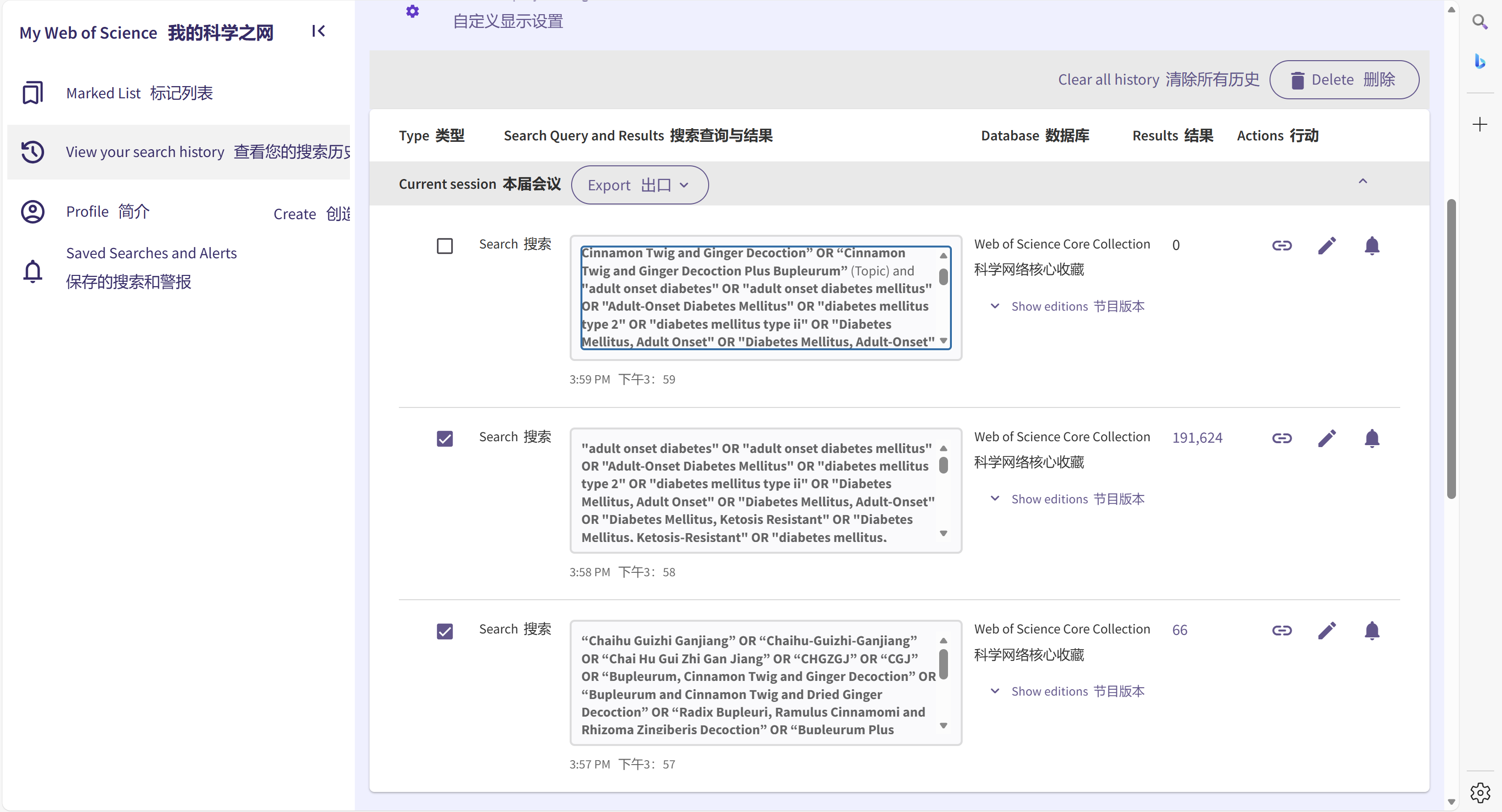


## CNKI

The retrieval of the CNKI database was conducted on January 24, 2026, and a total of 74 records were retrieved.

(SU=‘柴胡桂枝干姜’+‘柴胡桂枝干姜汤’+‘柴胡桂姜’+‘柴胡桂姜汤’+‘柴胡姜桂’+‘柴胡姜桂汤’+‘柴桂姜’+‘柴桂姜汤’ OR TKA=‘柴胡桂枝干姜’+‘柴胡桂枝干姜汤’+‘柴胡桂姜’+‘柴胡桂姜汤’+‘柴胡姜桂’+‘柴胡姜桂汤’+‘柴桂姜’+‘柴桂姜汤’) AND (SU=‘糖尿病’+‘2型糖尿病’+‘二型糖尿病’+‘Ⅱ型糖尿病’+‘2型DM’+‘非胰岛素依赖型糖尿病’+‘成人发病型糖尿病’+‘消渴’+‘消瘅’+‘T2DM’+‘DM’ OR TKA=‘糖尿病’+‘2型糖尿病’+‘二型糖尿病’+‘Ⅱ型糖尿病’+‘2型DM’+‘非胰岛素依赖型糖尿病’+‘成人发病型糖尿病’+‘消渴’+‘消瘅’+‘T2DM’+‘DM’)


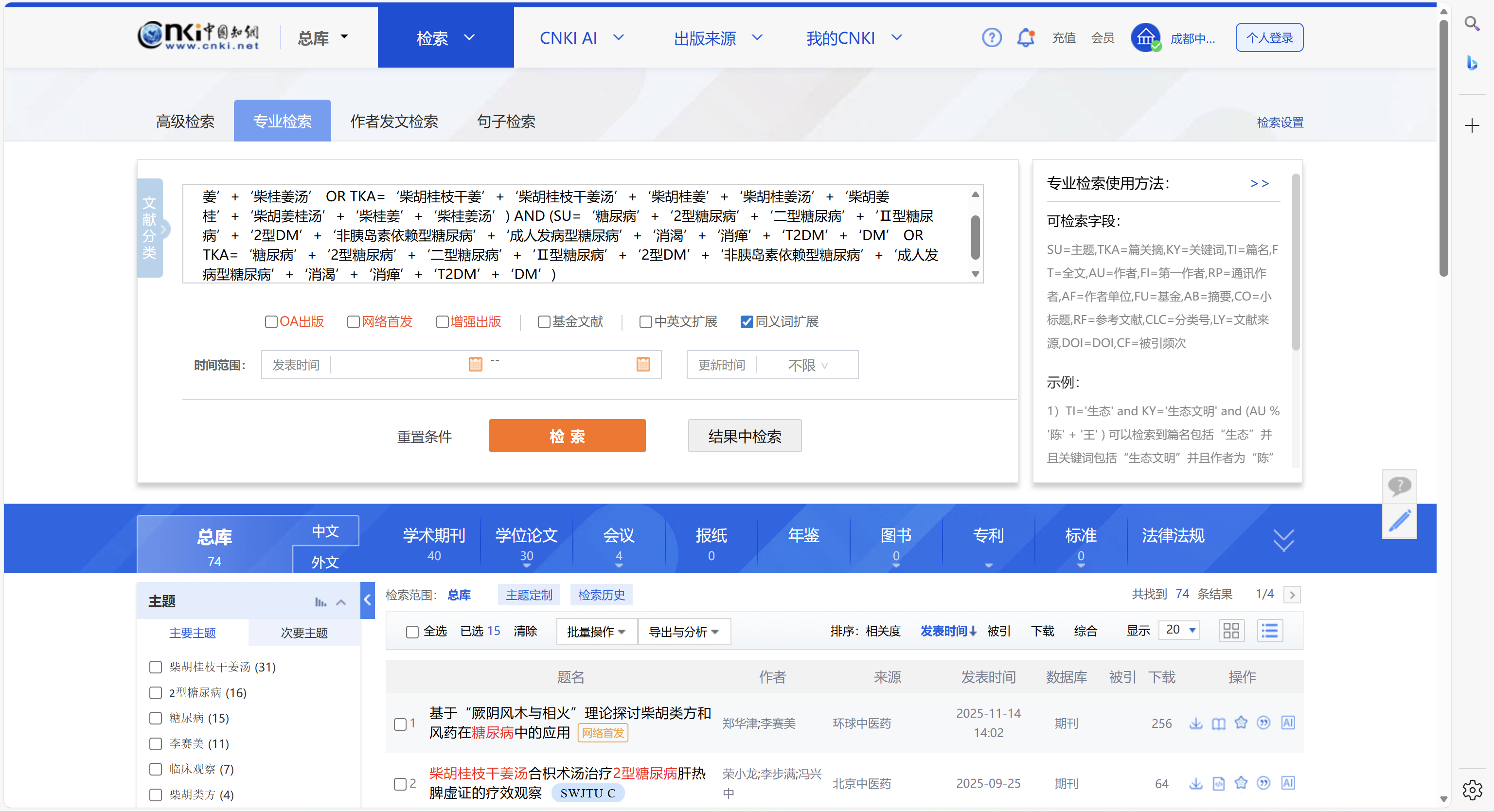


## Wan Fang

The retrieval of the Wan Fang database was conducted on January 24, 2026, and a total of 132 records were retrieved.

(主题:(柴胡桂枝干姜 or 柴胡桂枝干姜汤 or 柴胡桂姜 or 柴胡桂姜汤 or 柴胡姜桂 or 柴胡姜桂汤 or 柴桂姜 or 柴桂姜汤) or 题名或关键词:(柴胡桂枝干姜 or 柴胡桂枝干姜汤 or 柴胡桂姜 or 柴胡桂姜汤 or 柴胡姜桂 or 柴胡姜桂汤 or 柴桂姜 or 柴桂姜汤)) and (主题:(糖尿病 or 2型糖尿病 or 二型糖尿病 or Ⅱ型糖尿病 or 2型DM or 非胰岛素依赖型糖尿病 or 成人发病型糖尿病 or 消渴 or 消瘅 or T2DM or DM) or 题名或关键词:(糖尿病 or 2型糖尿病 or 二型糖尿病 or Ⅱ型糖尿病 or 2型DM or 非胰岛素依赖型糖尿病 or 成人发病型糖尿病 or 消渴 or 消瘅 or T2DM or DM))


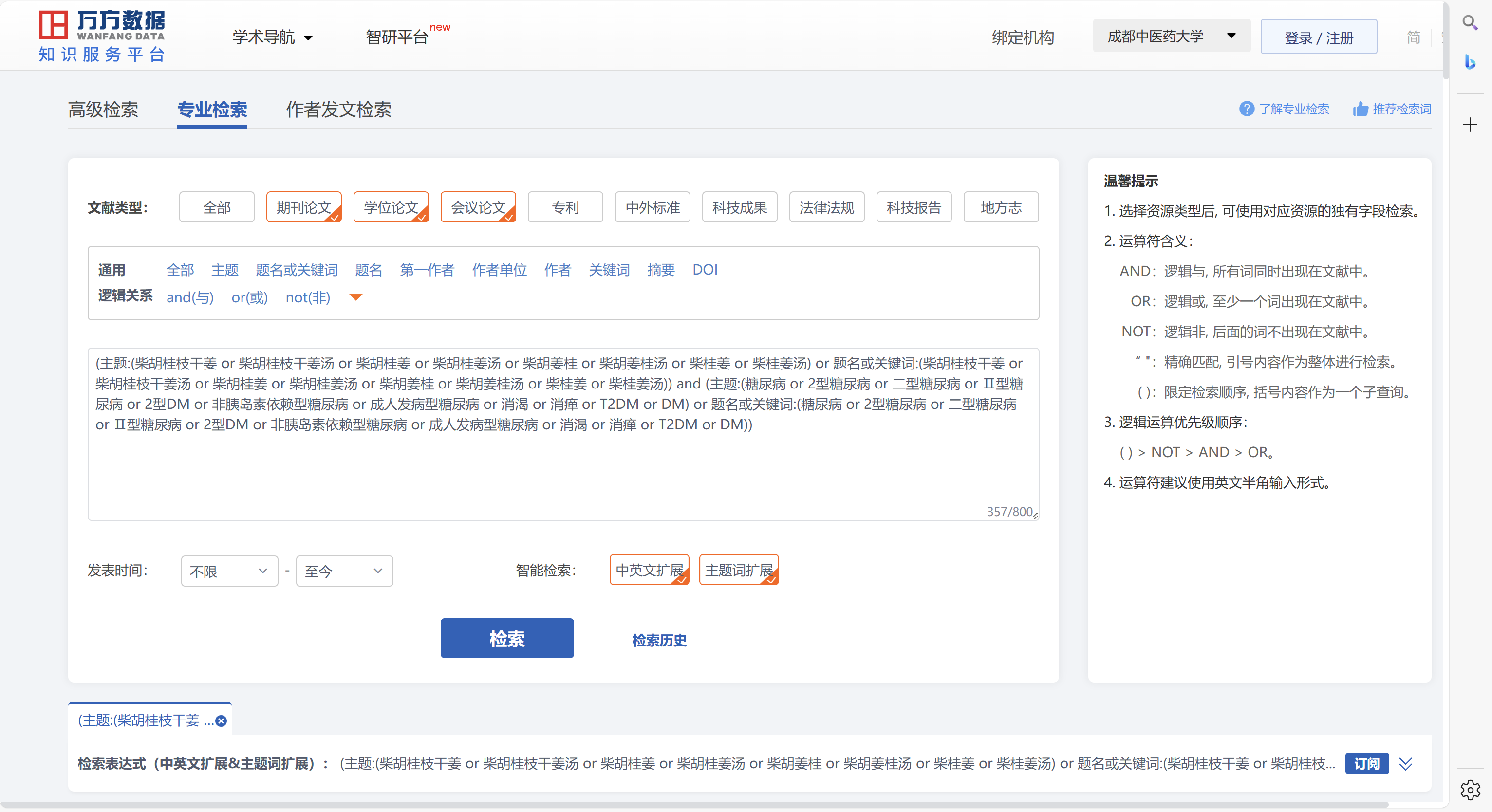


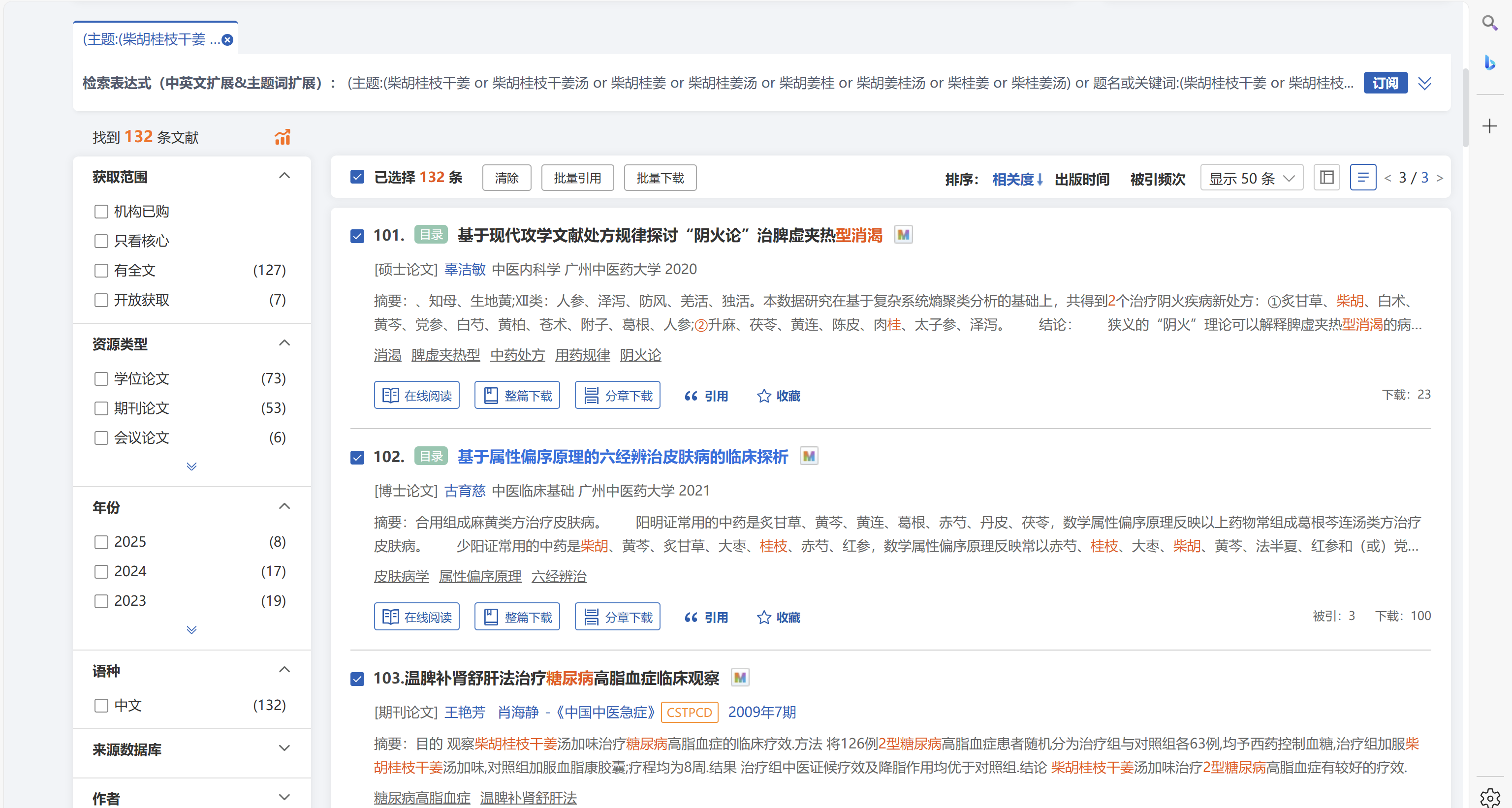


## VIP

The retrieval of the VIP database was conducted on January 24, 2026, and a total of 42 records were retrieved.

((M=(柴胡桂枝干姜+柴胡桂枝干姜汤+柴胡桂姜+柴胡桂姜汤+柴胡姜桂+柴胡姜桂汤+柴桂姜+柴桂姜汤)) OR (R=(柴胡桂枝干姜+柴胡桂枝干姜汤+柴胡桂姜+柴胡桂姜汤+柴胡姜桂+柴胡姜桂汤+柴桂姜+柴桂姜汤))) AND ((M=(糖尿病+2型糖尿病+二型糖尿病+Ⅱ型糖尿病+2型DM+非胰岛素依赖型糖尿病+成人发病型糖尿病+消渴+消瘅+T2DM+DM)) OR (R=(糖尿病+2型糖尿病+二型糖尿病+Ⅱ型糖尿病+2型DM+非胰岛素依赖型糖尿病+成人发病型糖尿病+消渴+消瘅+T2DM+DM)))


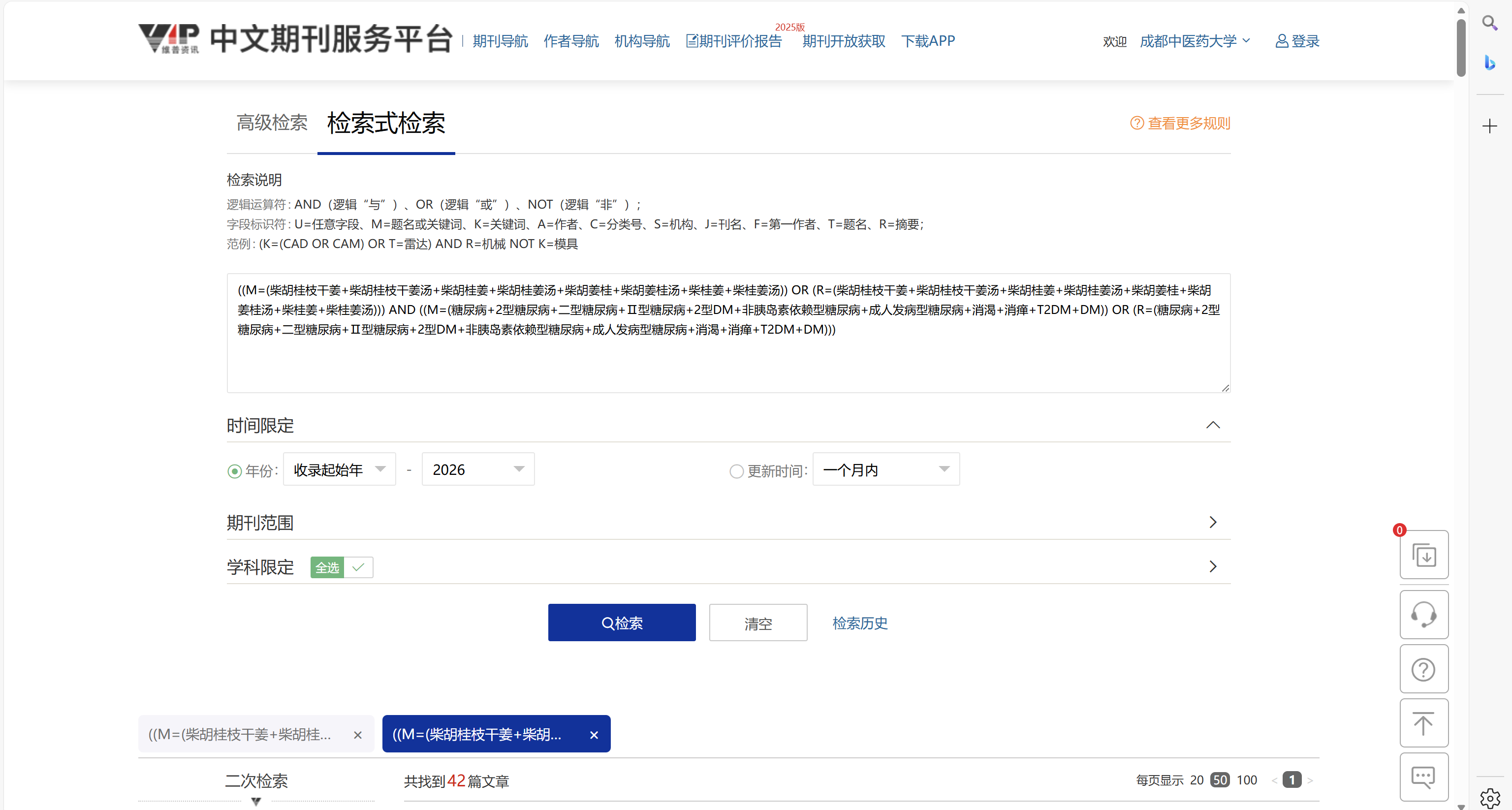


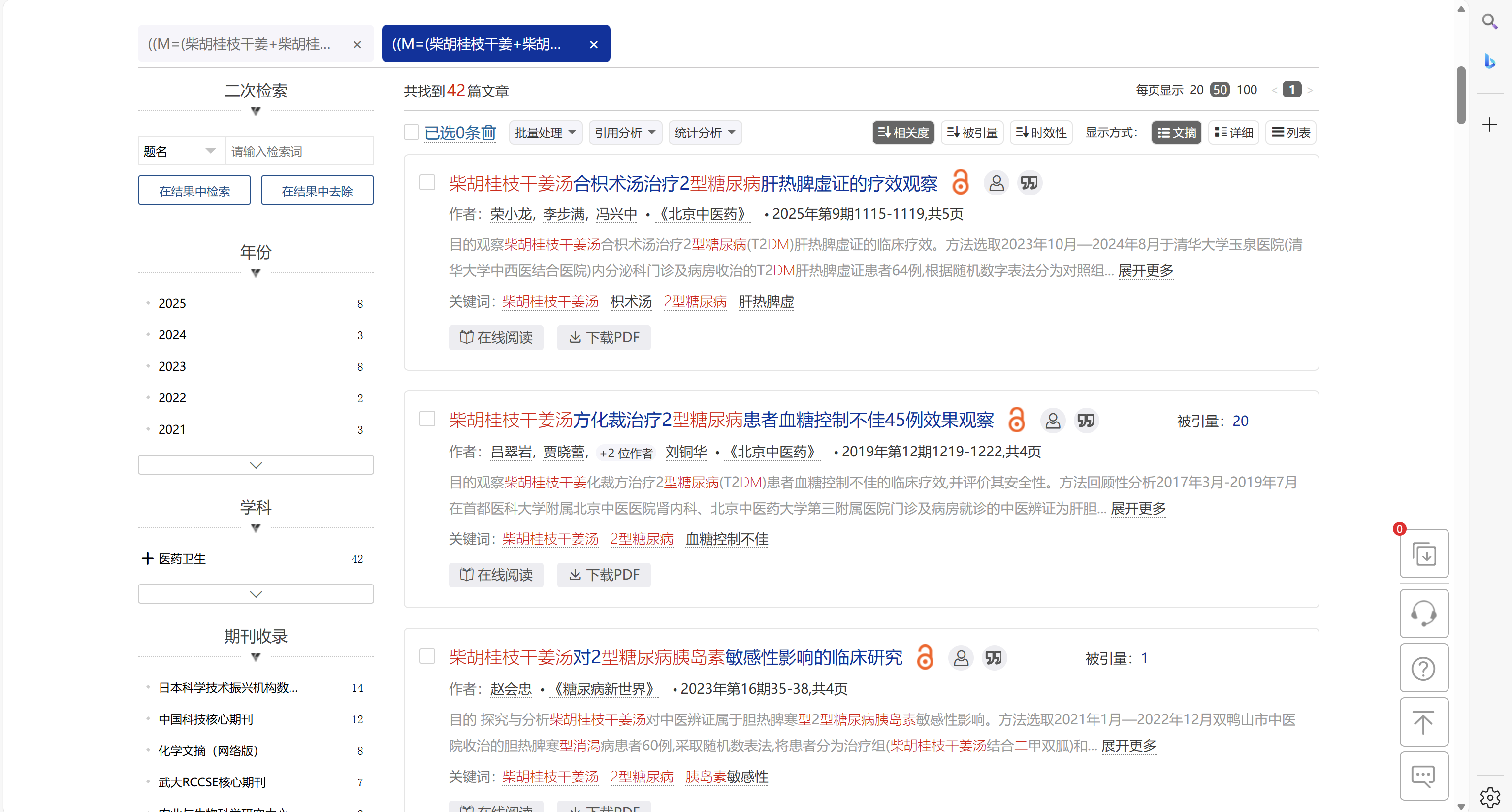


## CBM

The retrieval of the CBM database was conducted on January 24, 2026, and a total of 37 records were retrieved.

((“柴胡桂枝干姜汤”[不加权:扩展]) OR (“柴胡桂枝干姜”[常用字段:智能] OR “柴胡桂枝干姜汤”[常用字段:智能] OR “柴胡桂姜”[常用字段:智能] OR “柴胡桂姜汤”[常用字段:智能] OR “柴胡姜桂”[常用字段:智能] OR “柴胡姜桂汤”[常用字段:智能] OR “柴桂姜”[常用字段:智能] OR “柴桂姜汤”[常用字段:智能])) AND ((“糖尿病, 2型”[不加权:扩展]) OR (“糖尿病”[常用字段:智能] OR “2型糖尿病”[常用字段:智能] OR “二型糖尿病”[常用字段:智能] OR “Ⅱ型糖尿病”[常用字段:智能] OR “2型DM”[常用字段:智能] OR “非胰岛素依赖型糖尿病”[常用字段:智能] OR “成人发病型糖尿病”[常用字段:智能] OR “消渴”[常用字段:智能] OR “消瘅”[常用字段:智能] OR “T2DM”[常用字段:智能] OR “DM”[常用字段:智能]))


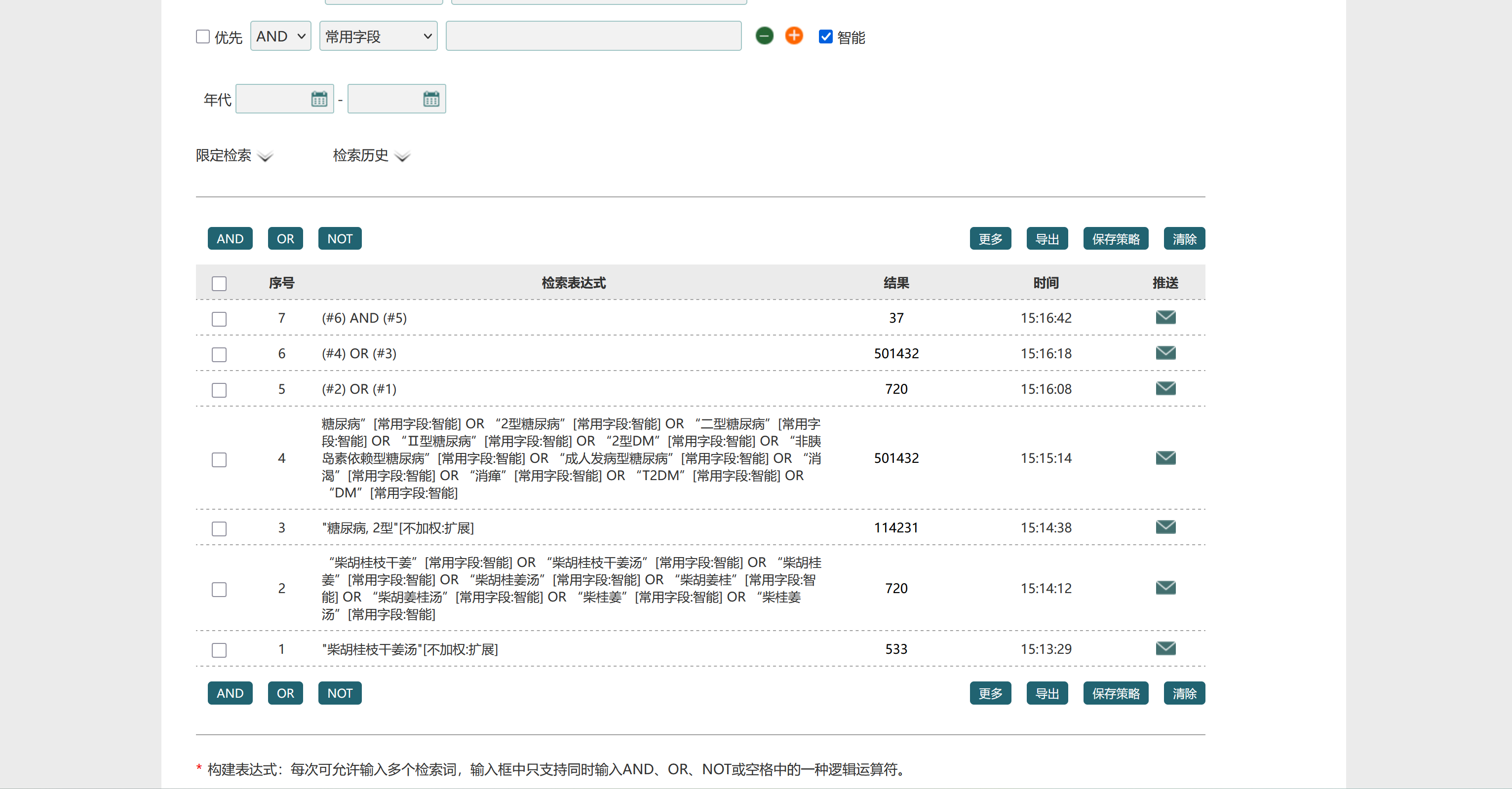


## ClinicalTrials.gov

The retrieval of the ClinicalTrials.gov database was conducted on January 24, 2026, and no records were retrieved.


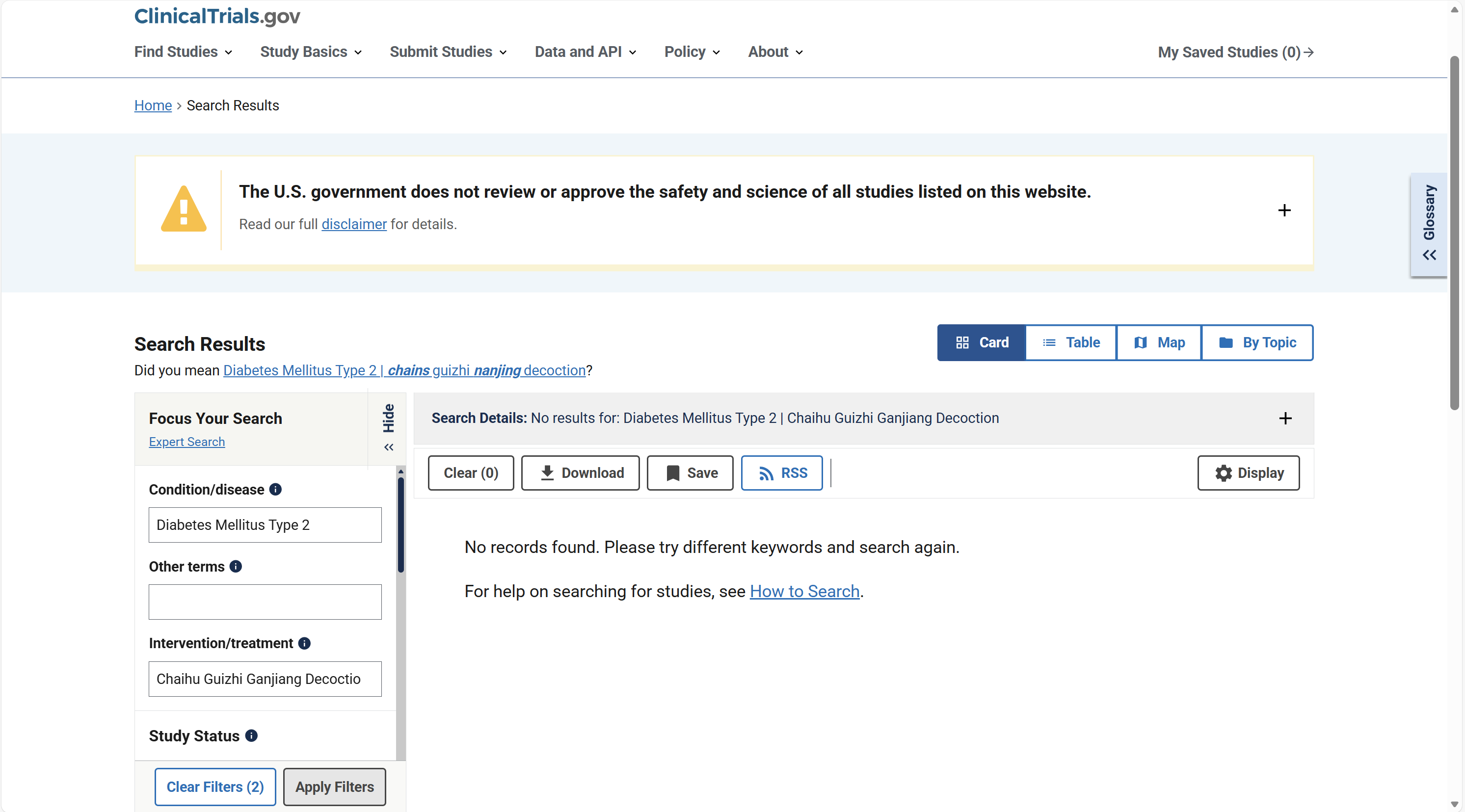


## Chinese Clinical Trial Registry

The retrieval of the Chinese Clinical Trial Registry was conducted on January 24, 2026, and no records were retrieved.


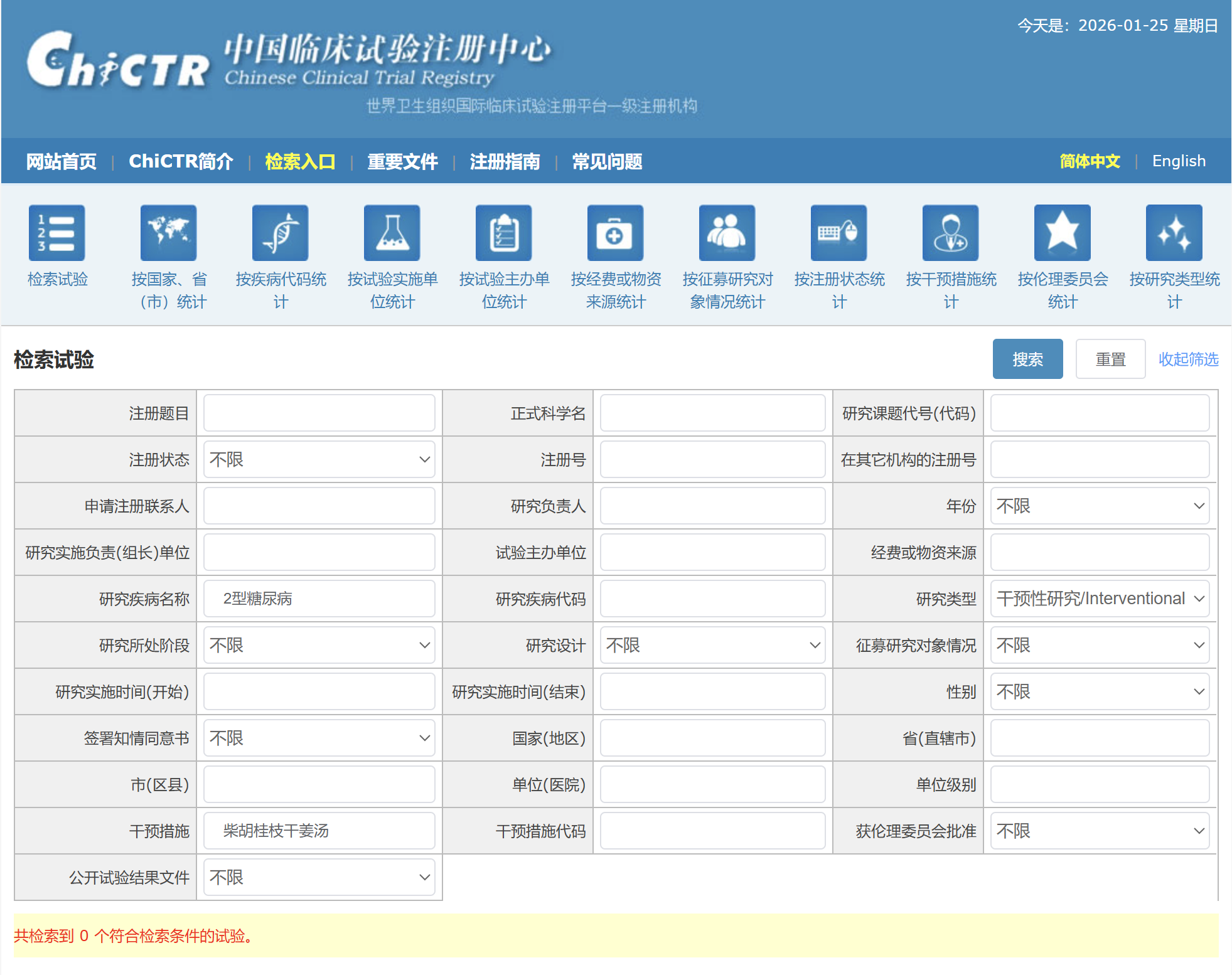


# Supplementary Material S3. Meta regression

## Meta regression of HbA1c for CHGZGJT combined with conventional treatment vs. conventional treatment


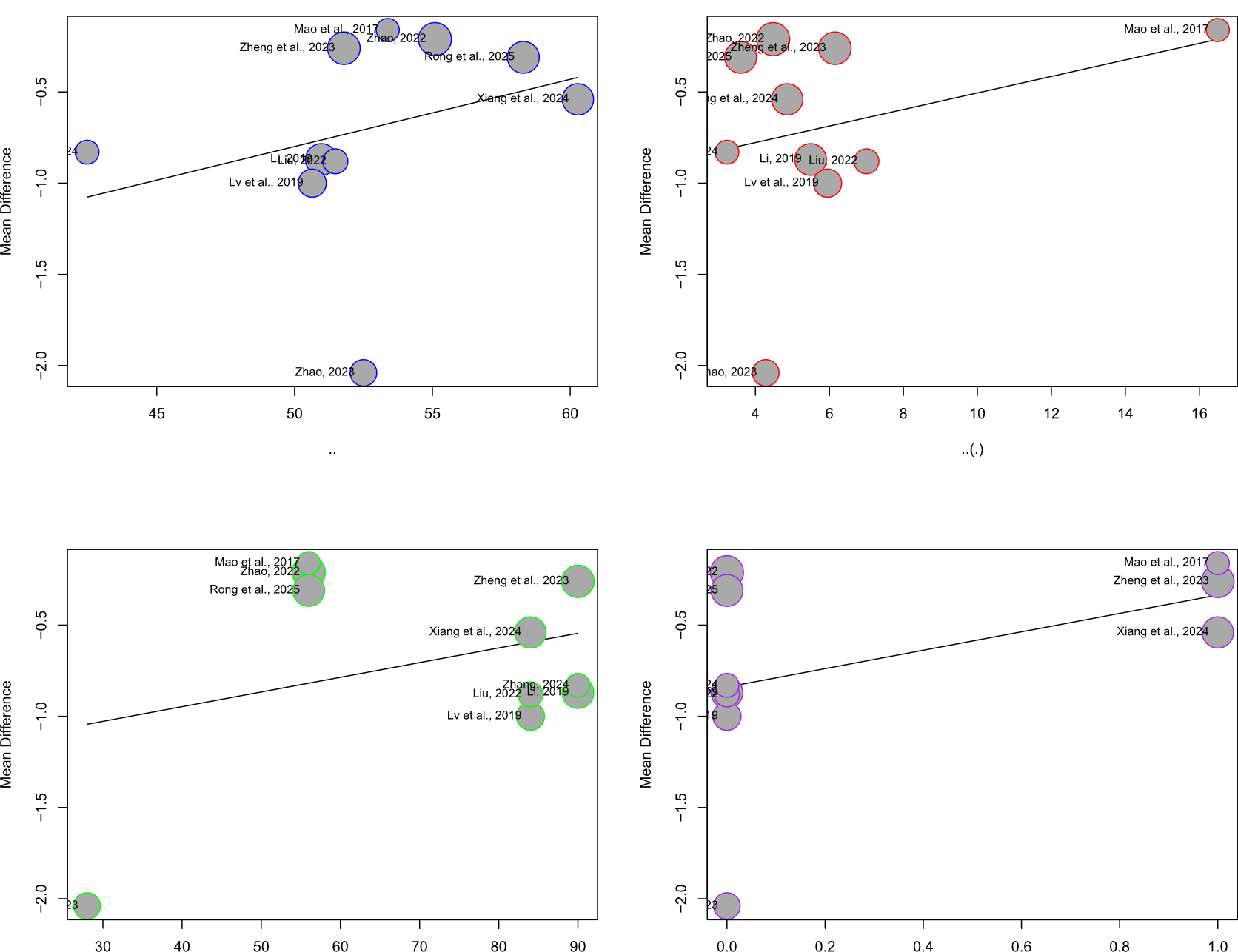


## Meta regression of FPG for CHGZGJT combined with conventional treatment vs. conventional treatment


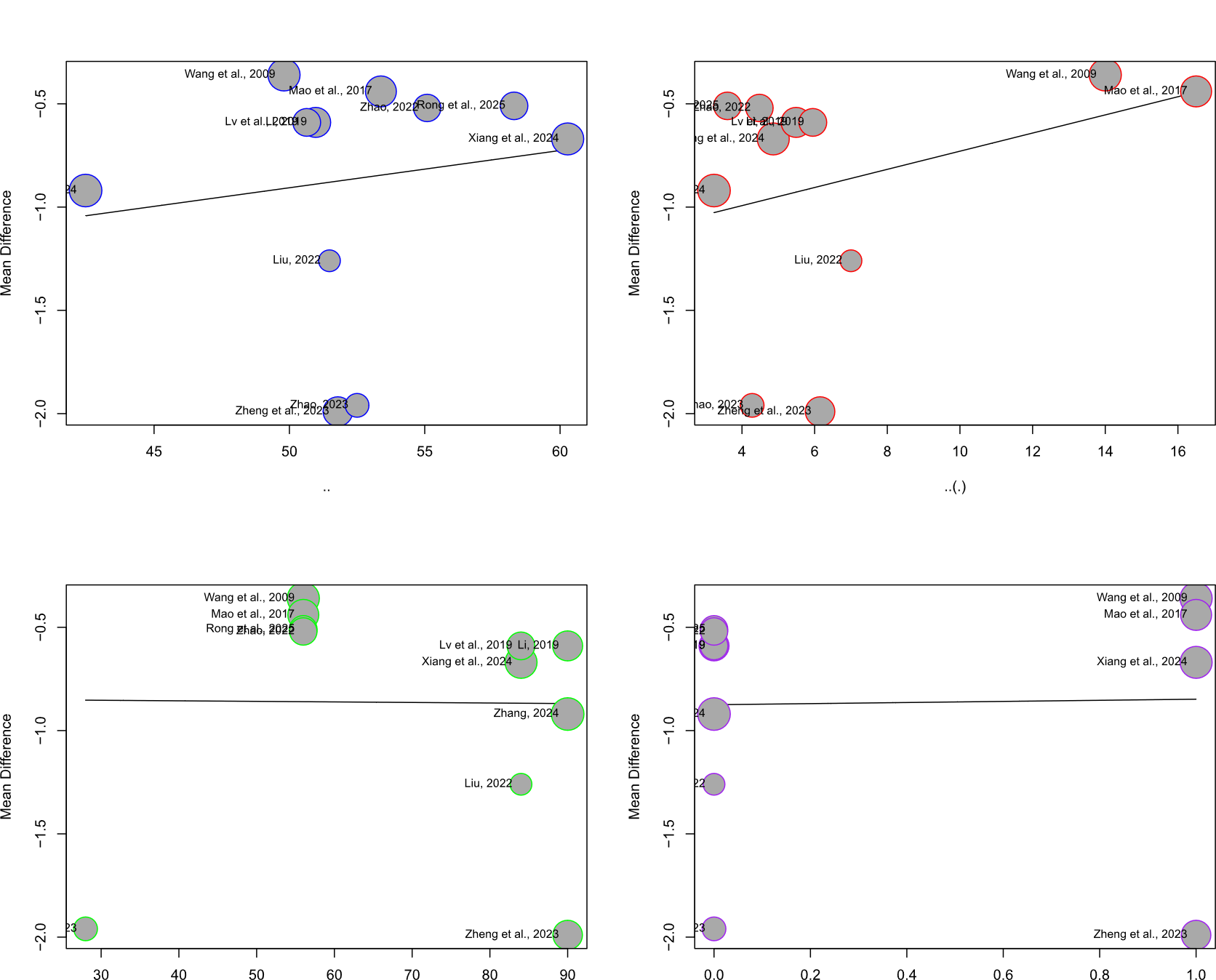


## Meta regression of 2hPG for CHGZGJT combined with conventional treatment vs. conventional treatment


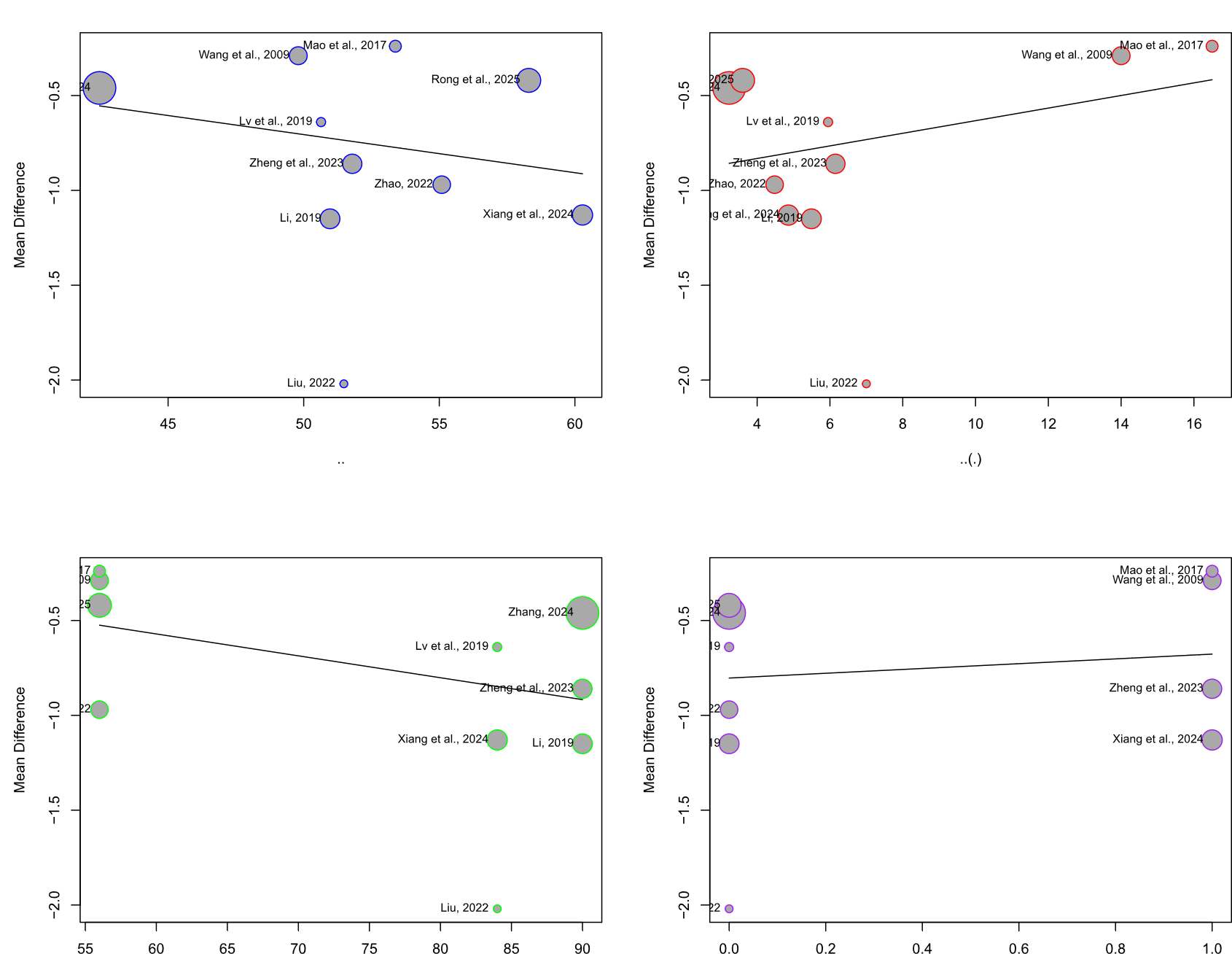


# Supplementary Material S4. Subgroup analysis

## Subgroup analysis of HbA1c for CHGZGJT combined with conventional treatment vs. conventional treatment


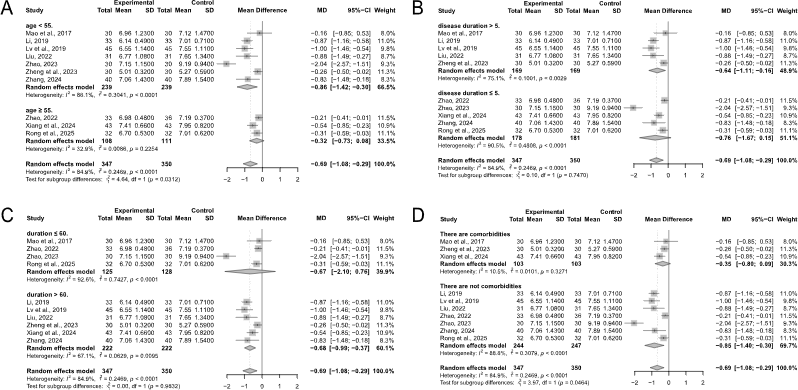


## Subgroup analysis of FPG for CHGZGJT combined with conventional treatment vs. conventional treatment


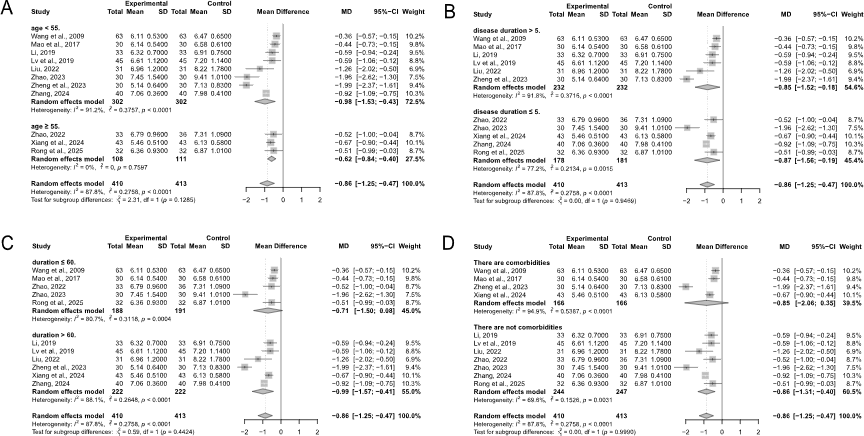


## Subgroup analysis of 2hPG for CHGZGJT combined with conventional treatment vs. conventional treatment


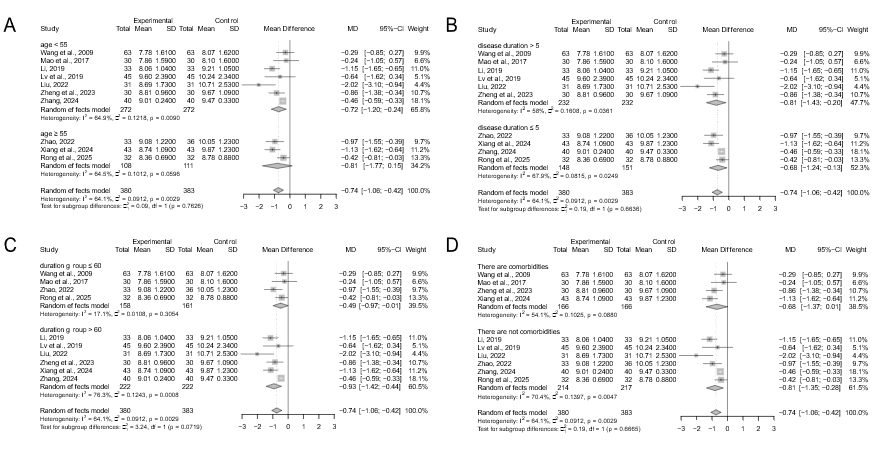


# Supplementary Material S5. Sensitivity analysis

1. HbA1c


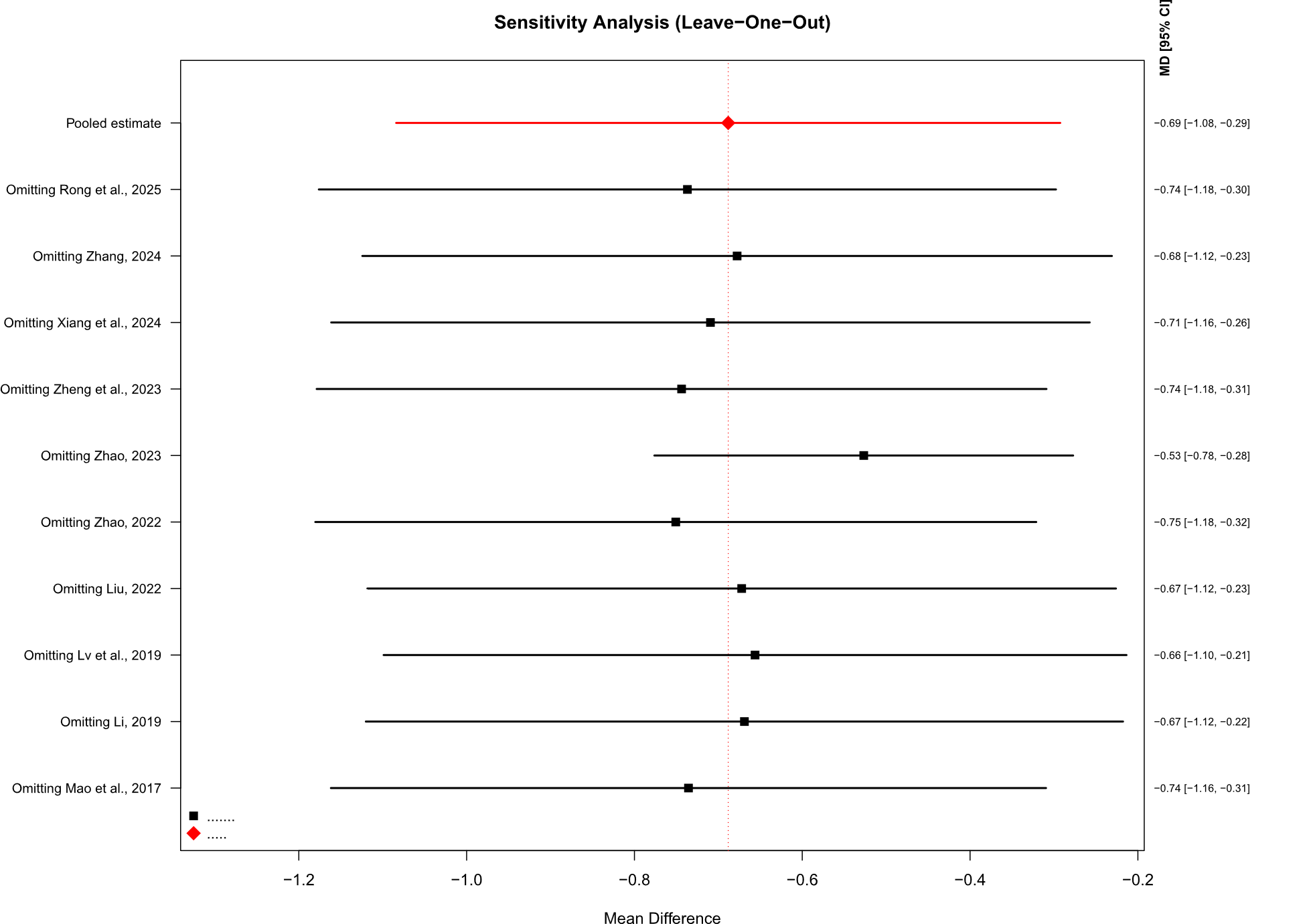


1. FPG


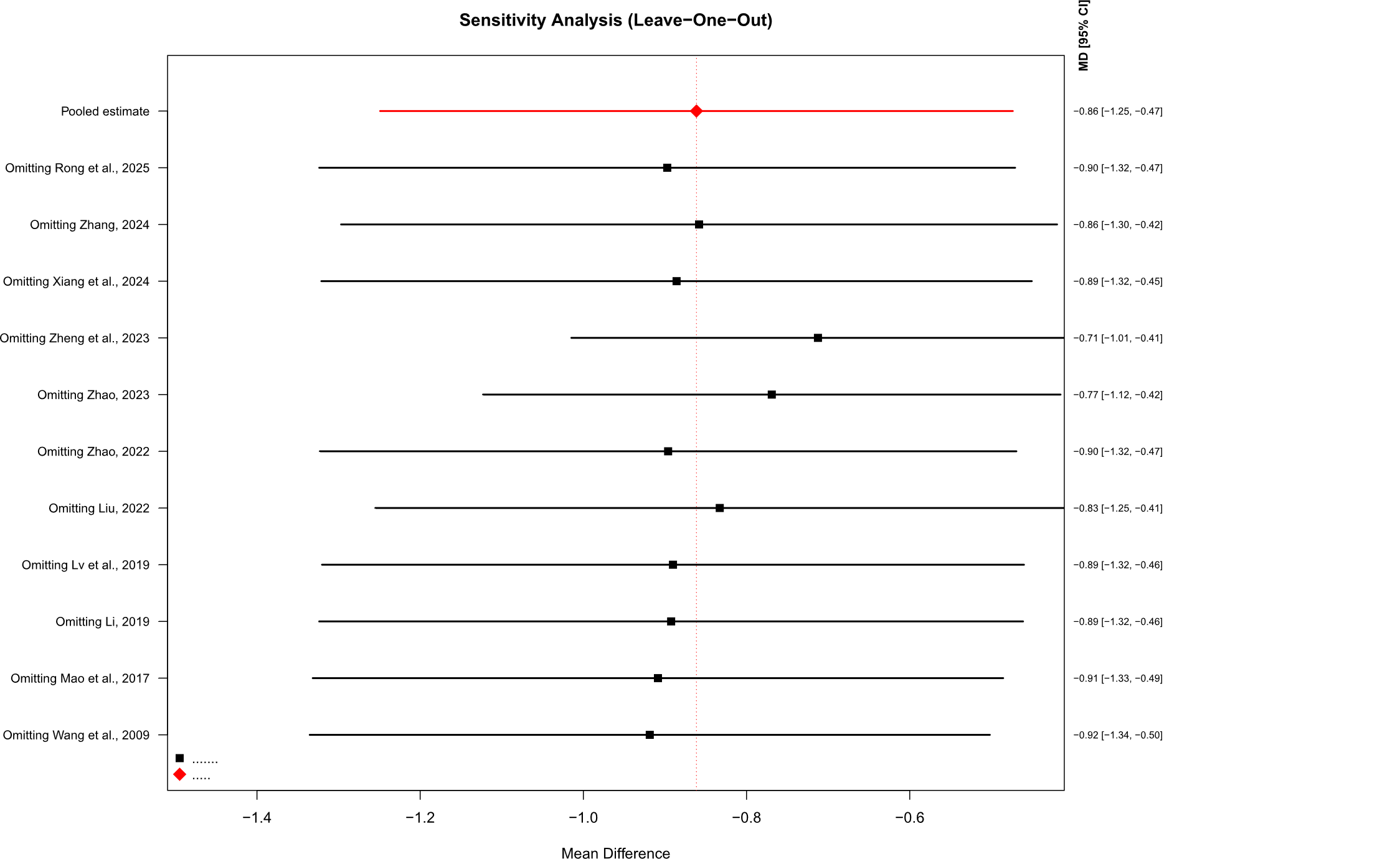


(C) 2hPG


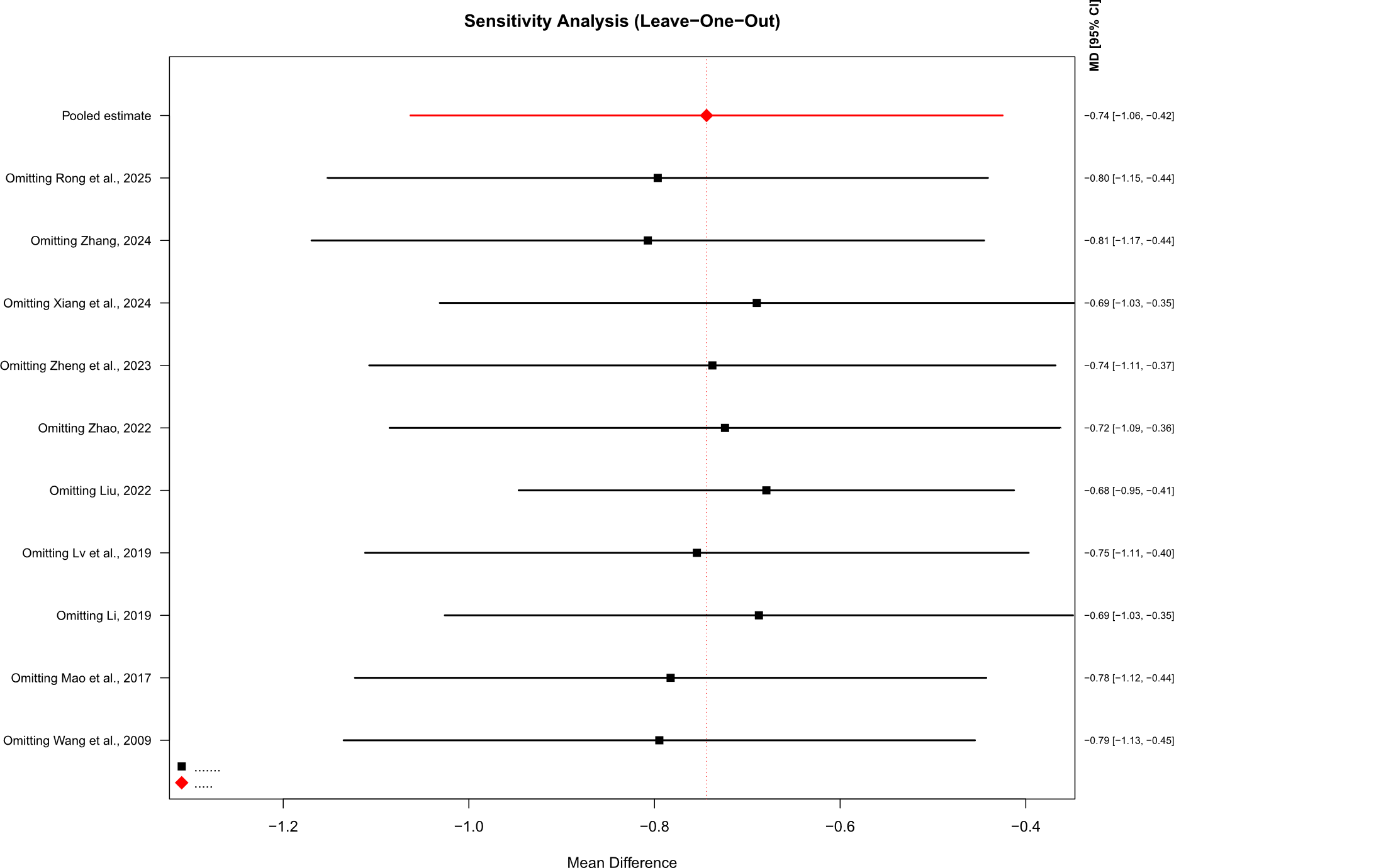


(D) FINS


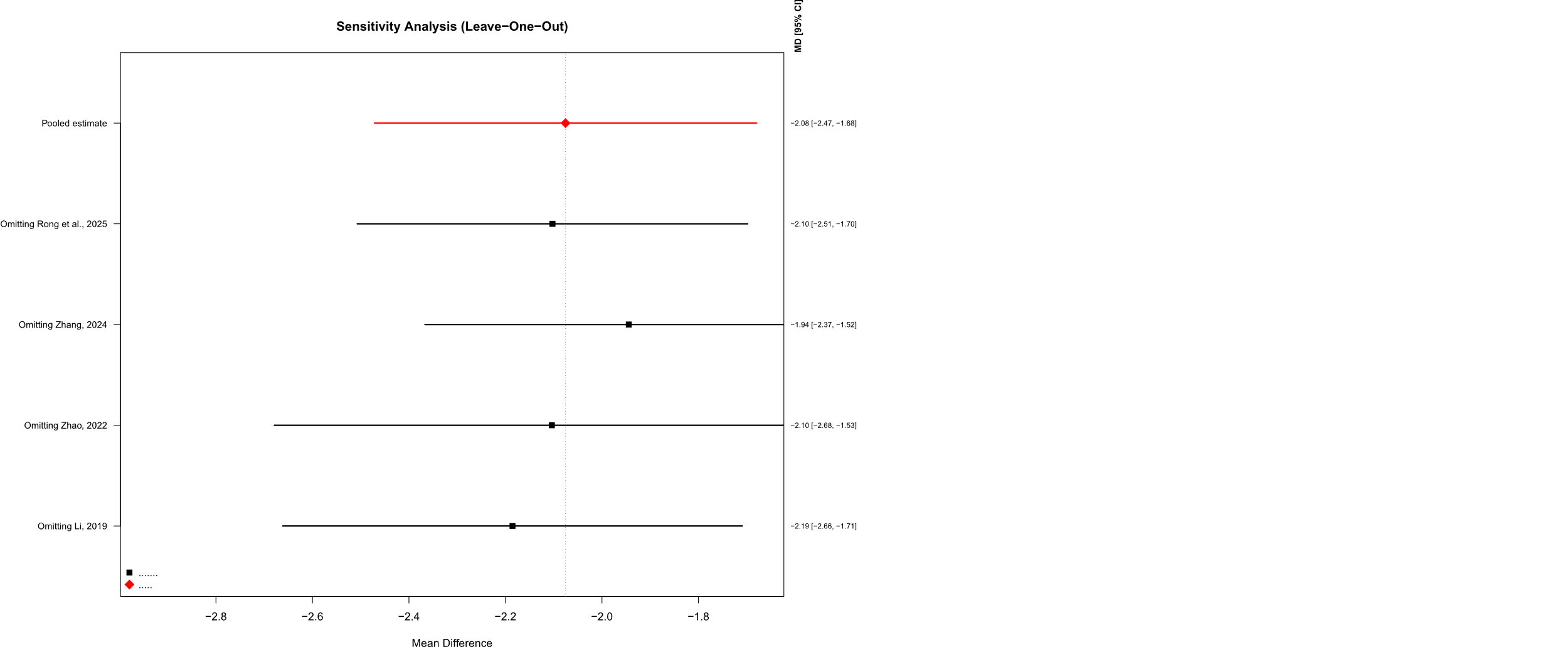


(E) HOMA-IR


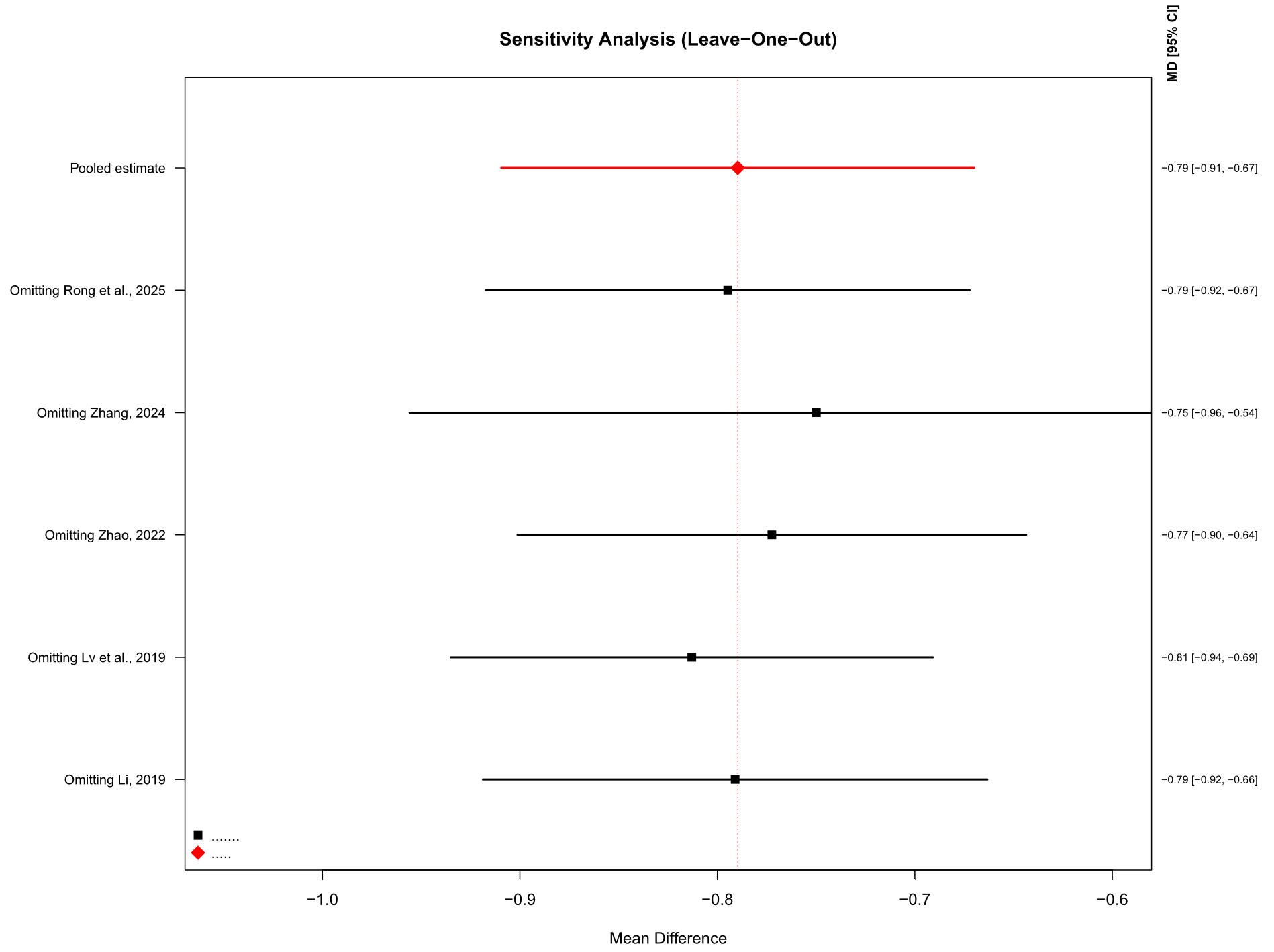


(F) HOMA-β


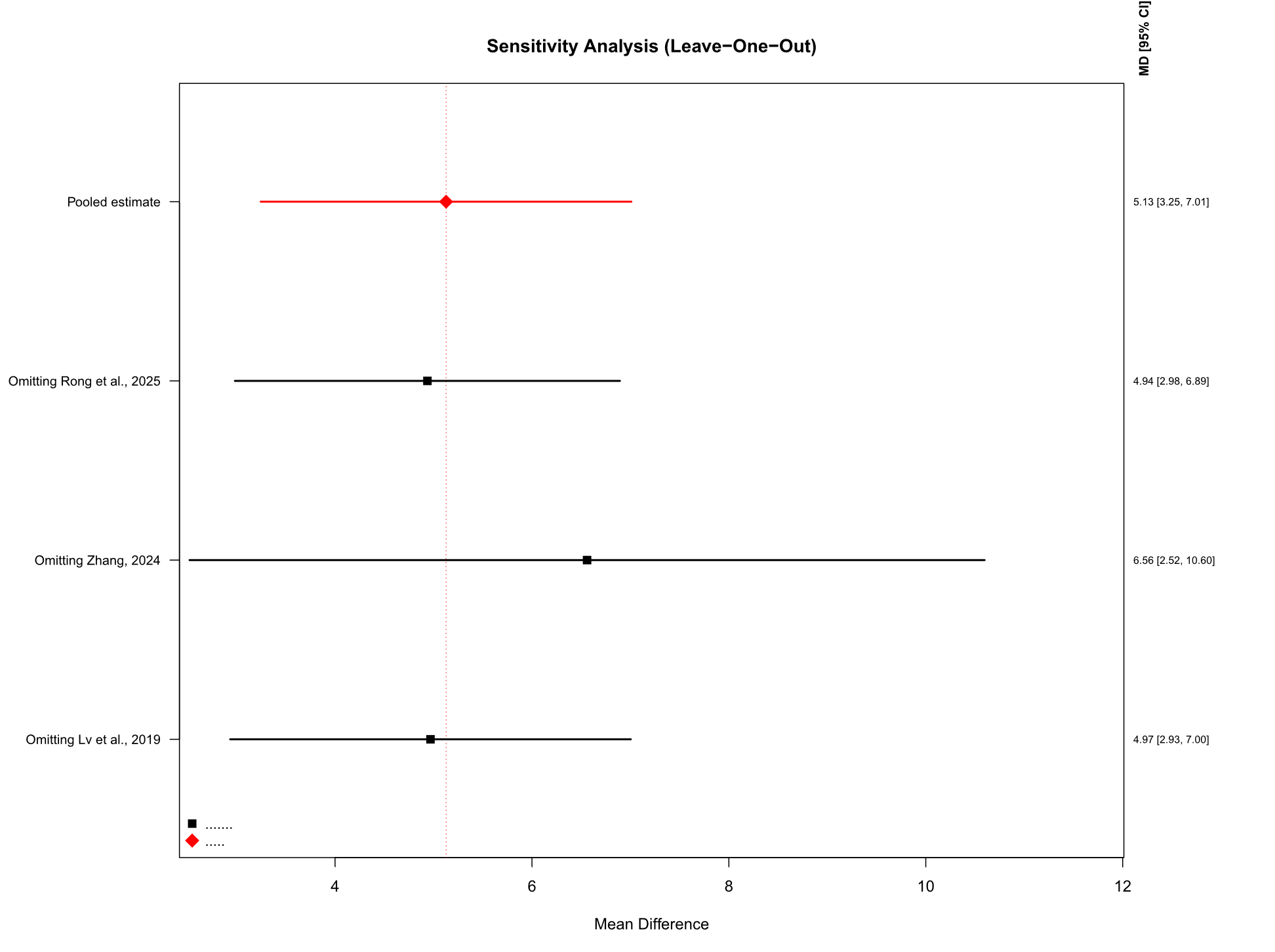


(G) TC;


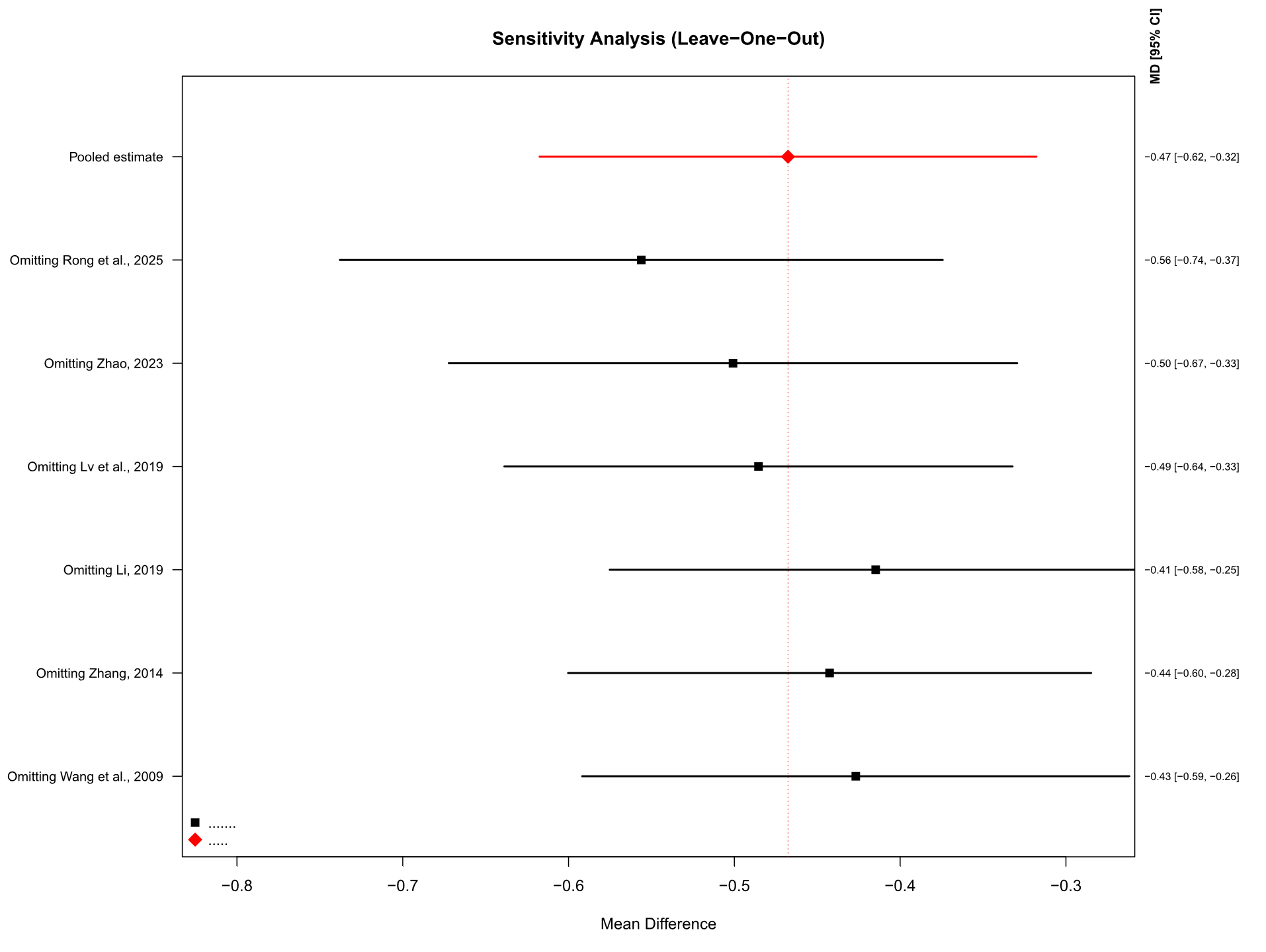


(H) TG


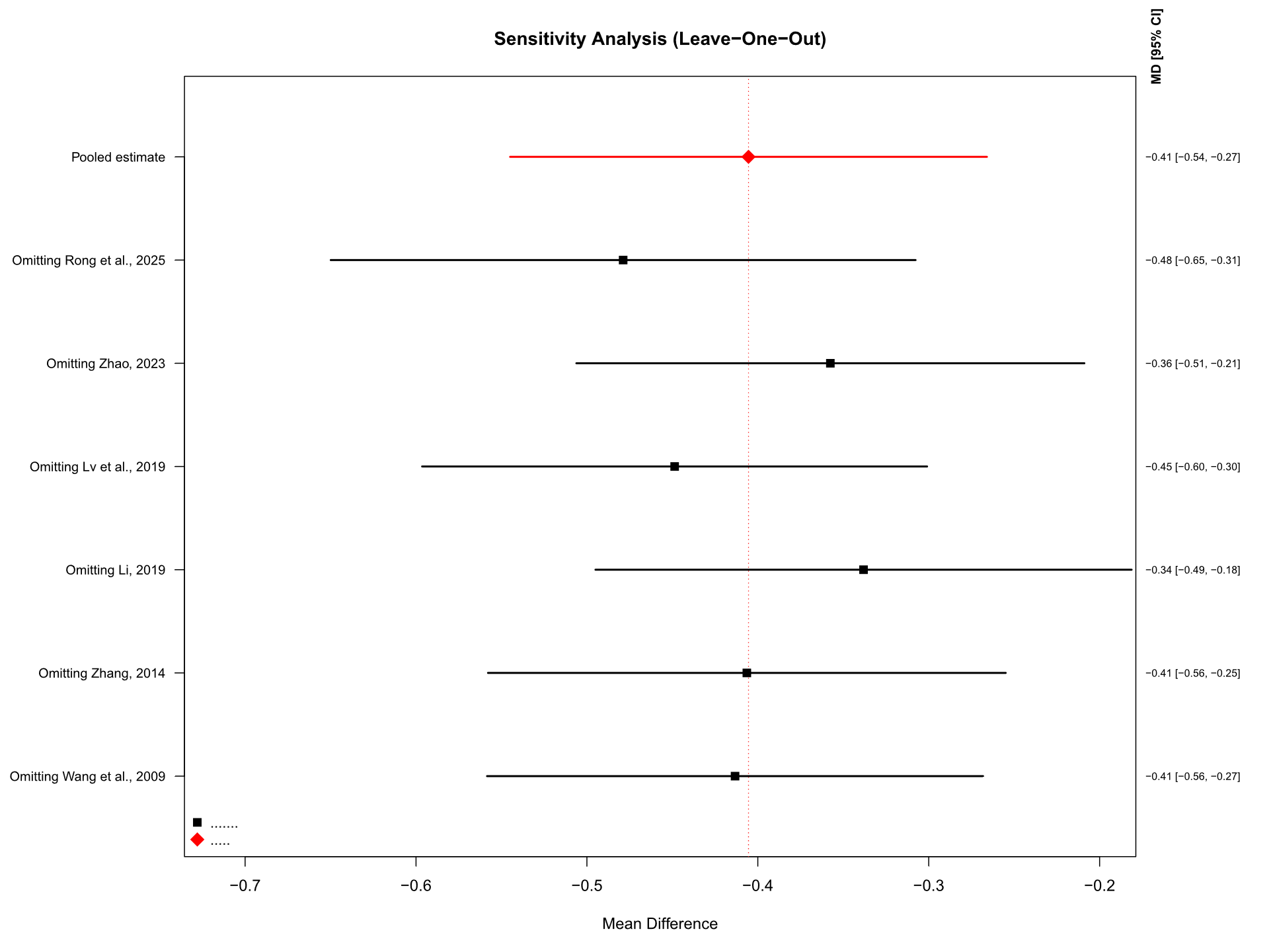


(I) LDL-C


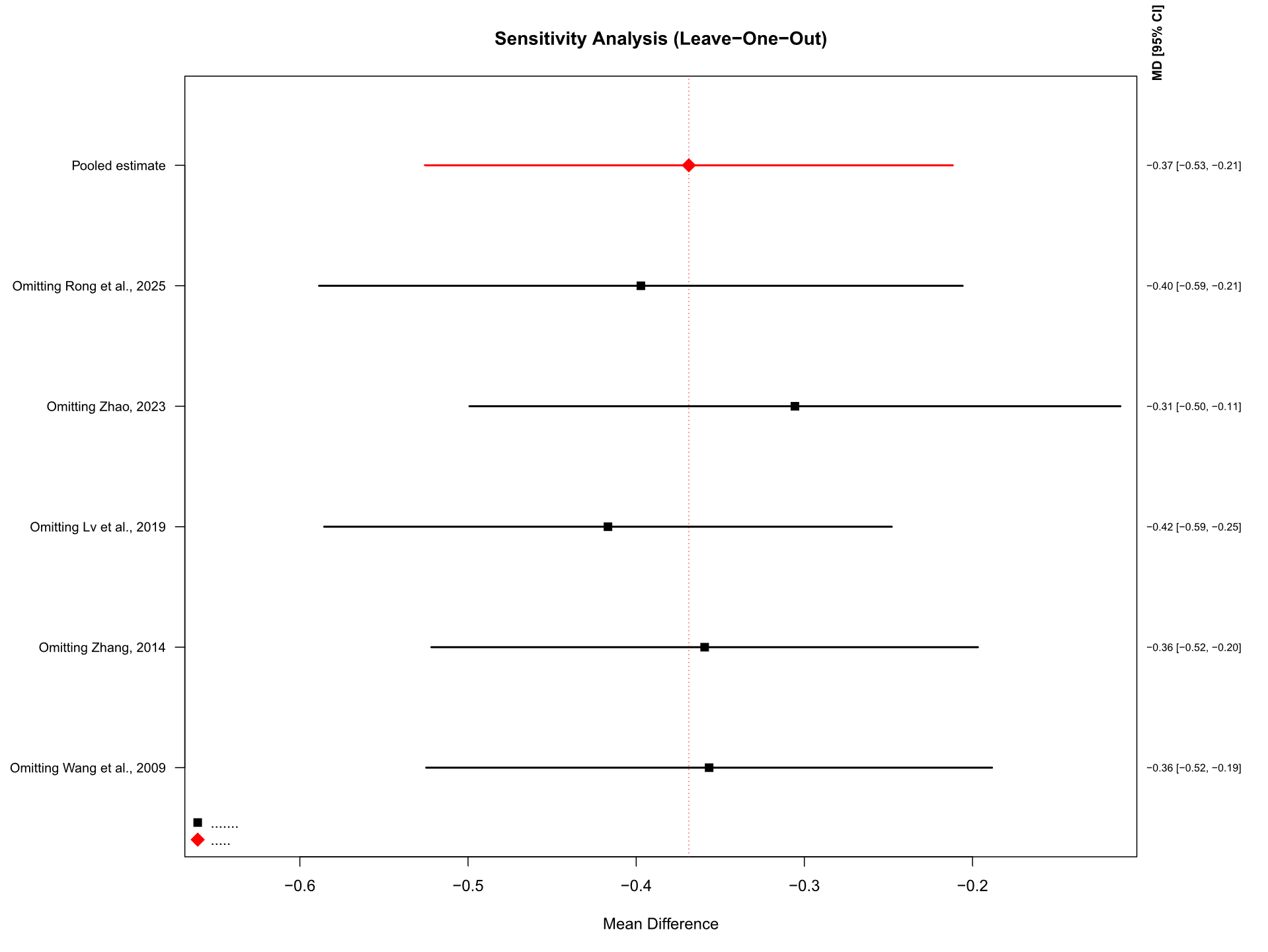


(J) HDL-C


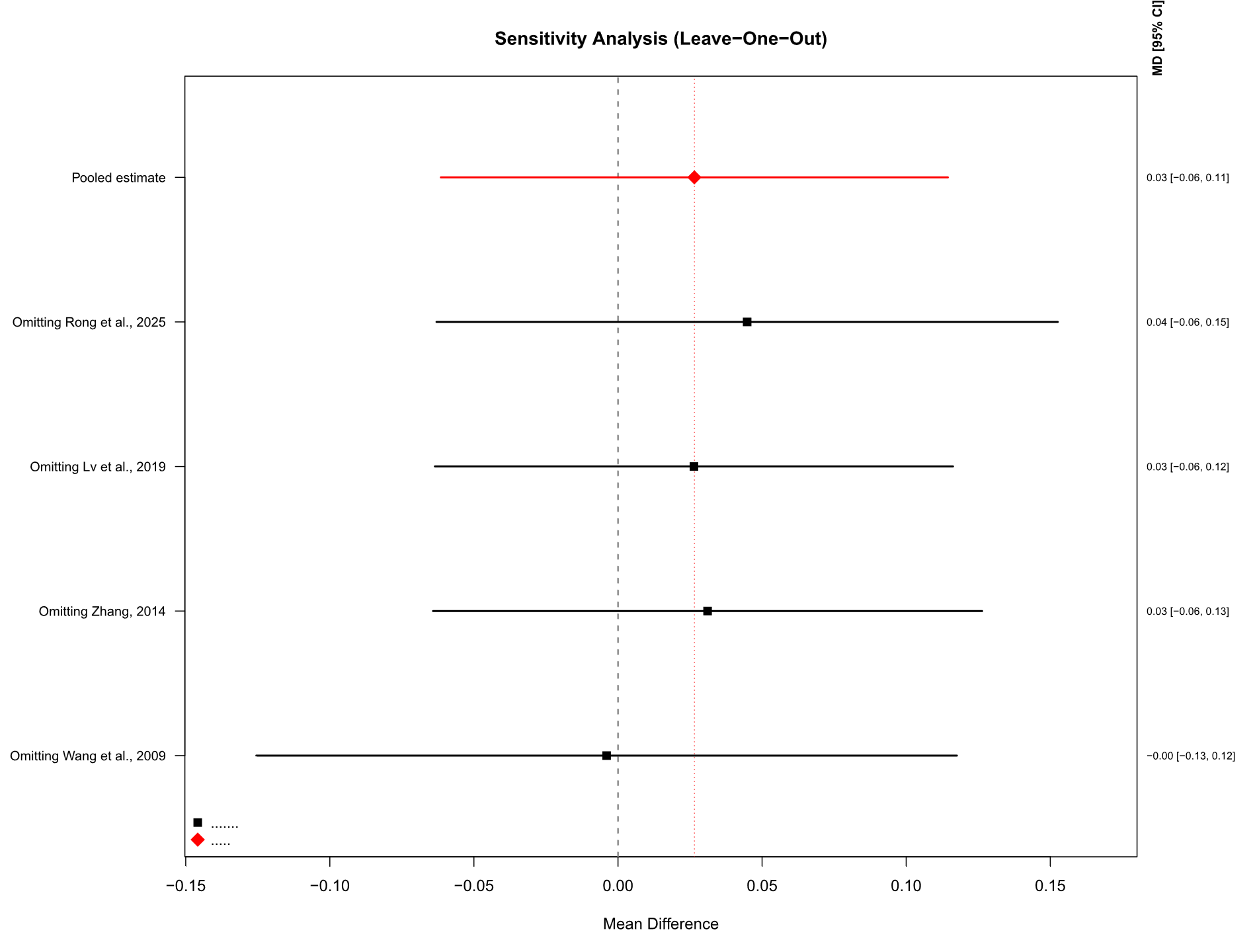


(K) CRP


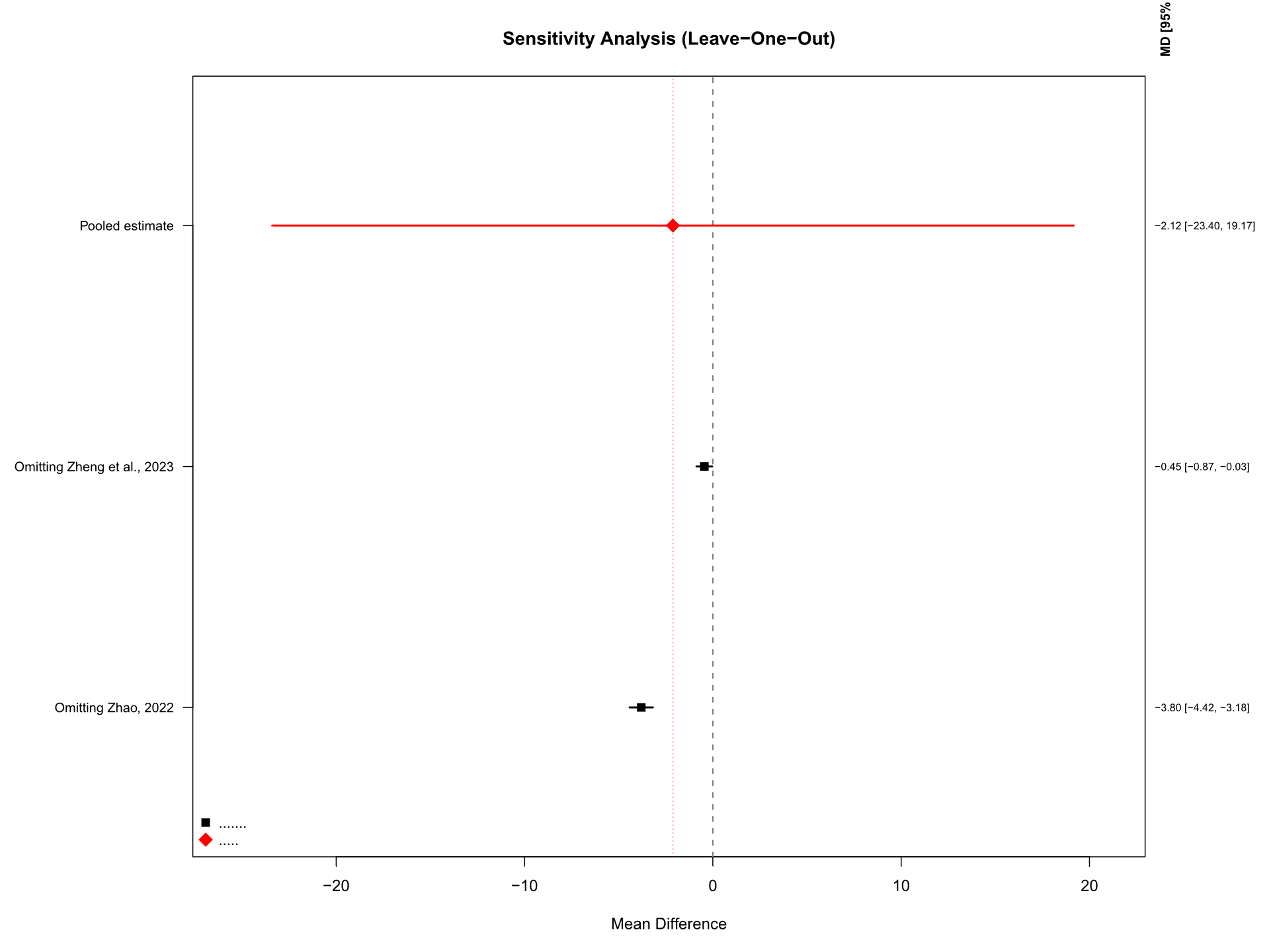


(L) Overall effective rate


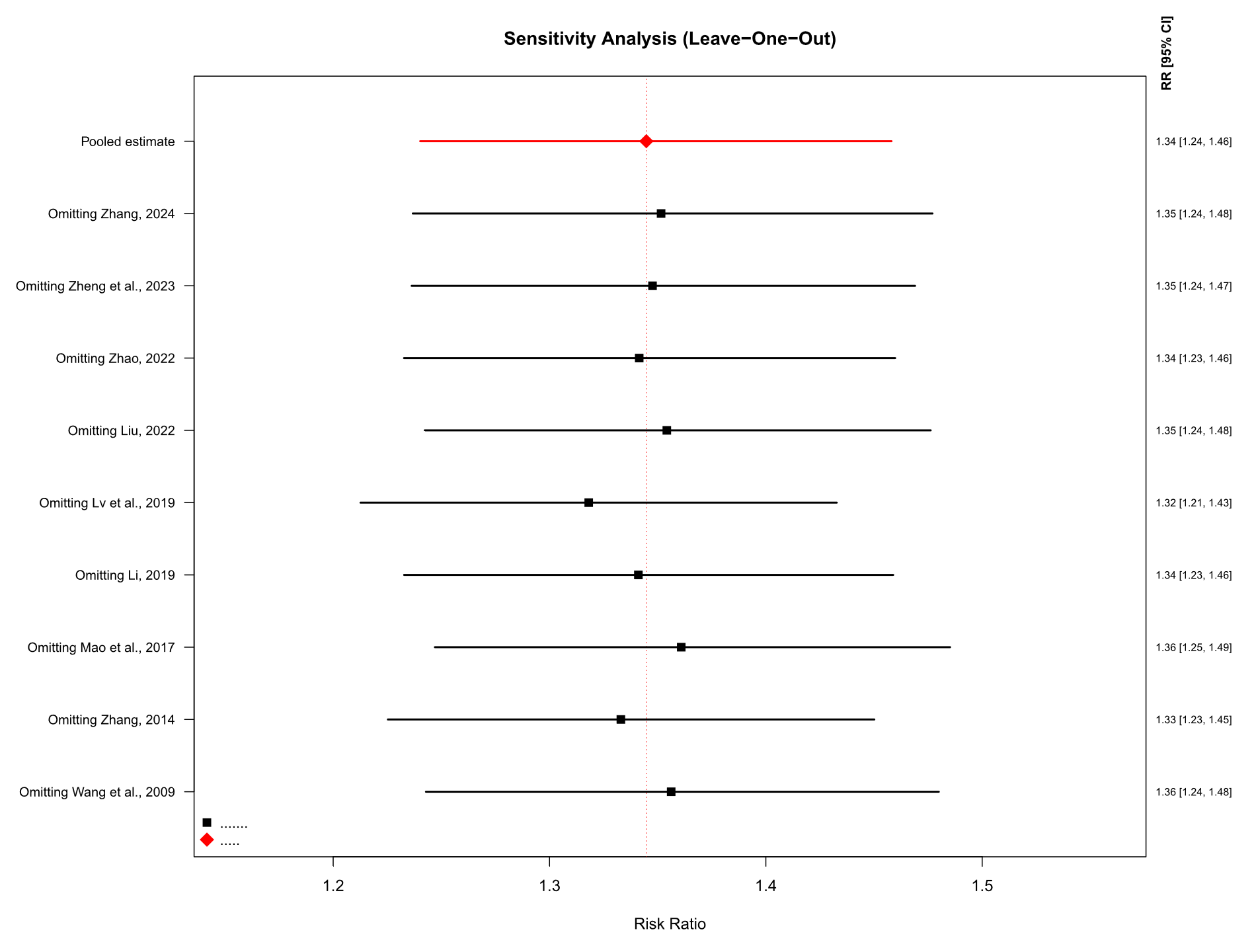


(M) Adverse events rate


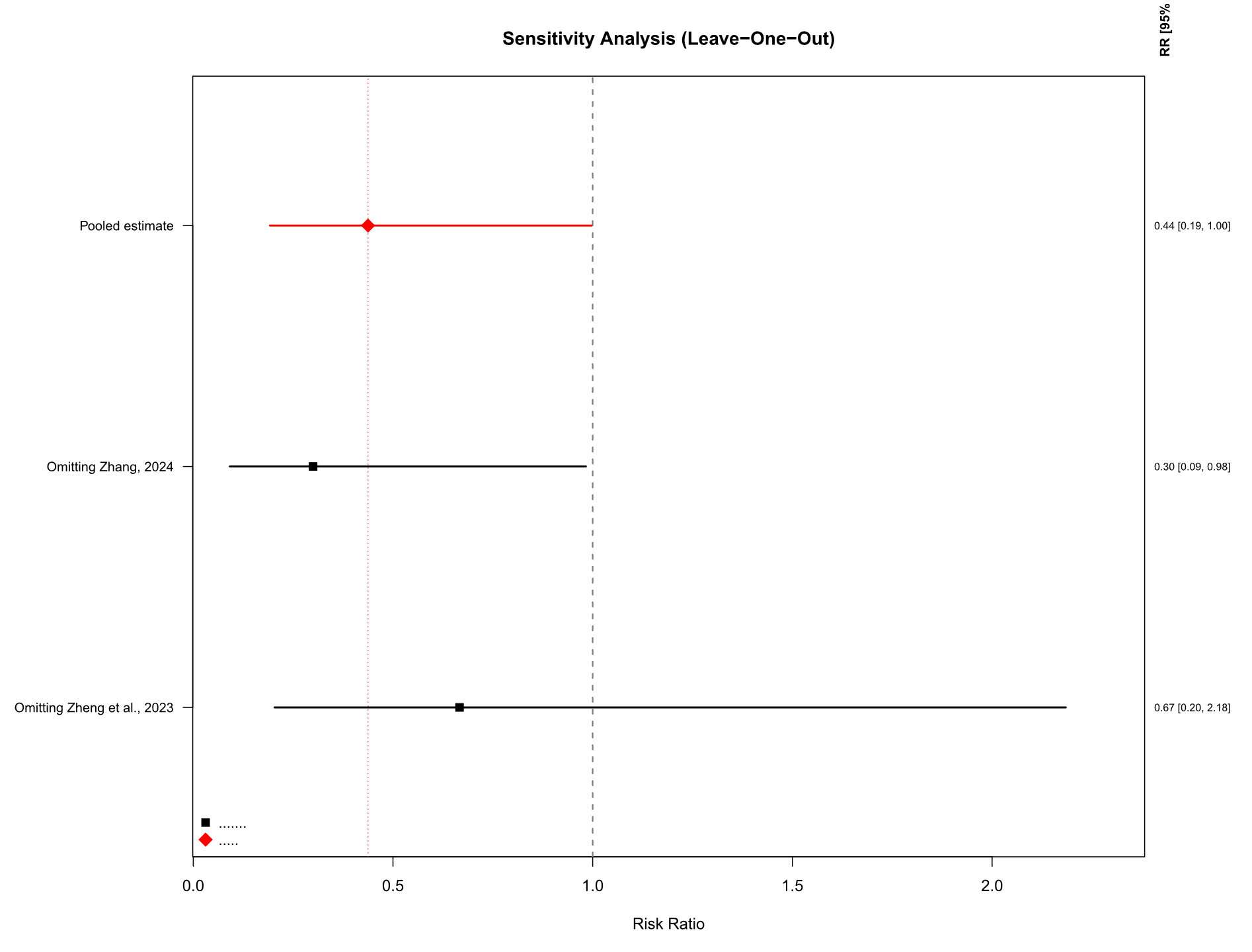


# Supplementary Material S6. Adverse events and safety monitoring reported in the included studies

| **Study** | **Adverse-event reporting** | **Safety parameters monitored** | **Adverse events – intervention group** | **Adverse events – control group** | **Severity / outcome** |
| --- | --- | --- | --- | --- | --- |
| Li, 2019 | Yes (narrative) | Complete blood count, urinalysis, stool routine, liver and kidney function, electrocardiogram | 1 case of mild nausea with abdominal distension | None reported | Mild and self-limiting (resolved within 2 days); all laboratory and ECG parameters normal in both groups |
| Lv et al., 2019 | Yes (narrative) | Adverse-event monitoring | None | None | No adverse reaction in either group |
| Zhao, 2022 | Yes (narrative) | Vital signs (temperature, blood pressure, respiration, heart rate), complete blood count, urinalysis, stool routine, liver and kidney function, electrocardiogram | None reported | None reported | All parameters normal before and after treatment in both groups |
| Zheng et al., 2023 | Yes (quantified) | Adverse-event monitoring | 3/30: gastrointestinal reaction (1), drowsiness (1), dry mouth (1) | 10/30: gastrointestinal reaction (3), fatigue (2), drowsiness (3), dry mouth (2) | All mild; fewer events in the intervention group |
| Zhang, 2024 | Yes (quantified) | Complete blood count, liver and kidney function | 4/40 (10.0%): constipation (1), rash (1), insomnia (2) | 6/40 (15.0%): constipation (2), rash (1), insomnia (3) | All mild; laboratory tests normal; fewer events in the intervention group |
| Rong et al., 2025 | Yes (narrative) | Complete blood count, liver and kidney function | None | None | No adverse reaction in either group; laboratory tests normal |

# Supplementary Material S7. Restricted analysis: influence of the two non-randomized controlled studies (Lv et al., 2019; Liu, 2022) on the pooled estimates

| **Outcome** | **Primary analysis: all eligible studies (10 RCTs + 2 CCTs)** | **Sensitivity analysis: 2 non-randomized studies excluded (RCTs only)** | **Conclusion** |
| --- | --- | --- | --- |
| HbA1c (%) | MD −0.69 (−1.08, −0.29); 10 studies, n=697; I²=84.9% | MD −0.63 (−1.14, −0.13); 8 RCTs, n=545; I²=86.8% | Direction and significance unchanged |
| FPG (mmol/L) | MD −0.86 (−1.25, −0.47); 11 studies, n=823; I²=87.8% | MD −0.86 (−1.34, −0.38); 9 RCTs, n=671; I²=90.0% | Point estimate identical; unchanged |
| 2-h postprandial glucose (mmol/L) | MD −0.74 (−1.06, −0.42); 10 studies, n=763; I²=64.1% | MD −0.68 (−0.98, −0.38); 8 RCTs, n=611; I²=61.0% | Direction and significance unchanged |
| HOMA-IR | MD −0.79 (−0.91, −0.67); 5 studies, n=369; I²=4.6% | MD −0.81 (−0.94, −0.69); 4 RCTs, n=279; I²=0% | Unchanged (slightly stronger) |
| HOMA-β | MD 5.13 (3.25, 7.01); 3 studies, n=234; I²=0% | MD 4.97 (2.93, 7.00); 2 RCTs, n=144; I²=0% | Direction and significance unchanged |
| Total cholesterol (mmol/L) | MD −0.47 (−0.62, −0.32); 6 studies, n=466; I²=40.4% | MD −0.49 (−0.64, −0.33); 5 RCTs, n=376; I²=44.6% | Unchanged (slightly stronger) |
| Triglycerides (mmol/L) | MD −0.41 (−0.54, −0.27); 6 studies, n=466; I²=49.4% | MD −0.45 (−0.60, −0.30); 5 RCTs, n=376; I²=41.5% | Unchanged (slightly stronger) |
| LDL-cholesterol (mmol/L) | MD −0.37 (−0.53, −0.21); 5 studies, n=400; I²=0% | MD −0.42 (−0.59, −0.25); 4 RCTs, n=310; I²=0% | Unchanged (slightly stronger) |
| HDL-cholesterol (mmol/L) | MD 0.03 (−0.06, 0.11); 4 studies, n=340; I²=0% | MD 0.03 (−0.06, 0.12); 3 RCTs, n=250; I²=0% | Non-significant in both; unchanged |
| Overall (clinical) effective rate | RR 1.34 (1.24, 1.46); 9 studies, n=673; I²=0% | RR 1.33 (1.21, 1.45); 7 RCTs, n=521; I²=0% | Direction and significance unchanged |

# Supplementary Material S8. Summary of findings and GRADE certainty of the evidence for CHGZGJT combined with conventional therapy versus conventional therapy alone in type 2 diabetes mellitus

**Certainty key:** ●●●● High ●●●○ Moderate ●●○○ Low ●○○○ Very low.

| **Outcome (post-treatment)** | **N participants (studies)** | **Pooled effect (95% CI)** | **Certainty (GRADE)** | **Comments / reasons for downgrading** |
| --- | --- | --- | --- | --- |
| ***Glucose metabolism*** | | | | |
| Glycated haemoglobin, HbA1c (%) | 697 (10) | MD −0.69 (−1.08 to −0.29) | ●●○○  **Low** | a, b |
| Fasting plasma glucose (mmol/L) | 823 (11) | MD −0.86 (−1.25 to −0.47) | ●●○○  **Low** | a, b |
| 2-h postprandial glucose (mmol/L) | 763 (10) | MD −0.74 (−1.06 to −0.42) | ●●○○  **Low** | a, b |
| ***Insulin function*** | | | | |
| Fasting insulin, FINS (µIU/mL) | 279 (4) | MD −2.08 (−2.47 to −1.68) | ●●●○  **Moderate** | a, e |
| Insulin resistance, HOMA-IR | 369 (5) | MD −0.79 (−0.91 to −0.67) | ●●●○  **Moderate** | a, e |
| β-cell function, HOMA-β | 234 (3) | MD 5.13 (3.25 to 7.01) | ●●○○  **Low** | a, c |
| ***Lipid metabolism*** | | | | |
| Total cholesterol (mmol/L) | 466 (6) | MD −0.47 (−0.62 to −0.32) | ●●●○  **Moderate** | a |
| Triglycerides (mmol/L) | 466 (6) | MD −0.41 (−0.54 to −0.27) | ●●●○  **Moderate** | a |
| LDL-cholesterol (mmol/L) | 400 (5) | MD −0.37 (−0.53 to −0.21) | ●●●○  **Moderate** | a |
| HDL-cholesterol (mmol/L) | 340 (4) | MD 0.03 (−0.06 to 0.11) | ●●○○  **Low** | a, c |
| ***Secondary outcomes*** | | | | |
| C-reactive protein (mg/L) | 129 (2) | MD −2.12 (−23.40 to 19.17) | ●○○○  **Very low** | a, b, c |
| Overall (clinical) effective rate | 673 (9) | RR 1.34 (1.24 to 1.46) | ●●○○  **Low** | a, d |
| Adverse events | 140 (2) | RR 0.44 (0.19 to 1.00) | ●●○○  **Low** | a, c |

**Footnotes**

**a. Risk of bias —** the evidence base comprised ten randomized controlled trials (all rated “some concerns” on RoB 2, because allocation concealment and blinding of participants, personnel and outcome assessors were not reported and no trial was prospectively registered or had a pre-published protocol) together with two non-randomized controlled studies (both rated moderate risk of bias on ROBINS-I). Downgraded one level. A pre-specified sensitivity analysis excluding the two non-randomized studies left the direction and statistical significance of all pooled estimates unchanged (Supplementary Material S7).

**b. Inconsistency —** substantial-to-considerable unexplained statistical heterogeneity (I² > 50%) that pre-specified subgroup analyses and meta-regression did not fully explain. Downgraded one level (for C-reactive protein, I² = 98.7%).

**c. Imprecision —** few studies and/or participants and/or a 95% confidence interval compatible with no important effect (optimal information size not met). Downgraded one level.

**d.** The overall (clinical) effective rate is a subjective composite outcome that is particularly susceptible to performance and detection bias in the absence of blinding.

**e.** The pooled estimate was obtained after exclusion of one short-duration (4-week) study identified by leave-one-out sensitivity analysis as the source of heterogeneity; residual heterogeneity was low (I² ≤ 15%), so the certainty was not further downgraded for inconsistency.

***Abbreviations:*** *CI, confidence interval; MD, mean difference; RR, risk ratio; HOMA-IR/β, homeostasis model assessment of insulin resistance/β-cell function.*

# Supplementary Material S9. Egger’s test of HbA1c, FPG and 2hPG

(A) HbA1c: Test result: t = -2.17, df = 8, p-value = 0.0622; Bias estimate: -1.7414 (SE = 2.0930)

(B) FPG: Test result: t = -0.83, df = 9, p-value = 0.4269; Bias estimate: -1.7414 (SE = 2.0930)

(C) 2hPG: Test result: t = -2.08, df = 8, p-value = 0.0707; Bias estimate: -1.4680 (SE = 0.7045)
